# UFPS: A unified framework for partially annotated federated segmentation in heterogeneous data distribution

**DOI:** 10.1016/j.patter.2024.100917

**Published:** 2024-01-25

**Authors:** Le Jiang, Li Yan Ma, Tie Yong Zeng, Shi Hui Ying

**Affiliations:** 1 School of Computer Engineering and Science, Shanghai University, Shanghai, China; 2 Department of Mathematics, Chinese University of Hong Kong, Hongkong, China; 3 Department of Mathematics, Shanghai University, Shanghai, China

**Keywords:** federated learning, medical image segmentation, partial label

## Abstract

Partially supervised segmentation is a label-saving method based on datasets with fractional classes labeled and intersectant. Its practical application in real-world medical scenarios is, however, hindered by privacy concerns and data heterogeneity. To address these issues without compromising privacy, federated partially supervised segmentation (FPSS) is formulated in this work. The primary challenges for FPSS are class heterogeneity and client drift. We propose a unified federated partially labeled segmentation (UFPS) framework to segment pixels within all classes for partially annotated datasets by training a comprehensive global model that avoids class collision. Our framework includes unified label learning (ULL) and sparse unified sharpness aware minimization (sUSAM) for class and feature space unification, respectively. Through empirical studies, we find that traditional methods in partially supervised segmentation and federated learning often struggle with class collision when combined. Our extensive experiments on real medical datasets demonstrate better deconflicting and generalization capabilities of UFPS.

## Highlights

• Provided solutions for federated partially supervised segmentation challenges
• Trained a global model via heterogeneous datasets without class intersections
• Demonstrated efficiency of the global model on most classes for all domains

## The bigger picture

Labeling numerous datasets for segmentation tasks is labor intensive and requires expert knowledge. Although techniques exist to address labeling problems, such as partially supervised segmentation, their application to medical data is limited due to data privacy and domain gaps. By adopting federated learning, the proposed framework protects data privacy and increases annotation efficiency of medical imaging segmentation tasks with enhanced generalization ability.

## Graphical abstract

**Figure:**
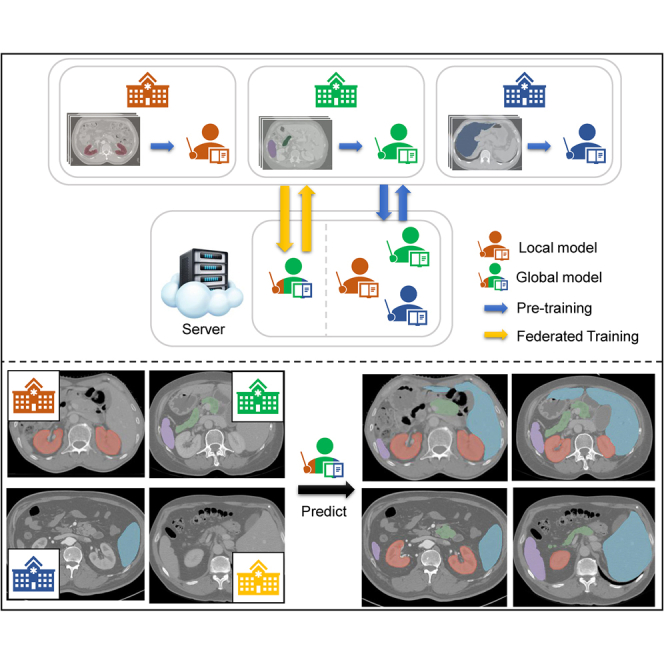

## Teaser

A unified federated partially labeled segmentation framework is presented in this work. The framework provides insights into performing medical data annotation tasks with better generalization across different domains without class collision.

## Introduction

Deep learning techniques^1^ have advanced the field of computer-aided diagnosis,^2^^,^^3^ providing effective tools for clinicians. These techniques often rely on large-scale data with abundant diversity to achieve accurate results.^4^^,^^5^ However, it requires specialized knowledge and significant effort to collect annotations for medical data, especially for dense pixel-level tasks. In response to this challenge, partially supervised segmentation (PSS)^6^^,^^7^^,^^8^^,^^9^ has emerged as a label-saving approach. Unlike traditional learning paradigms, PSS aims to segment datasets with only a subset of classes annotated for each one. Importantly, there is little to no overlap in the labeled classes between datasets in PSS, while the union includes all classes simultaneously.

Existing works on PSS^6^^,^^7^^,^^8^^,^^9^ mainly depend on centralized datasets, which do not comply with privacy regulations in real-world medical applications.^10^ Federated learning (FL),^11^ a distributed learning framework, can serve as a promising solution to this challenge. It allows multiple clients, such as hospitals or apartments, to cooperate in training a global model without sharing their data by aggregating model weights or gradients. However, utilizing partially annotated labels to train a global segmentation model in the FL scenario is underexplored. In this work, we propose an extension of the PSS formulation to an FL setting, called federated PSS (FPSS). Apart from low demands for label integrity and protection for data privacy, this setting also has the potential to enhance model generalization through knowledge communication.

**Figure 1:**
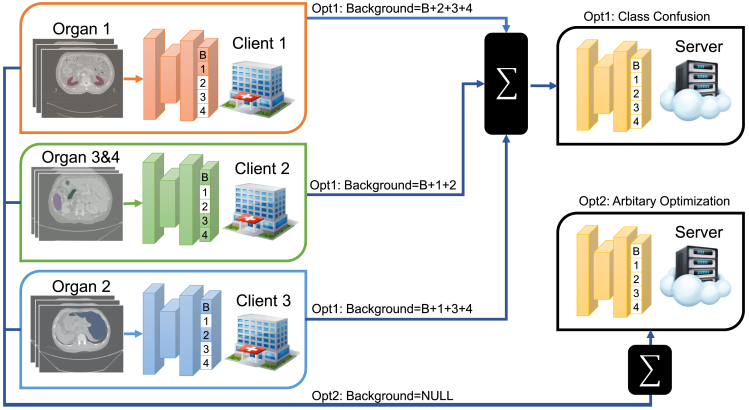
Illustration of solutions in centralized learning to the class heterogeneity problem for FPSS on CT images Each client has only two or three classes annotated, which are colored in the segmentation head. “Option. 1” merges unannotated classes into the background class to calculate the loss. “Opt 2” only uses the foreground class(es) to calculate the loss.

The learning process for FPSS confronts two major challenges: class heterogeneity and client drift. The class heterogeneity problem arises from inconsistency of annotated classes among clients. To illustrate solutions to this problem in centralized learning, we provide an example in Figure 1. When all unannotated classes are merged into the background class to calculate loss functions (Opt 1), the global model suffers severe class conflict. It comes down to the fact that foreground annotations for each client are mistaken as background ones in the setting of other clients. When clients only use annotated classes to calculate loss (Opt 2), foreground channels without supervision can be optimized to any false direction. One feasible solution for the class conflict issue is to aggregate part of the whole model globally and keep the rest of parts local; e.g., excluding the segmentation head from global model aggregation. However, some classes may be relevant in the medical field, such as relative organ position in a computed tomography (CT) image.^12^^,^^13^^,^^14^ This approach may hinder potential interactions between classes during training and requires extra computational cost during evaluation.

In FL, client drift is caused by the assumption that data across clients are in non-independent identically distribution (non-IID).^15^^,^^16^ This drift can be ascribed to various factors in the medical field, such as differences in data collection protocols^17^ or devices,^18^ population diversity,^19^ etc. When weights or gradients with significant divergence between clients are aggregated under the non-IID setting, the global model can be optimized to a suboptimal solution, and the convergence speed can also be decelerated. Previous methods to tackle the problem in FL can be categorized into three major directions: local optimization rectification,^20^^,^^21^^,^^22^^,^^23^^,^^24^^,^^25^^,^^26^ client selection,^27^^,^^28^ and contrastive learning.^29^^,^^30^^,^^31^^,^^32^^,^^33^ While client selection is mainly designed for full-class supervised learning, contrastive learning is computationally expensive, especially for unified federated partially labeled segmentation (UFPS) in the medical field. Although there are lots of works about local optimization rectification, most of them only emphasize optimization, neglecting the importance of local data distribution.

To resolve the problems of class heterogeneity and client drift, we propose a framework called UFPS. It alleviates effects of class heterogeneity by unified label learning (ULL) and ones of client drift by sparse unified sharpness aware minimization (sUSAM).

In FPSS, only a subset of all classes is labeled for each client, and common PSS methods in centralized learning fail to generalize when utilized in the FL scenario. Therefore, to address the class conflict problem and to take advantage of underlying class intersections, ULL adopts a unified labeling approach based on pretrained class-specific teacher models. Because the pretraining step is executed locally, clients can train their models at any time, which is free from communication burden and stability issues in FL.^34^ Different from the traditional pseudolabeling process, to avert concept collision among clients, we filter the intersection part within pretrained teacher models in the background channel. It is the first attempt to incorporate the pseudolabeling idea into FPSS. Thus, the class heterogeneity problem is converted to a noisy label learning issue.

We tackle the noisy label learning issue from global and local perspectives. Since the global model benefits from overall data distribution and class interactions, it can serve as a more reliable source for generating pseudolabels compared with pretrained teacher models. By assigning higher model aggregation weights to clients with high-quality data, the global model is more likely to concentrate on credible knowledge, thus providing improved guidance to local models. On the other hand, the common performance bottleneck of local models is relevant to the coupling between noise and hard classes in pseudolabels. To mitigate this issue, we introduce a loss weight scheduler that helps alleviate the side effects of noise while better fitting hard classes.

As a solution to the non-IID issue, adaptive sharpness aware minimization (ASAM) has been proven as an effective two-step approach in previous work, called federated ASAM (FedASAM).^35^ Despite the good performance of ASAM, because the two steps are both based on the plain local dataset, some sharp directions may deviate from the global optimal path. This is because when each local model is optimized toward the sharpest local direction, certain directions may be relevant to client-specific attributes, thus restricting the generalization ability of the global model. Additionally, the training time of FedASAM is doubled compared with FedAvg. For better generalization, we propose a modified approach called sUSAM, which allows local models to approximate a unified optimization target for all clients through strong data augmentation. The effect of data augmentation is limitedly studied in the field of FL because underlying data information is banned from sharing. Besides, attempts to transfer traditional data augmentation techniques to FL either promote the global model performance marginally through slight augmentation or worsen it through strong augmentation.^35^ By decoupling training data for two steps in ASAM, local models can be free from training instability caused by strong data augmentation. Furthermore, to accelerate the ASAM based framework and avoid overfitting local-specific attributes, we concentrate only on the most essential ascent directions to optimize with while exploring latent directions to enhance the generalization ability of the global model. Through our experiments, we demonstrate that our approach is capable of getting a large margin beyond previous methods. Our contributions can be concluded as follows.

(1) We investigate challenges for FPSS systematically and propose a heterogeneous benchmark based on our solutions.
(2) We propose a UFPS framework for FPSS based on pseudolabeling technology for the first time. Within this framework, we introduce two key components: ULL and sUSAM. They are designed to cope with issues of class heterogeneity and client drift, respectively.
(3) The comprehensive experiments on the benchmark validate the effectiveness of our proposed method.

## Related work

### PSS in the medical domain

Many efforts have been made to conduct PSS in the medical domain. Deep learning (DL) approaches in this context can be categorized into three main branches: prior guided segmentation, index-conditioned segmentation, and pseudolabel-based segmentation.

As one of the *a priori* guided segmentation methods, prior-aware neural network (PaNN)^6^ distills the volume ratio of target organs based on a fully labeled dataset, which may be hard to collect in real-world applications. Partial- and mutual-prior incorporated framework (PRIMP)^36^ utilizes average masks for several groups as *a priori*, but it requires manual preprocessing for start and end slices. These methods can result in inaccurate segmentations in an FL scenario once such priori varies significantly across domains.

Conditional decoder (Cond-dec)^37^ and dynamic on-demand network (DoDNet)^38^ incorporate organ indexes into the network. In Cond-dec, indexes are encoded into hash values and used as additional activations for each layer. DoDNet combines bottleneck features and an index vector to dynamically generate weights and bias for the segmentation head. However, both of them require repetitive forward steps for all organs during the reference process, which is time consuming, especially in the medical domain.

Cross pseudo supervision (CPS),^39^ a pseudolabel-based segmentation method, uses Siamese networks supervising each other to correct potential noises in pseudolabels. Another way in this research field is MS-KD,^40^ which pretrains teacher models based on several datasets, each for one organ, to generate pseudolabels. In multi-teacher single-student knowledge distillation (MS-KD), features from all layers are distilled along with final logits to ease model training using the Kullback-Leibler (KL) loss.^41^ Other methods do not fall into either of these categories. For example, pyramid input pyramid output feature abstraction network (PIPO-FAN)^9^ utilizes multiscale inputs and features to capture details and global context. For all methods mentioned above, the domain gap issue is not taken into consideration, which is common in the medical domain.

### FL

One of the most serious challenges in FL is statistical heterogeneity of decentralized data. To surmount this barrier, numerous works are put forward. For instance, a regularization term between the global model and local models is proposed in FedProx.^20^ Stochastic controlled averaging algorithm (SCAFFOLD)^21^ uses control variants to mitigate local gradient drift. These two methods are limited in highly non-IID scenarios.

Model contrastive federated learning (MOON)^29^ performs contrastive learning based on positive pairs between the local and global models and on negative pairs between the current local model and the one from a previous round. Federated contrastive re-localization and history distillation (FedCRLD)^31^ enhances the positive correlation in MOON and the stability of local models via cross-attention and the local history distillation module, respectively. However, they are costly both in computational time and memory.

Recently, several works have presented solutions based on high-order information and managed to improve the generalization ability of the global model to a great extent. FedAlign^25^ distills the Lipschitz constant between the original network block and a slimmed one. FedASAM^26^ combines ASAM^35^ and Stochastic Weight Averaging (SWA)^42^ in FL, which is prolonged in training.

Existing methods in FL primarily focus on improving the optimization process, but they usually neglect the importance of data-related techniques due to the prohibition of data sharing among clients. Direct data augmentation may even lead to model degradation in FL, as proven in FedASAM.

### FPSS

Multi-encoding UNet (MENU-Net),^43^ as an early work in FPSS, trains a model with multiple encoders and deep supervision layers based on marginal and exclusive loss. However, some foreground channels for one client may be within a background one for other clients, which is also mentioned in a previous paper.^44^ Direct aggregation of the global segmentation head for all organs is bound to result in a class conflict problem, which is demonstrated in the empirical study of our work. Besides, the global model with multiple encoders^45^ is inferior to the one with multiple decoders.^46^ The same theory is verified in personalized FL (pFL).^47^

Naturally, a superior global model generalizes better than locally trained models for clients who may join the corporate training in the future, which is common in the FL setting. Therefore, our main purpose is to train a global model instead of personalized ones for FPSS. Compared with MENU-Net, we represent comprehensive results based on initially fully labeled datasets in our experiments. To our best knowledge, it is also the first time that the global model is trained by clients with partially annotated non-IID datasets, but the benchmark performance for all organs in each client is reported. Our method only requires a single forward pass during evaluation, regardless of the number of segmented organs. Moreover, the global model in our approach generalizes better than local models on unseen clients.

## Results

### Empirical study

**Figure 2:**
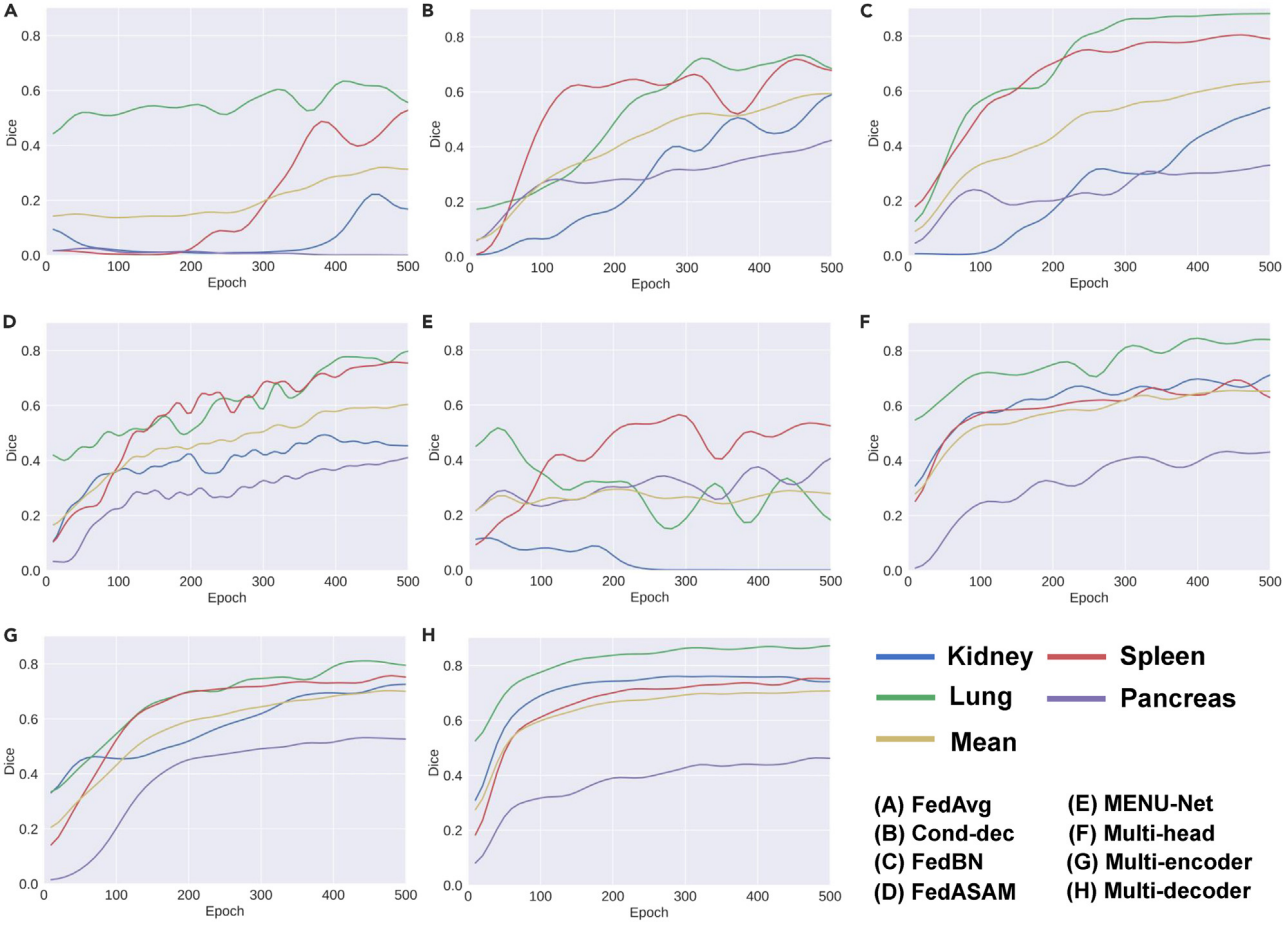
Mean test Dice curves over all clients for different combinations between PSS and FL

Before formulating FPSS, we first study the rationality of directly combining some methods for PSS with FL (Figure 1). Opt 1 in Figure 1 corresponds to Figure 2E, which is MENU-Net. Opt 2 in Figure 1 corresponds to Figures 2A–2D. Implementation details can be found in Note S6.

To investigate the effect of solutions in centralized learning on the class heterogeneity issue, we start from the simplest case, where locally trained segmentation networks are completely aggregated in each communication round. We observe that FedAvg (Figure 2A) fails to segment the pancreas in CT images, which is the hardest class among all organs due to its highly varied anatomical morphology across different domains. Besides, FedAvg suffers tremendous oscillation and slower convergence speed compared with the rest. We put the blame on the fact that foreground channels without supervision can be optimized to any false direction. Even with client index input into each layer of the decoder (Figure 2B) as auxiliary information, or with batch normalization layers separated for each client (Figure 2C) as feature decoupling to ease training, the severe oscillation still remains. When the method designed for the highly non-IID setting (Figure 2D) is used, successive fluctuations caused by class heterogeneity still exist. It proves that the class heterogeneity issue poses a more critical challenge than the client drift in FPSS.

When unannotated classes are merged into the background channel, MENU-Net (Figure 2E) suffers the most significant oscillation among all methods. This occurs because foreground annotations for each client are mistaken as background ones in other clients when there is no class intersections between clients. Consequently, it can be concluded that both options in Figure 1 are not suitable for FPSS because Opt 1 and Opt 2 cannot deal with class conflict for all classes and the background class, respectively.

As long as some certain part of the local model is separated from aggregation and the part is updated based on the loss for complete partial labels (e.g., one annotated class as foreground and its inverse set as background), the oscillation can be remarkably reduced. This phenomenon stresses the significance of simultaneously addressing class heterogeneity and client drift in FPSS. Furthermore, personalizing the decoder (Figure 2H) yields better performance than personalizing the encoder (Figure 2G). The reason for this observation is that global representations play a crucial role in enhancing the generalization ability of models in FL, which has also been proven by previous work in pFL.^46^ It is *a priori* that some classes are relevant in the medical field (e.g., relative organ position in CT images) and that layers around the bottleneck of the network usually extract high-level information. Hence, personalizing such parts may hinder potential interactions between classes during training. Despite the success of pFL-based methods, they suffer from long inference time, which is proportional to the number of classes.

Therefore, we aim to segment all classes without class conflict by filling up missing labels in a unified manner. Different from training personalized models, we also endeavor to train a generalized global model that only requires forwarding once during testing, no matter how many target classes there are. This model will absorb knowledge from all classes and all clients to learn about organ interactions.

### Problem formulation

In this subsection, we review objectives of PSS and FL and then define the formulation of FPSS based on empirical study.

We first give a short review of PSS. Let x∈X be the input and y∈Y be its corresponding annotated label map. Suppose the entire dataset $D^{p}={\left\{D_{i}^{p}\right\}}_{i=1}^{N}$ can be divided into *N* partially annotated datasets where each subset $D_{i}^{p}$ includes $N_{i}$ data samples; i.e., $D_{i}^{p}=\left(X_{i},Y_{i}^{p}\right)={\left\{{\left\{\left(x_{ij},y_{ij}^{p}\right)\right\}}_{j=1}^{N_{i}}\right\}}_{i=1}^{N}$. We denote $C_{i}\subset C$ as the label set of $Y_{i}$, where |C| is the total number of classes. Here, we conclude some properties about classes in PSS.

Property 1. The number of classes for the joint label space Y is fixed to $N_{c}$: $\left|\cup_{i=1}^{N}C_{i}\right|=N_{c}=\left|C\right|$.

Property 2. The amount of partially annotated classes for any subset $Y_{i}^{p}$ is usually limited: $0<\left|C_{i}\;\right|\ll N_{c}$.

Property 3. The intersection of classes is restricted: $\forall i,j\in \left[1,N\right],i\neq j,C_{i}\cap C_{j}=c_{i,j}$, where $0\leq \left|c_{i,j}\right|\ll N_{c}$.

Consider the FL setting with *N* clients and the overall dataset *D* with $N_{c}$ classes. Each client has the dataset $D_{i}$ separated from *D*. Let $w_{i},w_{0}$ denote the local model from client *i* and the global model, respectively. In each round, all clients upload their trained local model to the server for aggregation, and the server distributes the global model to clients as the initial local model at the next round. The global objective is to minimize the average of local empirical risks:

(Equation 1) $$\min_{w_{0}\in W}f\left(w_{0}\right)=\frac{1}{N}\sum_{i=1}^{N}f_{i}\left(D_{i},w_{0}\right)\text{,}$$

where f(·) is the loss function.

Now, we give the formulation of FPSS and list feasible solutions for it. Suppose each client has a partially labeled dataset $D_{i}^{p}$ from $D^{p}$. The global objective for FPSS is almost same as the one for FL (i.e., Equation 1) but with more constraints (i.e., three properties listed in the PSS setting):

(Equation 2) $$\min_{w_{0}\in W}f\left(w_{0}\right)=\frac{1}{N}\sum_{i=1}^{N}f_{i}\left(D_{i}^{p},w_{0}\right)\text{,}$$

Only with the modification to loss functions, it is unlikely to to achieve global minimal in Equation 2 based on partially-annotated datasets without any class intersection due to the class heterogeneity problem discussed before. Thus, one simple but feasible way is to separate part of the whole model from aggregation:

(Equation 3) $$\min_{w_{0}^{G}\in W^{G},w_{i}^{L}\in W^{L}}f\left(w_{0}^{G},w_{i}^{L}\right)=\frac{1}{N}\sum_{i=1}^{N}f_{i}\left(D_{i}^{p},w_{0}^{G},w_{i}^{L}\right)\text{.}$$

The whole model *w* can be divided into $w_{0}^{G},{\left\{w_{i}^{L}\right\}}_{i=1}^{L}$, denoting model parts aggregated globally and kept local, respectively.

### ULL

**Figure 3:**
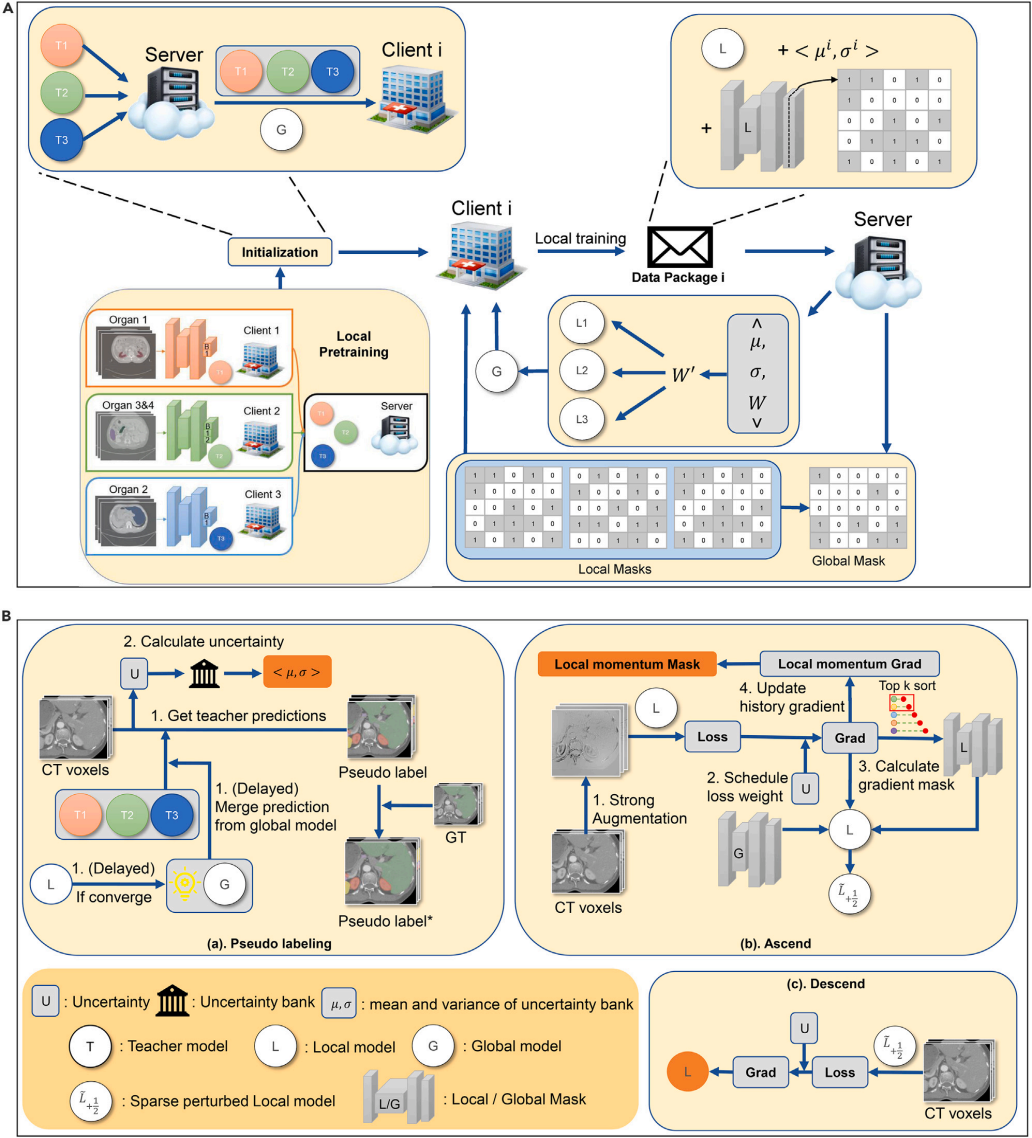
The overall flow of our proposed UFPS framework (A) FL process. Operations in pseudolabeling are condensed in the “Initialization” box. “Data Package” refers to the local mask, the mean and variance of uncertainty bank for client *i*, and the local model. The lower right part denotes taking the non-intersection part as the global mask. Aggregation weights are recomputed with statistics of the uncertainty bank and the original proportion weight. (B) Local training loop. The uncertainty score for each batch is deposited into the uncertainty bank and used to reweight loss. The local mask acquired in the ascent step is combined with part of the global mask to perturb the clean model. The local momentum mask is the mask sent to the server after local training. When local training converges, the global model replaces pretrained teacher models as the main teacher model.

In this subsection, we propose a pseudolabeling process for the class heterogeneity problem and mechanisms for the noisy label learning from both global and local perspectives. The overall flow is depicted in Figure 3.

#### Denoising pseudo label generation

A better solution to the class heterogeneity should be free from class confliction and improve the segmentation ability of the global model by learning class interactions. In ULL, each client *i* first pretrains a local model as a class-specific teacher model for other clients with partially annotated labels before federation. After sending the pretrained local teacher model and receiving pretrained teacher models $w^{T}$ from others, at each local round in FL, each client uses all pretrained teacher models to get pseudo-labels for all classes except ones with ground truth kept local. The background class for the pseudolabel is the intersection of background predictions from all teacher models, and foreground classes are merged in a predefined sequence. FL is then performed as

(Equation 4) $$\min_{\begin{array}{l} w_{0}\in W \end{array}}f\left(w_{0}\right)=\frac{1}{N}\sum_{i=1}^{N}f_{i}\left(X_{i},Y_{i,gt},PL_{i}\left(X_{i},w^{T}\right),w_{0}\right)\text{,}$$

where $PL_{i}$ represents the operation to predict pseudolabels for $X_{i}$ by $w^{T}$. $Y_{i,gt}$ denotes the annotated ground-truth labels within foreground classes for each client *i*. However, even though the annotated class is replaced by the ground truth, noise in rest channels may still be severe because the process of pseudo labeling is hindered by domain gaps. For this reason, the class heterogeneity problem is naturally transformed into a noisy label learning issue.

Serving as the direct source of pseudolabels, a noise-robust teacher model can play an important role in the noisy learning problem. Otherwise, local models for full-organ segmentation may overfit erroneous information in noisy pseudo-labels, thus being stuck at a local minimum. In a previous study,^48^ a global model trained on a labeled public dataset is used as the teacher model to provide pseudolabels because it is more reliable than local models. However, such a public dataset is not always available in the medical domain due to privacy concerns. Despite this restriction in our setting, predictions from the global model can still become less noisy than ones from local teacher models at some point in time since the global model absorbs knowledge from multiple organs and global data distribution. Therefore, we use the global model as the main teacher model (global main teacher [GMT]) during the training course at that time to better supervise local models.

Although the ability of the global model to locate organs is promoted, its accuracy in predicting segmentation boundaries may be reduced. This phenomenon can be explained by the fact that the global model in FL is usually smoother than locally trained models because client drifts result in counteractions in some dimensions. Therefore, we use locally pretrained models as auxiliary teacher models to refine boundary areas. Specifically, when the foreground prediction intersection of a patch between the global model and auxiliary teacher models is greater than the volume percentage threshold v, we use the intersection as the pseudolabel. Otherwise, we only use forecast from the global model as convincing pseudosupervision:

(Equation 5) $$\tilde{q}=\left\{ \begin{array}{l} {\tilde{q}}^{G},\left|{\tilde{q}}_{c\neq 0}^{w^{T}}\cap {\tilde{q}}_{c\neq 0}^{G}\right|<v\cdot \left|{\tilde{q}}_{c\neq 0}^{G}\right|,\\ {\tilde{q}}^{w^{T}}\cap {\tilde{q}}^{G},\text{else}, \end{array}\right.$$

where ${\tilde{q}}^{G}$ denotes the one-hot pseudolabel from the global model, and |·| here represents the volume of the prediction.

#### Enlarging impact of less noisy local models

The denoising effect of GMT counts on a reliable global model, which is directly affected by the noise degree of local models. To enhance reliability of the global model, we propose uncertainty-aware global aggregation (UA), which enlarges aggregation weights of less noisy local models.

It is common in FL that aggregation weights $A^{w}$ only depend on the number of samples. However, clients with a large amount of data do not necessarily have high-quality data. For FPSS, data quality can be reflected by confidence of pseudolabels. Using merged prediction from all teacher models, we first calculate data-wise uncertainty *U* for each sample *j*:

(Equation 6) $$U_{j}=\frac{1}{N_{c}}\sum_{c=0}^{N_{c}-1}\frac{\sum_{vox}E_{vox}\cdot {\tilde{q}}_{vox,c}^{w^{T}}}{\sum_{vox}{\tilde{q}}_{vox,c}^{w^{T}}+1}\text{,}$$

where $E_{vox}$ is average entropy across all classes, and vox denotes voxel. Uncertainty scores for each client are then deposited in their individual uncertainty bank.

Furthermore, because local teacher models are pretrained at different sites and for different organs, using only uncertainty of pseudolabels may not correctly rectify the aggregation weights. Thus, we calculate both mean $\mu_{i}$ and variance $\sigma_{i}$ of the uncertainty bank for each site and combine them with the number of samples to decide the aggregation weight of each client *i*:

(Equation 7) $${\hat{A}}_{i}^{w}=\frac{1}{3}\left(\frac{e^{-\frac{\mu_{i}}{\tau^{\mu }}}}{\sum_{j}^{N}e^{-\frac{\mu_{j}}{\tau^{\mu }}}}+\frac{e^{-\frac{\sigma_{i}}{\tau^{\sigma }}}}{\sum_{j}^{N}e^{-\frac{\sigma_{j}}{\tau^{\sigma }}}}+A_{i}^{w}\right)\text{,}$$

where $\tau^{\mu },\tau^{\sigma }$ are temperature hyperparameters for mean and variance, respectively. By assigning higher aggregation weights to these clients with reliable pseudolabels, the global model is less likely to be influenced by these label noises. Direct assignment of ${\hat{A}}_{i}^{w}$ can be improper because not all parts of the whole model are closely related to uncertainty. Consequently, we only apply this module to the decoder, which is most relevant to the final prediction.

#### Uncertainty-guided loss weight scheduler and noise robust loss

Another key factor restricting reliability of the global model is the inadequate learning for hard classes, leading to a higher level of noise compared with that of head classes. Pseudolabels with low confidence are more likely to be noisy or hard to segment. To avoid underfitting hard classes and overfitting pure noise, we propose to use weight scheduler (WS) based on self-entropy for loss functions. The proposed scheduler, named tail shift (TS), is formulated as

(Equation 8) $$\begin{array}{l} w\left(U_{j}\right)=\left\{ \begin{array}{l} 2-e^{\text{norm}\left(U_{j}\right)-\frac{r}{R}},U_{j}>U_{T},\\ 2-e^{\text{norm}\left(U_{j}\right)},\text{else}, \end{array}\right.\\ \text{where}\;\text{norm}\left(U_{j}\right)=\frac{U_{j}-\mu }{U_{\max}-U_{\min}}\text{,} \end{array}$$

where $U_{T}$ corresponds to the uncertainty value at lowest T percentage. $\mu ,U_{\max},U_{\min}$ represent mean, maximal, and minimal uncertainty in the uncertainty bank, respectively. $w\left(U_{j}\right)$ is then multiplied with the overall loss function to ensure enough fitting emphasis on hard classes. Other schedulers and their impact are introduced in Note S8.

Pseudo labels from teacher models can be quite noisy in some circumstances (e.g., restricted amount of labeled data).^49^^,^^50^ Because predictions from student models may become even more reliable than ones from teacher models during training, we use reverse cross-entropy (RCE)^51^ loss and reweight it based on the current training epoch *r* and total training epoch *R* (adaptive RCE loss [aRCE]):

(Equation 9) $$f_{aRCE}=e^{-20\left(1-\frac{r}{R}\right)}\cdot \left(q\left(x\right)\log\left(p\left(x\right)\right)\right)\text{,}$$

where q(x) is model prediction, and p(x) is ground truth.

### sUSAM

In this subsection, to alleviate the client drift problem, we introduce a unified ASAM (USAM) and its accelerating version, sUSAM, based on the ASAM framework.

#### Optimizing toward global direction with strong data augmentation

FedASAM has been proven as an effective method for the client drift problem. In the ascent step, the objective is to approximate the steepest optimization direction. By further optimizing from the sharpest direction based on the original parameter in the descent step, the global model achieves flatter minima and a smoother loss landscape at each iteration.

However, local models in FedASAM may overfit some local-specific attributes, thus failing to generalize on the non-IID global distribution. Therefore, we aim to find the steepest global direction in a unified manner while maintaining the modeling capacity of local datasets. Unlike previous methods dealing with data heterogeneity in FL, we alleviate the model drift issue by approximating the underlying global data distribution through data augmentation.

From theorem 1 in Note S2, it can be concluded that the gap between $D_{\text{global}\;}$ and $D_{\text{aug}\;}$ is mainly decided by constant *g* and its increment, caused by excessive data augmentation. Local models thus incur a larger error of the upper bound for generalization on $D_{\text{global}\;}$. Our key insights are twofold. First, local models can be free from performance degradation when optimized on strongly augmented datasets indirectly. Second, the global model generalizes for unseen clients better when the local data distribution is extended to the global one through comprehensive augmentations in a privacy-preserving manner.

Thus, we propose USAM to optimize local models toward the global direction. Because our setting for FPSS concentrates on medical images, we apply causality-inspired medical image domain generalization (CMIDG),^52^ which is designed for single-source domain medical image segmentation (e.g., CT and MRI). It is a causality-inspired data augmentation method, and we apply it to local datasets to imitate the underlying global data distribution. CMIDG integrates medical priors and simulates real-world data from various data centers by inflicting non-linear medical noises on data. Thus, we can naturally treat CMIDG as a strong and reasonable data augmentation method to explore global cliffy ways. When CMIDG is used for both steps in ASAM in our experiment, the global model is more unstable just as what is verified in theorem 1. Unlike FedASAM inputting the same data for ascent and descent steps, we perform the ascent step of ASAM on the CMIDG-augmented data.

Because the time complexity of USAM is about twice that of FedAvg in our experiment, we only use USAM when the global model converges in late communication rounds and find that it works almost as well when used for more rounds.

#### Accelerating and supplementing USAM with a gradient mask

Although USAM has its potential to mitigate the impact of data heterogeneity, some sharp directions found in the ascent step may be relevant to attributes that only exist in a single local dataset. Besides, the time complexity of USAM is high even when performed for limited rounds. We propose sUSAM focusing only on the most essential parts of perturbation to tackle both issues. Note that the principle of accelerating effect is discussed in a previous paper.^53^

To illustrate whether all gradients deserve perturbation, we show the relative difference ratio of gradients between the pseudolabel baseline and USAM:

(Equation 10) $$r=\log\left|\frac{\nabla f_{USAM}-\nabla f_{\text{base}\;}}{\nabla f_{\text{base}\;}}\right|\text{.}$$

**Figure 4:**
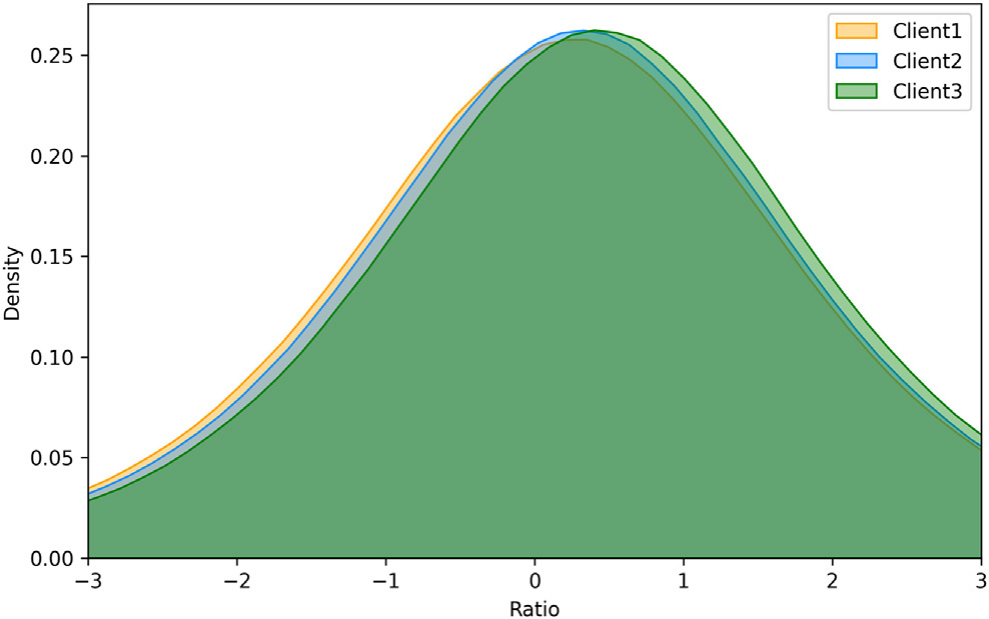
Relative difference ratio of gradients between the pseudolabel baseline and USAM

As demonstrated in Figure 4, about 60% of gradients are steep (ratio more than 0). Hence, we introduce a gradient mask $M_{L}$ to only retain gradients with the top *TL*% absolute values in the ascent step for USAM.

However, the sparse mask may remove some gradients accounting for vital global factors. These global factors may be of lesser importance to certain clients but highly emphasized by others. To bridge this semantic gap, we propose to replace a portion of local masks with the nonintersecting global mask. This approach involves three main steps: updating local masks and momentum gradients, communicating local and global masks, and merging them.

Local masks are always updated in the top-k manner. In the meantime, each client maintains local momentum gradients $G_{mo}$ in the ascent step, which is further used to calculate a momentum local mask $M_{L,mo}$. The local momentum gradients are updated at each iteration:

(Equation 11) $$G_{mo}=\alpha_{mo}G_{mo}+\left(1-\alpha_{mo}\right)\nabla f\text{,}$$

where $\alpha_{mo}$ is a hyperparameter empirically set to 0.9.

After completing local training, each client transmits $M_{L,mo}$ to the server, which represents the dominant positions of local features. When receiving momentum local masks from all clients, the server merges them as a global mask $M_{G}$, following the rule that nonintersecting parts of momentum local masks are assigned a value of 1, while the remaining areas are set to 0:

(Equation 12) $$\begin{array}{l} {\left(M_{G}\right)}_{0}={\left(\sum_{i=1}^{N}M_{L,mo}\right)}_{0}\cup {\left(\sum_{i=1}^{N}M_{L,mo}\right)}_{N}\text{,}\\ {\left(M_{G}\right)}_{1}=1-{\left(M_{G}\right)}_{0}\text{,} \end{array}$$

where ${\left(\cdot \right)}_{i}$ denotes positions with their values equal to *i*. The global mask ensures no redundant perturbation while exploring underlying global features in a unified manner. Compared with gradients of the float type, the global mask is of the bool type, so the extra communication burden and privacy leakage can be almost negligible.

For these gradients $G_{N}$ not in the top $T_{L}\%$ of $M_{L}$ but in the nonintersecting part of $M_{G}$, we randomly choose part of them to generate the extra perturbation mask $M_{E}$. Its total length is

(Equation 13) $$\left|M_{E}\right|=\min\left(T_{G}\left|\nabla f\right|,\left|G_{N}\right|\right)\text{,}$$

where $T_{G}$ is a hyperparameter to decide the proportion of the extra mask. The descent step at the *k*-th iteration based on sparse disturbance is formulated by the mergence of masks:

(Equation 14) $$w_{k+1}\leftarrow w_{k}-\nabla_{w_{k}}f\left(D,w_{k}\right){|}_{w_{k}+{\hat{\epsilon }}_{k}\cdot \left(M_{L}\cup M_{E}\right)}\text{.}$$

To further reduce computational costs and stabilize training, the update for all masks is conducted every $r_{fre}$ rounds. Otherwise, $G_{mo}$ is not accumulated, and a history local mask $M_{L}$ obtained from in the last update is used. In our experiment, the average computational cost of the local mask is only 5% of that for the local model, which can be considered negligible. Next, we provide a summary convergence analysis for both full and part participating scenarios. The detailed assumption, proof, and discussion can be found in Notes S3 and S4.

It can be concluded for theorem 2 and theorem 3 in Note S4 that the sparse ratio for masks has a direct impact on partial high-order terms. Because the mask in sUSAM constrains sparse gradients, the additional square and two-thirds terms are also negligible in magnitude. Furthermore, sUSAM has potential to generalize better by the dynamic mask, thus alleviating weight shifts in dominant terms for convergence.

## Experimental procedures

### Resource availability

#### Lead contact

Any further information, questions, or requests should be sent to Li Yan Ma (liyanma@shu.edu.cn).

#### Materials availability

Our study did not generate any physical materials.

#### Data and code availability

This study uses previously published datasets. Our source code is available at GitHub (https://github.com/tekap404/unified_federated_partially-labeled_segmentation) and has been archived at Zenodo.^54^

### Datasets

Main information of the datasets is listed in Table 1. We conduct our experiments with four fully annotated CT image datasets: whole abdominal organ dataset (WORD) (https://github.com/HiLab-git/WORD), abdominal CT organ segmentation dataset (AbdomenCT-1K, https://github.com/JunMa11/AbdomenCT-1K), abdominal organ segmentation dataset (AMOS, (https://amos22.grand-challenge.org) and multi-atlas labeling beyond the cranial vault (BTCV, https://www.synapse.org/#!Synapse:syn3193805/wiki/217752). Annotations for four organs are extracted from each dataset to serve as foreground classes: liver, kidneys (left and right), spleen, and pancreas. Whether a client is in the training process of FL is represented by “in-FL” and “out-FL.” Preprocessing details can be found in Note S6.

**Table 1:** Statistics of datasets

Dataset	WORD	AbdomenCT-1K	AMOS	BTCV
Total selected	120	266	200	30
Partial target	kidney	spleen & pancreas	liver	all
in-FL/out-FL	in-FL	in-FL	in-FL	out-FL
Client index	1	2	3	4

### Training

Only the partial target set and its inverse set (background) are used to pretrain class-specific teacher models. Default loss functions are Dice and binary cross entropy (BCE) losses. We train all methods for 500 communication rounds, with 1 local round for each global round. We conduct 10 warm-up rounds to increase the minimal learning rate and accumulate uncertainty values for loss WS. Unless otherwise specified, post-processing techniques (i.e., filling up holes and deleting small connected components) are not applied. We only show Dice and hausdorff distance (HD) for the mean of each dataset. Please refer to Note S6 for training details and complete results under more metrics.

### Main results

#### Comparison with SOTAs

**Figure 5:**
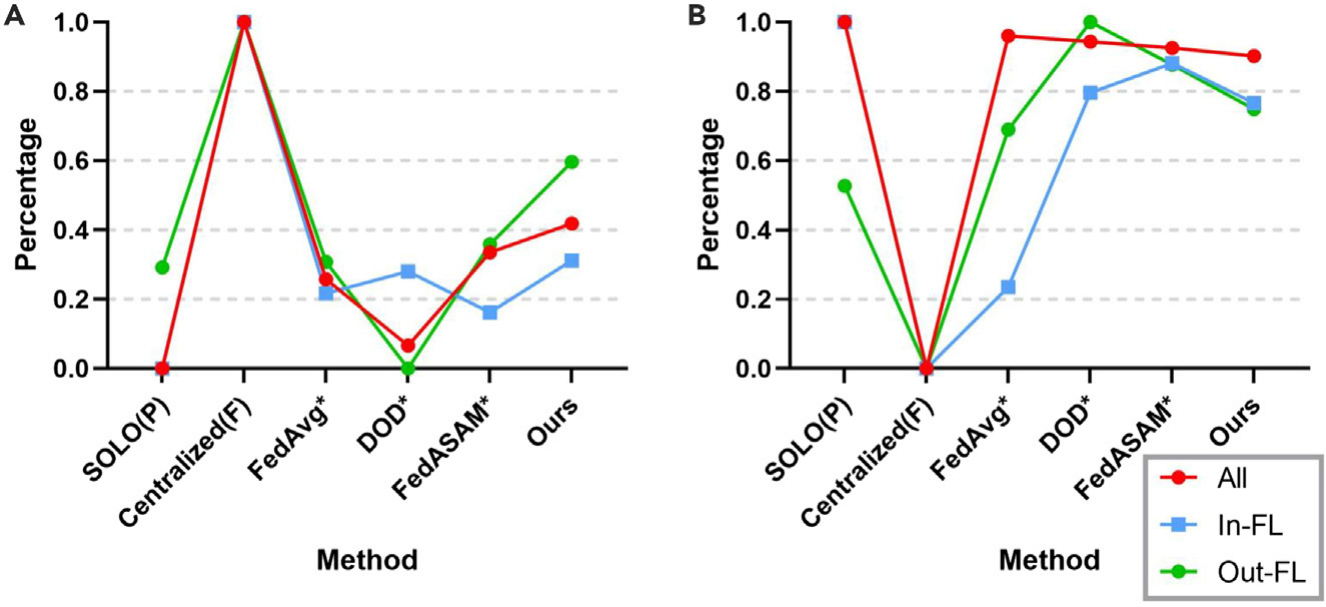
Client-wise comparison between SOTAs after post-processing (A) Normalized Dice (↑). (B) Normalized HD (↓).

**Figure 6:**
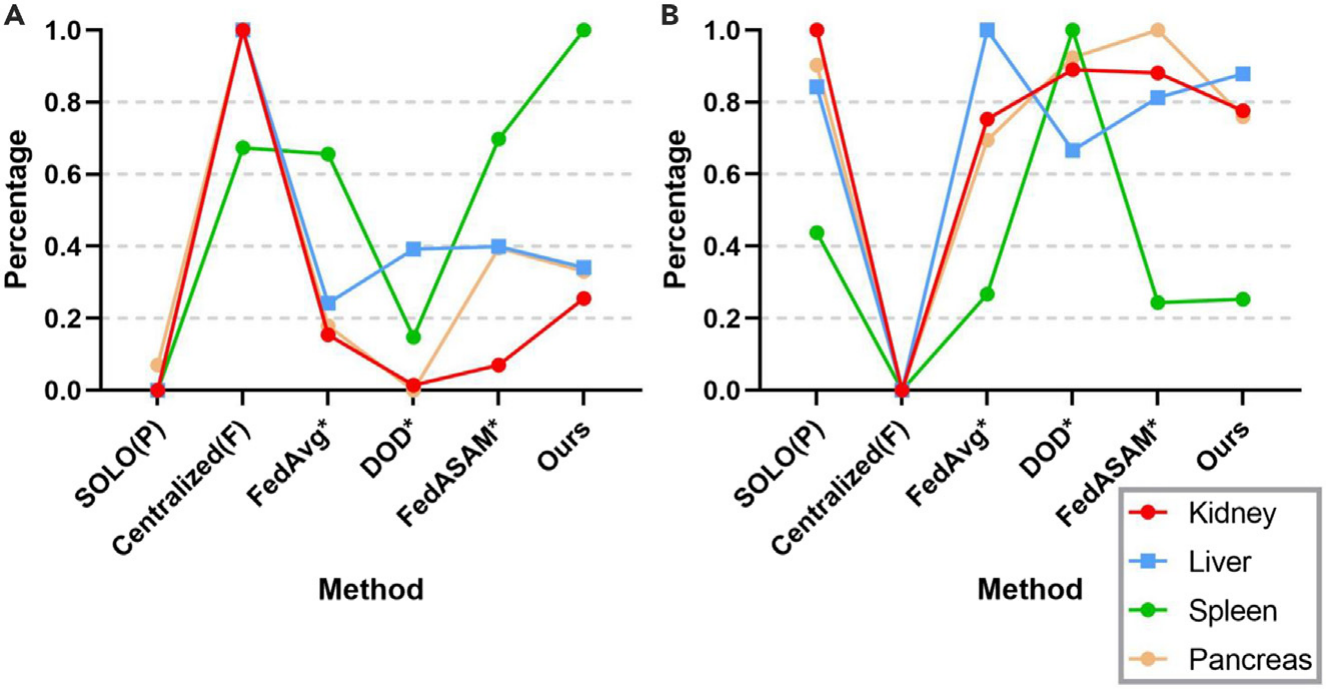
Organ-wise comparison between SOTAs after post-processing (A) Normalized Dice (↑). (B) Normalized HD (↓).

To demonstrate the lower and upper bounds of benchmarks, we first conduct experiments under local training (SOLO) and centralized training (centralized) based on partially annotated datasets and fully annotated ones, respectively. We use models pretrained in SOLO as organ-specific teacher models to generate pseudolabels. To showcase the effectiveness of UFPS, we compare UFPS with a variety of SOTA methods. FedAvg∗ is a combination of FedAvg and our proposed pseudolabeling procedure. Note that DOD (DoD-Net) is originally a partially annotated method based on part model aggregation for centralized learning. We modify it into the pFL setting. FedASAM is a method designed for heterogeneous data in fully annotated FL. Here, we incorporate FedASAM into the pseudolabeling framework for partially annotated segmentation as FedASAM∗. Details for all methods can be found in Note S6. Experimental results on in-FL and out-FL datasets for representative methods are shown in Table 2 and Figures 5 and 6.

**Table 2:** Comparison with SOTAs

Method	Client 1	Client 2	Client 3	Client 4	Mean	Post
SOLO (partial, lower bound)	69.17/1.02	75.75/3.02	60.59/1.61	74.70/1.52	70.05/1.79	69.93/1.66
Centralized (full, upper bound)	78.76/1.10	88.33/1.72	79.18/1.33	80.78/1.41	81.76/1.39	82.25/1.23
FedCRLD	67.82/2.21	77.77/3.06	58.11/2.06	72.30/1.84	69.00/2.29	68.14/1.76
DOD∗	61.95/1.15	81.76/2.59	60.57/1.69	53.52/1.14	70.62/1.72	70.77/1.63
CPS∗	75.78/0.99	78.05/2.91	65.75/1.70	75.60/1.65	73.80/1.81	73.78/1.62
MS-KD∗	74.35/1.00	76.68/2.96	63.33/1.66	73.10/1.64	71.86/1.81	71.77/1.82
FedAvg∗	74.94/1.29	78.10/2.83	64.53/1.83	74.74/1.69	73.07/1.91	73.13/1.64
FedProx∗	74.41/1.39	77.33/2.88	64.56/1.75	75.60/1.64	72.97/1.92	73.06/1.64
MOON∗	75.00/1.22	77.89/2.88	64.10/1.83	75.25/1.69	73.06/1.90	73.12/1.61
FedAlign∗	75.18/1.20	77.07/2.89	64.31/1.77	76.22/1.62	73.20/1.87	73.28/1.61
FedASAM∗	77.02/1.39	78.15/2.91	65.60/1.75	75.14/1.71	73.97/1.94	74.20/1.63
UFPS (ours)	76.22/1.45	79.56/2.82	66.82/2.04	77.22/1.72	74.95/2.01	75.28/1.62

“Post” represents mean results after post-processing. Here we only show Dice/HD (higher/lower numbers are better) for the mean of each dataset. All methods marked by an asterisk are not FPSS methods originally and modified to fit the FPSS setting. DOD∗ is combined with the multidecoder setting in the empirical study. Others with an asterisk are combined with the pseudolabeling procedure. Please refer to Note S6 for modification details and Note S7 for complete results.

The benchmark, SOLO (partial), is based on partially annotated datasets within each client, thus incurring a severe domain gap for client 1 and client 3. pFL-based approaches (i.e., DOD∗ and FedCRLD) are essentially not compatible with FPSS. This is because recognizing all classes heavily depends on global universal features, as also demonstrated previously.^55^ Furthermore, pFL models are likely to overfit to local biased distributions. Therefore, although DOD∗ performs well for client 2, owing to the largest aggregation weight based on the data amount, segmentation results for the rest are even worse than those of SOLO. The performance trend of FedCRLD is analogical to DOD∗, with only client 2 free from the severe collapse. The local momentum model even worsens the overfitting issue. FedAvg∗, FedASAM∗, and UFPS (ours) are all based on the pseudolabeling framework, outperforming DOD∗. By unifying the class label space in FPSS, all of these methods significantly benefit from class interactions, which proves the effectiveness of using pseudolabels in FPSS for the first time.

Among all methods originally designed for PSS (i.e., CPS∗, MS-KD∗, and DOD∗), CPS∗ achieves the best performance (73.80 in Dice). Through co-training, the noise degree is somehow alleviated from a local perspective. This result supports our basic idea that the class heterogeneity problem can be translated to a noisy label learning issue.

Although the overall performance of MOON∗ is similar to FedAvg∗, we notice that Dice for client 4 is enhanced by 0.51, but in-FL results are not satisfying. For the form of contrastive loss in Li et al.,^29^ it can be concluded that forcing the local model to align with the global one and to keep away from its history version has potential to learn generalizable features for unseen domains. However, the feature extraction ability for local information may decline. Different from these contrastive learning-based methods (FedCRLD and MOON∗), UFPS not only considers the global distribution, but it also takes into account some features that may be too hard to emphasize for some clients but are commonly stressed by others. As a result, UFPS leads to improvements for all clients compared with FedAvg∗.

The tendency and principle of FedProx∗ are analogous to MOON∗ but with better out-FL performance and worse in-FL one. FedAlign also involves second-order calculation like our method. However, there is a large gap between it and sUSAM used alone, manifesting the significance of aligning global distribution under the highly non-IID setting. Because UFPS is universal for model types and regularization methods, appropriate combinations are probably beneficial.

As can be seen in Figure 5, thanks to ULL to denoise pseudolabels and sUSAM to optimize toward the global direction, our method outperforms other methods (except upper-bound Centralized Full) for both in-FL clients and out-FL clients. Specifically, our method increases Dice by 5.35 for the baseline and 1.08 for FedASAM∗. Through simple post processing, based on the accurate segmentation location and the intersecting predictions from teacher models in GMT, HD of our method can also be reduced to a satisfying level. This indicates that the segmentation border of our method is more refined than that of many methods. In Figure 6, it can be seen that our method even surpasses Centralized Full in the “Spleen” class, which proves the strong generalization ability of our method and its potential to save labor for labeling full annotations. Additionally, our approach also gains a significant margin for the “Kidney” class compared with methods (excluding the upper bound) and achieves approximate performance for other organs.

#### Ablation study

In this subsection, we prove the validity of each module proposed in our paper and provide primary ablation studies for all of them.

##### Module validity

Table 3 shows module effectiveness for UFPS. We can conclude that the order of module importance is GMT > WS > sUSAM > UA > aRCE. Because teacher models pretrained at client 1 and client 3 are not generalized enough for the global distribution, which is dominated by the dataset from client 2, predictions from local models can be gradually less noisy than these pseudolabels. Therefore, using aRCE as an additional loss leads to reasonable performance improvement. When WS is employed to force these models to concentrate on hard classes, all classes can be simultaneously learned with fair emphasis. This module also enables the exploration of class interactions by local models, resulting in significant performance enhancement for all clients. UA is specifically designed for these clients with high-quality data and head class annotations. By correctly adjusting the aggregation weight, the model performance is increased. When all classes are denoised through previous modules, GMT is able to generate more reliable pseudolabels compared with locally pretrained teacher models. Due to the fact that the global model is indirectly trained on global distribution, the overall performance becomes even better. Benefitting from optimization toward the global steepest direction at each site and guidance for latent global directions from other clients, our framework with sUSAM is profitable for most clients, with few extra computational costs.

**Table 3:** Ablation study on module validity

PL	aRCE	WS	UA	GMT	sUSAM	Client 1	Client 2	Client 3	Client 4	Mean
						69.17/1.02	75.75/3.02	60.59/1.61	74.70/1.52	70.05/1.79
✓						74.94/1.29	78.10/2.83	64.53/1.83	74.74/1.69	73.07/1.91
✓	✓					75.53/1.23	77.71/2.87	65.37/1.78	74.75/1.71	73.34/1.89
✓		✓				75.98/1.58	78.24/2.86	66.33/1.74	76.35/1.65	74.22/1.96
✓			✓			76.65/1.39	78.05/2.92	64.81/1.89	75.19/1.72	73.67/1.98
✓				✓		77.77/1.18	77.58/2.85	65.64/1.96	76.09/1.73	74.27/1.93
✓					✓	77.06/1.38	78.23/2.89	65.53/1.89	75.34/1.74	74.04/1.98
✓	✓	✓				75.99/1.44	78.22/2.86	66.89/1.92	76.48/1.70	74.39/1.98
✓	✓	✓	✓			76.93/1.37	78.08/2.89	66.56/1.95	76.22/1.62	74.44/1.98
✓	✓	✓	✓	✓		76.12/1.51	78.83/2.86	67.30/1.95	77.07/1.70	74.83/2.00
✓	✓	✓	✓	✓	✓	76.22/1.45	79.56/2.82	66.82/2.04	77.22/1.72	74.95/2.01

PL, pseudolabel; aRCE, adaptive RCE loss; WS, weight scheduler; UA, uncertainty-aware global aggregation; GMT, global main teacher; sUSAM, sparse unified sharpness aware minimization. Here we only show Dice/HD (higher/lower numbers are better) for the mean of each dataset. Please refer to Note S8 for more results.

In terms of the relationship between modules, WS plays a crucial role in ensuring the training quality during the early training phase. It mainly interacts with UA because this stage involves accumulating uncertainty values to model a reliable uncertainty distribution for each client. WS also ensures that the noise degree of pseudo labels is not too excessive to affect other modules implicitly. When GMT is enabled, it has reciprocal effects with sUSAM and UA. It can be explained by the advantages of sUSAM and UA. sUSAM performs global alignment for the global model. UA rectifies the aggregation weights to guarantee that the global model is dominated by local models trained with high-quality data. In return, GMT offers more accurate pseudolabels for the two modules. Analogously, aRCE mitigates the influence of noisy labels as well, thus forming a virtuous cycle with GMT.

##### Ablation for ULL

**Figure 7:**
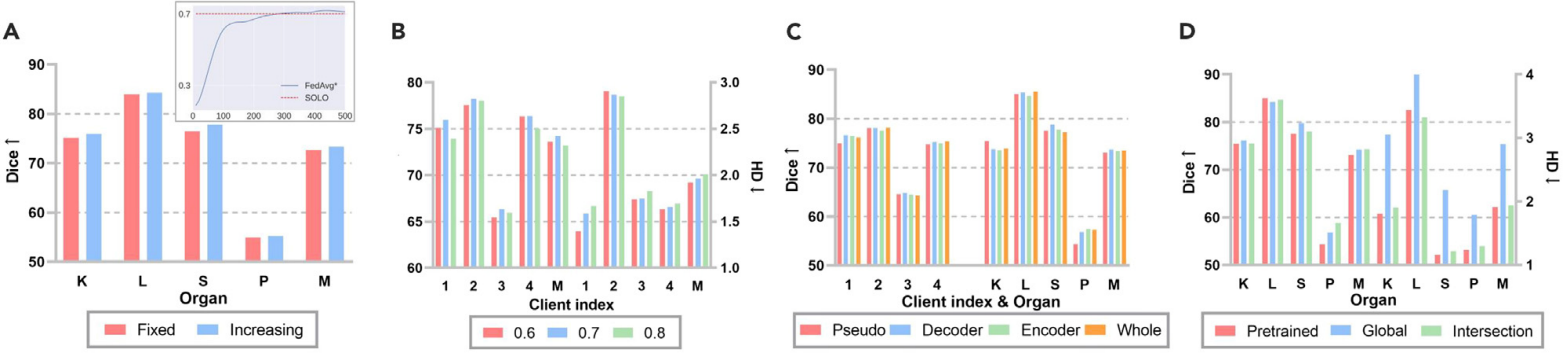
Ablation for ULL K, L, S, P, and M represent kidney, liver, spleen, pancreas, and mean, respectively. (A) Organ-wise Dice comparison for strategies of aRCE. The subfigure is a training Dice curve for FedAvg∗. (B) Client-wise Dice and HD comparison for the uncertainty threshold in WS. (C) Client-wise and organ-wise Dice comparison for module position of UA. (D) Organ-wise Dice and HD comparison for strategies of GMT.

Main ablations for ULL are shown in Figure 7. In Figure 7A, it can be observed that the model performance of FedAvg∗ gets higher during the training process and surpasses pretrained teacher models (i.e., SOLO) at the 300th epoch. Thus, it lays a foundation for the utilization of RCE loss because predictions are more accurate than pseudolabels due to organ interactions. The result in the larger picture further proves our assumption that increasing the coefficient for RCE loss, aRCE loss, is better than a fixed one for the increasing reliability of local models.

For the threshold of shifting in Figure 7B, when it is set to a moderate value, the local model is neither greatly affected by label noise nor easily neglects tail classes, thus enhancing performance for all clients. Due to the sensitivity of the threshold, in our future work, we intend to solve this problem by determining it adaptively.

As demonstrated in Figure 7C, in this experimental setting, client 1 only has the “Kidney” class annotated before FL, whose aggregation weight is increased most among all clients through UA. However, the promotion is not from the “Kidney” class. It proves that the specific class(es) with ground truth annotation is not necessarily closely related to the overall uncertainty of pseudolabels. Instead, the key factor lies in the global model aggregation based on the mean and variance of uncertainty. It adjusts model aggregation weights to prioritize clients with high-quality data but a smaller amount. This operation minimally sacrifices fitting ability for other clients compared with the pseudolabeling baseline. Furthermore, conducting this module merely on the decoder is slightly better than on the whole model but much better than on the encoder or on deep supervision layers. The reason for this phenomenon is probably due to the closer relationship between prediction uncertainty and the decoder.

In Figure 7D, it is evident that, when the global model is taken as the main teacher model after a certain point (i.e., the 300th epoch in our experiment), it achieves a huge performance gain for the client who has worse performance. This increment is due to its better generalization ability than that of pretrained teacher models. Furthermore, when we take the intersection between global and pretrained teacher models, regions with high confidence are selected as our final prediction. Therefore, the ambiguity of borders can be significantly alleviated, which is proven by HD.

##### Ablation for sUSAM

**Figure 8:**
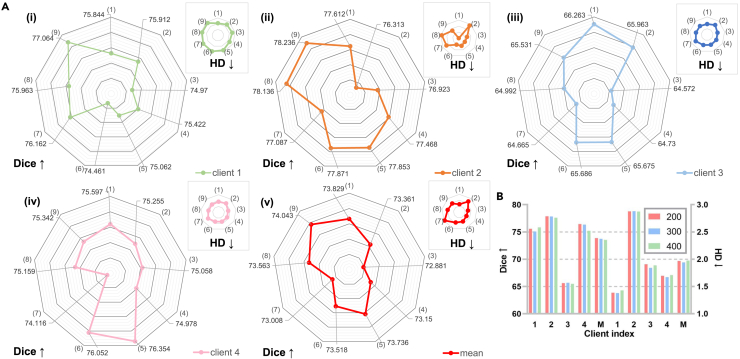
Strategy ablation for sUSAM (A) The larger radar map corresponds to Dice, and the smaller one corresponds to HD. (1) CMIDG from the beginning. (2) CMIDG from the 300th epoch. (3) Original data + ASAM. (4) Random perturbation + ASAM. (5) USAM. (6) USAM + (0.8 ∗ original weight + 0.2 ∗ perturbed weight for the descent step). (7) CMIDG for both ascent and descent steps. (8) USAM + top k perturbation. (9) sUSAM. (B) The start epoch of USAM.

We first display Figure 8A to comprehensively validate our motivations for sUSAM. We use CMIDG as the strong data augmentation method, whose distribution density is in direct proportion to the number of epochs. The comparison between (1) and (2) indicates its sensitivity to training rounds, thus resulting in heavy computational costs because the augmentation is generated from a network. Because the local sharpest direction is not necessarily the global steepest direction, simply performing the ascent step of ASAM on original data in (3) intensifies client drifts and is even worse than random perturbation in (4). USAM (i.e., [5]), with comparison with (2) and (7), demonstrates our insight that local models can be free from performance degradation when optimized on strongly augmented datasets indirectly. That is, conducting CMIDG in the ascent step improves the generalization ability of models through abundant data distribution, while descending on the original data keeps their fitting ability to the local distribution. What should be paid most attention to is that the out-FL client, BTCV, achieves the best result in this setting, which shows the great potential of USAM to generalize better on unseen data distributions. If original parameters and perturbed ones are combined to calculate the loss (i.e., [6]), then model performance slightly drops, proving the necessity of the perturbation in the ascent step.

For partial gradient perturbation in (8), it degrades the model performance slightly due to some missing key gradients, just as experimental results in sparse sharpness-aware minimization (SSAM).^53^ Through the non-intersection global mask in sUSAM, underlying steep directions for the global distribution are fully explored. Consequently, model performance for in-FL clients in (9) is significantly improved.

When modifying the start epoch of USAM from 300 to 200 in Figure 8B, we observe that the gain from data density is limited, so we choose 300 as the start epoch to striking a balance between training speed and accuracy. Besides, benefitting from the most essential perturbation directions from a global perspective, our method, merely with sUSAM for 200 epochs, surpasses the SOTA method FedASAM with 500 epochs for ASAM.

**Figure 9:**
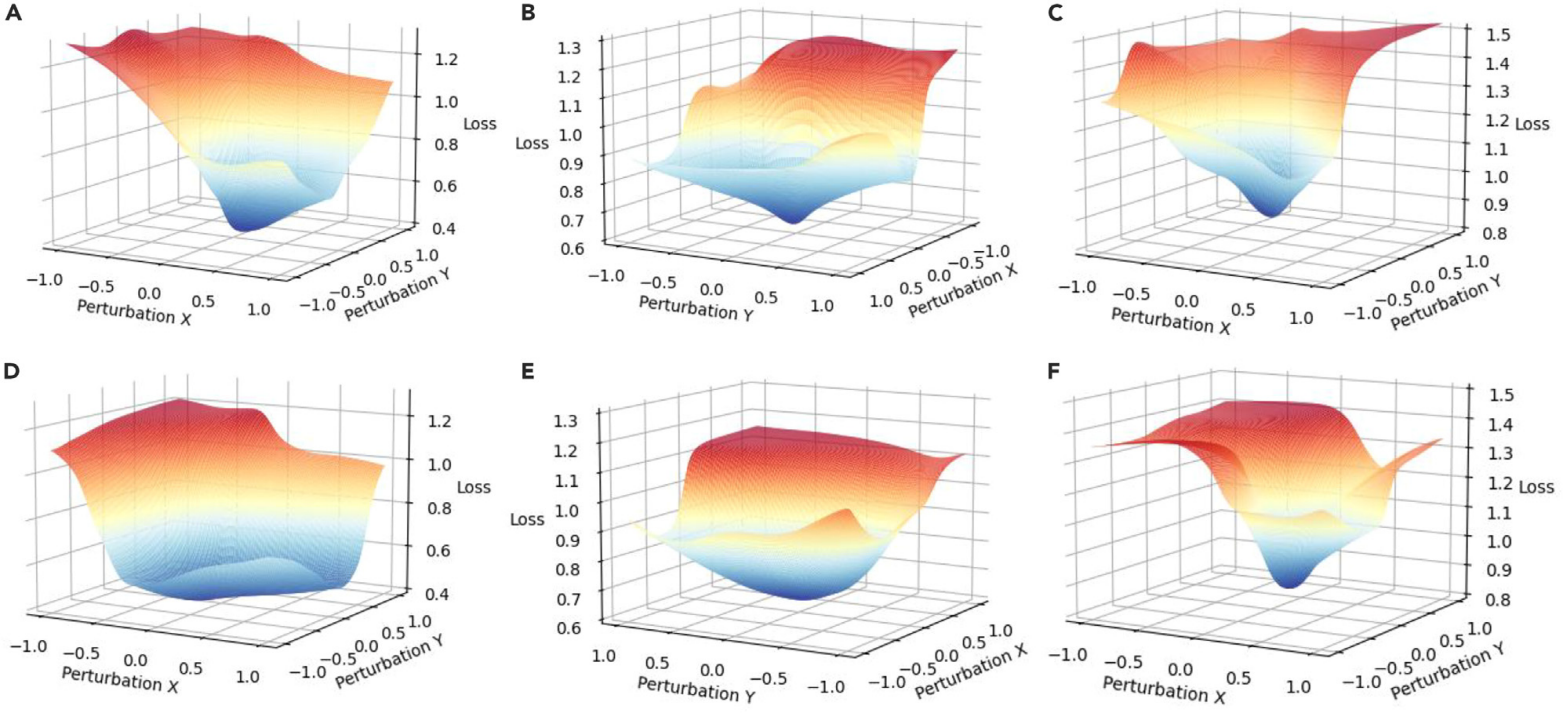
Loss landscape on the training set Model generalization is better when the overall loss landscape is flatter. (A–C) Loss landscapes from FedAvg∗ for client 1, client 2, and client 3, respectively. (D–F) Loss landscapes from UFPS (ours) for client 1, client 2, and client 3, respectively.

**Figure 10:**
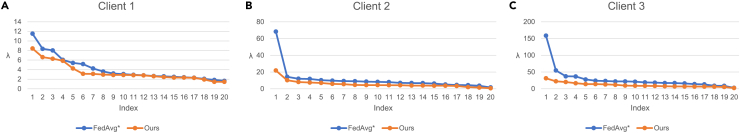
Hessian eigenspectra of the global model (A–C) Statistics of Hessian for client 1, client 2, and client 3, respectively.

To prove the generalization ability of sUSAM, we first plot the loss landscape on the training set in Figure 9. Compared with FedAvg∗ achieving sharp minima, UFPS achieves lower loss for all clients. For client 1, the landscape under a loss value of 0.8 is overall flatter. This is attributed to the corrected model aggregation weights and gradient mask for the global descending direction. All statistics extracted from the Hessian for the global model (Table 4; Figure 10) demonstrate that the generalization ability can be improved by seeking flatter minima explicitly in a heterogeneous setting.

**Table 4:** Statistics related to model generalization

Client	$\lambda_{\max}$ (pseudo)	$\lambda_{\max}$ (ours)	$\lambda_{\max}/\lambda_{5}$ (pseudo)	$\lambda_{\max}/\lambda_{5}$ (ours)	Trace (pseudo)	Trace (ours)
1	11.512	8.422	2.125	1.956	170.1	60.8
2	68.286	21.84	6.643	3.132	393.7	269.6
3	158.768	31.221	5.723	2.245	165.6	57.2

Model generalization is better when all of these statics are lower. $\lambda_{\max}$ and $\lambda_{5}$ mean the top eigenvalue and the fifth top eigenvalue of the Hessian for the global model, respectively.

#### Visual evaluation

**Figure 11:**
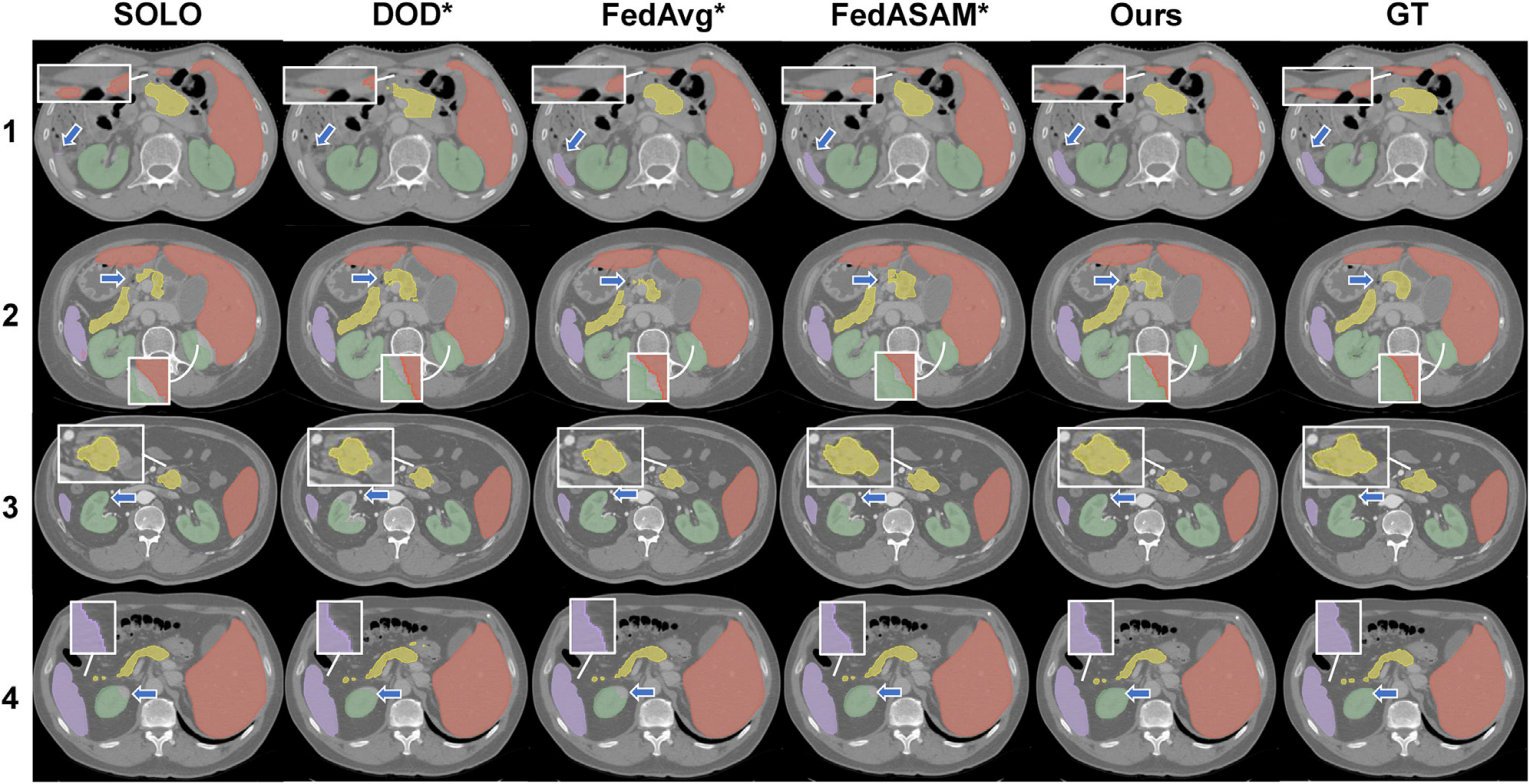
2D segmentation result on the test set Numbers on the left side of images refer to the client index. Green, red, purple, and yellow regions represent kidney, liver, spleen and pancreas, respectively.

Because the global model in our method is trained from multiple sites and organs, it is effective to reduce false negatives compared with other methods; e.g., spleen in client 1 and kidneys in clients 2 and 3 in Figure 11. Furthermore, the overall contour predicted by our global model is obviously smoother, especially for the pancreas and junctions between organs.

**Figure 12:**
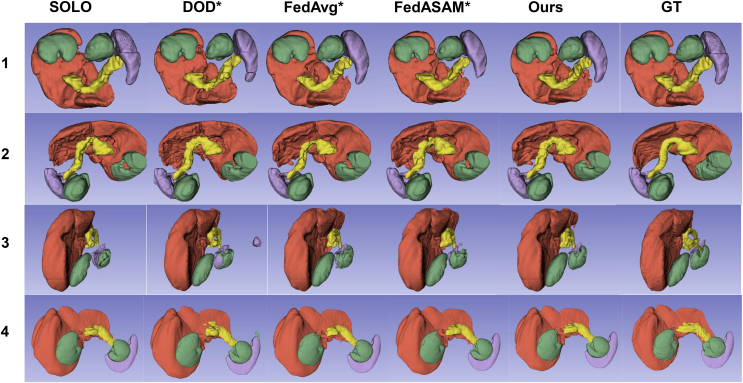
3D segmentation result on the test set Numbers on the left side of images refer to client index. Green, red, purple, and yellow regions represent kidney, liver, spleen, and pancreas, respectively.

In terms of the 3D segmentation results in Figure 12, DOD∗ generates more false positives for client 3 and client 4. It can explained by the facts that data distribution of client 3 is relatively biased from the global one and that client 4 is not involved in training. These findings highlight the limited generalization ability of personalized models. In contrast, UFPS uses a single model, which generalizes well on all datasets and classes. Additionally, UFPS is also able to correct some unnatural segmentations in ground truth, e.g., spleen in client 1, which shows the great potential of our method for real-world applications.

## Discussion

In this work, we analyze challenges about FPSS for direct combinations between PSS and FL methods. Our proposed framework, UFPS, is able to segment all classes based on several partially annotated datasets with a single global model. The training process of UFPS integrates ULL and sUSAM. While ULL denoises pseudolabels and explores underlying values in hard classes, sUSAM unifies the local training in FL to a global direction. The overall framework is computationally efficient and time saving during test time compared with pFL-based methods.

Our experiments demonstrate the strong generalization ability of UFPS because it incorporates knowledge from multiple sites and captures organ interactions. The effectiveness and sensitivity of hyperparameters for each module in ULL are also comprehensively investigated. Through detailed module ablation studies of sUSAM, we verify our key insights into how to enhance the ASAM-based framework for a more generalized and faster version in FL.

In terms of limitations, some hyperparameters (e.g., the threshold in WS and perturbation radius of UFPS) require fine-tuning. This issue can be resolved by reinforced learning or other automatic parameter-adjusting methods.

## Supplemental information

Document S1. Notes S1–S10, Figures S1–S5, and Tables S1–S13

Document S2. Article plus supplemental information
