# Supplementary material for: UFPS: A unified framework for partially annotated federated segmentation in heterogeneous data distribution
Source: Patterns (N Y). 2024 Jan 25;5(2):100917. doi: 10.1016/j.patter.2024.100917 (PMC10873159; doi:10.1016/j.patter.2024.100917)
Supplement: Document S1. Notes S1–S10, Figures S1–S5, and Tables S1–S13 [file mmc1.pdf]

Patterns, Volume *5*

Supplemental information

UFPS: A unified framework for partially annotated federated segmentation in heterogeneous data distribution

Le Jiang, Li Yan Ma, Tie Yong Zeng, and Shi Hui Ying

Supplementary Information

Supplementary Note 1 : Notations

Table S1. Description for notations occurred in this paper.

Notation	Description
$(x, y/y^p)$	Image and corresponding fully-annotated / partially-annotated label.
$(X, Y/Y^p)$	Joint space for image and fully-annotated / partially-annotated label.
D_i^p/D^p	Partially-annotated dataset for i-th client. / Global partially-annotated dataset.
N_i/N	Number of data samples in D_i^p/D^p.
C	Label set for all classes occurring in Y.
$\|C_i\|$	Number of classes in label space for i-th client.
N_c	Number of classes in Y.
$c_{i,j}$	Intersection between C_i and C_j.
R	Total number of communication round.
K	Total number of local rounds.
w_i^r/w_0^r	Local model for i-th client at communication round r. / Global model at communication round r.
$w^{G/L}$	Model part aggregated globally / kept local.
w_i^T/w^T	Teacher model pretrained by i-th client. / All pretrained teacher models.
$f(\cdot)$	Loss function.
PL	Operation to predict pseudo labels.
Y_l	Annotated ground-truth label within foreground classes.
$p(x)$	Ground truth.
$q(x)/\tilde{q}(x)$	Model prediction. / One-hot model prediction.
E	Information entropy.
$\mathcal{T}$	Hyper-parameter of uncertainty threshold.
$U/U_{\mathcal{T}}$	Data-wise uncertainty. / Uncertainty value at lowest $\mathcal{T}$ percentage.
U_{bank}	Uncertainty bank.
$\mu/\sigma/U_{\text{max / min}}$	Mean / variance / maximal / minimal uncertainty value for the uncertainty bank.
α	Hyper-parameter to determine the minimal base in BD.
β	Hyper-parameter to balance terms in RG.
$\tau^{\mu/\sigma}$	Temperature hyper-parameter for mean / variance in uncertainty-based aggregation.
A^w	Aggregation weight only based on the number of local samples.
υ	Hyper-parameter of volume threshold.
W	Joint model parameter space.
T_w^{-1}	Normalization operator.
η	Hyper-parameter to balance stability and universality in ASAM.
ρ	Hyper-parameter of searching radius for disturbance in ASAM.
$\nabla f_{\text{USAM}}/\nabla f_{\text{base}}$	Gradients of USAM / pseudo label baseline.
T_{sUSAM}	Hyper-parameter of local mask percentage threshold in sUSAM.
M_L/M_G	Local / Global mask in sUSAM.
T_L	Hyper-parameter of non-masking percentage of local mask in sUSAM.
T_G	Hyper-parameter of non-masking percentage of local mask according to the global mask in sUSAM.
$M_{L,mo}$	Momentum local mask.
α_{mo}	Hyper-parameter for the update of local momentum gradients.
G_N	Gradients not in top T_L% of M_L but in the nonintersecting part of M_G.
r_{warmup}	Number of warm-up rounds.
r_{WS}	End global round for weight scheduler.
$r_{UA/GMT/sUSAM}$	Start global round for uncertainty-based aggregation / global mean teacher / sUSAM.
r_{fre}	Hyper-parameter of the updating frequency for local mask in sUSAM.

Notations are described in Table S1.

Supplementary Note 2 : Proof of Theorem 1

Definition 1. *Given a bounded loss satisfying* $|f(x, w)| \leq f_{upper}$, *the Wasserstein distance between two data distribution* A, B *is:*

$$\mathbb{W}_{f_c}(A, B) = \inf_{f_{upper} \in \Pi(A,B)} \mathbb{E}_{f_{upper}} \left[f_c\left(X, X'\right) \right],$$

where $\Pi(A, B)$ *represents couplings of* A, B, f_c *denotes nonnegative, lower semi-continuous cost function.*

Here we cite the lemma from Sinha et.al [12], which demonstrates the result on any distribution A and B.

Lemma 2.1. *Let* $f : X \times W \to \mathbb{R}$ *and* $f_c : X \times X \to \mathbb{R}_+$ *be continuous. Let* $\varphi_\gamma\left(x', w\right) = \sup_{x \in X} \left[f(x, w) - \gamma c\left(x, x'\right) \right]$ *be the surrogate objective function. For any distribution* B *and any* $g > 0, \gamma > 0$

$$\sup_{\mathbb{W}_{f_c}(A,B) \leq g} \mathbb{E}_A[f(X, w)] = \inf_{\gamma \geq 0} \gamma g + \mathbb{E}_B \left[\varphi_\gamma\left(x', w\right) \right],$$

and for any $\gamma \geq 0$, *we have*

$$\sup_A \left\{ \mathbb{E}_A[f(X, w)] - \gamma \mathbb{W}_{f_c}(A, B) \right\} = \mathbb{E}_B \left[\varphi_\gamma(X, w) \right].$$

Theorem 1. *For any constant* $t > 0, g > 0, \gamma > 0, w \in W$, *expected error of risk function on the global data distribution satisfies the following inequality at the probability of* $1 - e^{-t}$*:*

$$\sup_{\mathbb{W}_{f_c}(D_{global}, D_{aug}) \leq g} \mathbb{E}_{D_{global}}[f(X, w)] \leq \mathbb{E}_{\widehat{D}_{aug}} \left[\varphi_\gamma(X, w) \right] + \gamma g + \epsilon_n(t),$$

$$\text{where } \epsilon_n(t) = \gamma b_1 \sqrt{\frac{f_{upper}}{n}} \cdot \int_0^1 \sqrt{\log N\left(\mathcal{F}, f_{upper}\,\epsilon, \|\cdot\|_{L^\infty(X)}\right)} d\epsilon + b_2 f_{upper} \sqrt{\frac{t}{n}},$$

D_{global}, D_{aug}, $\widehat{D}_{aug}$ *corresponds to global data distribution, augmented local data distribution and empiral augmented local data distribution, respectively,* $\varphi_\gamma(x, w) = \sup_{x \in X} \left[f\left(x', w\right) - \gamma f_c\left(x', x\right) \right]$ *is a surrogate objective function with penalty* γ *and augmentation* x' *of* x, n *is number of samples of* D_{aug}, $\mathcal{F}$ *is the hypothesis class,* $N(\cdot)$ *is covering numbers of* $\mathcal{F}$, b_1, b_2 *are both constants.*

Proof.

Leveraging Lemma 2.1 to our problem, for all $g > 0, \gamma > 0$, distribution D_{aug}, we have the deterministic result

$$\sup_{\mathbb{W}_{f_c}(D_{global}, D_{aug}) \leq g} \mathbb{E}_{D_{global}}[f(X, w)] \leq \mathbb{E}_{D_{\text{aug}}} \left[\varphi_\gamma(X, w) \right] + \gamma g.$$

To get the complete form of Theorem 1, we first show that for the empirical augmented local data distribution $\widehat{D}_{\text{aug}}$, $\mathbb{E}_{\widehat{D}_{\text{aug}}} \left[\varphi_\gamma(X, w) \right]$ concentrates around its population counterpart at the usual rate. Since $-f_{\text{upper}} \leq f(x, w) \leq \varphi_\gamma(X, w) \leq \sup_x f(x, w) \leq f_{\text{upper}}$, the functional $w \to F_n(w)$ satisfies bounded difference. Substituting standard result on Rademacher complexity [1] and entropy integrals [9] gives the result.

Supplementary Note 3 : Assumption

Assumption 1 (Smoothness)**.** $\forall i \in [N], w_a, w_b \in W, f_i$ *is* L*-smooth:*

$$\left\| \nabla f_i\left(w_a\right) - \nabla f_i\left(w_b\right) \right\| \leq L \left\| w_a - w_b \right\|.$$

Assumption 2 (Bounded variance of global gradient)**.** $\forall i \in [N], r \in [R]$, *the variance between global and local gradient for the global model is bounded by* σ_g *:*

$$\left\| \nabla f_i\left(w^r\right) - \nabla f\left(w^r\right) \right\|^2 \leq \sigma_g^2.$$

Assumption 3 (Bounded variance of stochastic gradient)**.** $\forall i \in [N]$, *the stochastic gradient* $\nabla f_i\left(w, \xi_i\right)$, *computed by the* i*-th client of* w *using mini-batch* ξ_i, *is an unbiased estimator* $\nabla f_i(w)$ *with variance bounded by* σ_l *:*

$$\mathbb{E}_{\xi_i} \left\| \frac{\nabla f_i\left(w, \xi_i\right)}{\left\| \nabla f_i\left(w, \xi_i\right) \right\|} - \frac{\nabla f_i(w)}{\left\| \nabla f_i(w) \right\|} \right\|^2 \leq \sigma_l^2,$$

where the expectation is over all local datasets.

Assumption 4 (Upper bound of stochastic gradient with sparse mask)**.** $\forall i \in [N]$*, the arbitrary stochastic gradient with sparse mask m is bounded by* G_s *:*

$$\mathbb{E}_{\xi_i} \left\| \frac{\nabla f_i\left(w, \xi_i\right)}{\left\|\nabla f_i\left(w, \xi_i\right)\right\|} \odot (1-m) \right\|^2 \leq G_s^2,$$

where G_s and m depend on hyper-parameters T_L, T_G.

Supplementary Note 4 : Proof of Theorem 2 and 3

Description of sUSAM Algorithm and Key Lemmas. We list flow of sUSAM in Algorithm 2. For the part client participating circumstance, we randomly choose $S^r \subseteq [N]$ clients with $|S^r| = S$ in global round r and conduct following updates: the server first sends the global model for local initialization $w^r_{i,0} = w^r$. Then each client updates the local model in K local rounds:

$$\widetilde{w}^r_{i,k+\frac{1}{2}} = w^r_{i,k-1} + \rho \frac{g^r_{i,k-1}}{\left\|g^r_{i,k-1}\right\|} \odot m,$$

$$w^r_{i,k} = w^r_{i,k-1} + \eta_l \tilde{g}^r_{i,k+\frac{1}{2}},$$

$$\Delta^r_i = w^r_{i,k} - w^r,$$

$$\Delta^r = \frac{1}{N} \sum_{i=1}^{N} \Delta^{r-1}_i,$$

$$w^{r+1} = w^r + \eta_g \Delta^r,$$

where $g^r_{i,k-1}$ is the gradient at the r-th global round and $(k-1)$-th local round for client i, the fractional part and tilde signal in $\tilde{g}^r_{i,k+\frac{1}{2}}$ denotes 'already perturbed' and 'sparsely perturbed', respectively. η_g means the global learning rate.

Lemma 4.1. *(Relaxed triangle inequality). Let* $\{v_1, \dots, v_n\}$ *be n vectors in* $\mathbb{R}^d$*. The following folds: (1)* $\forall a > 0$, $\left\|v_i + v_j\right\|^2 \leq (1+a)\left\|v_i\right\|^2 + \left(1 + \frac{1}{a}\right)\left\|v_j\right\|^2$*, and (2)* $\|\sum_{i=1}^n v_i\|^2 \leq n \sum_{i=1}^n \|v_i\|^2$.

Lemma 4.2. *For random variables* $x_1, \dots, x_n$*, the following holds:*

$$\mathbb{E}\left[\|x_1 + \cdots + x_n\|^2\right] \leq n\mathbb{E}\left[\|x_1\|^2 + \cdots + \|x_n\|^2\right].$$

Lemma 4.3. *For independent, mean 0 variables* $x_1, \dots, x_n$*, the following holds:*

$$\mathbb{E}\left[\|x_1 + \cdots + x_n\|^2\right] \leq \mathbb{E}\left[\|x_1\|^2 + \cdots + \|x_n\|^2\right].$$

Lemma 4.4. *(Separating mean and variance for sUSAM). The accumulated sparse gradient for the i-th client at the k-th local round and the r-th global round in sUSAM is bounded by:*

$$\mathbb{E}\left[\left\|\sum_{k=0}^{K-1} \tilde{g}^r_{i,k}\right\|^2\right] \leq K \sum_{k=0}^{K-1} \mathbb{E}\left[\left\|\tilde{g}^r_{i,k+\frac{1}{2}}\right\|^2\right] + \frac{KL^2\rho^2}{N}\left(\sigma_l^2 + G_s^2\right).$$

Proof.

$$\begin{aligned}
&\mathbb{E}\left[\left\|\sum_{k=0}^{K-1} \tilde{g}^r_{i,k+\frac{1}{2}}\right\|^2\right] \\
&\overset{(1)}{=} \mathbb{E}\left[\left\|\sum_{k=0}^{K-1} \tilde{g}^r_{i,k+\frac{1}{2}}\right\|^2\right] + \mathbb{E}\left[\left\|\sum_{k=0}^{K-1}\left(\tilde{g}^r_{i,k+\frac{1}{2}} - \nabla f\left(w^r_{i,k+\frac{1}{2}}\right)\right)\right\|^2\right]
\end{aligned}$$

$$
\begin{aligned}
&\overset{(2)}{\leq} \sum_{k=0}^{K-1} \mathbb{E}\left[\left\|\tilde{g}^{r}_{i,k+\frac{1}{2}}\right\|^{2}\right] + L^{2} \sum_{k=0}^{K-1} \mathbb{E}\left[\left\|\frac{1}{N}\sum_{i\in[N]}\left(w^{r}_{i,k+\frac{1}{2}} - \widehat{w}^{r}_{i,k+\frac{1}{2}} + \widetilde{w}^{r}_{i,k+\frac{1}{2}} - w^{r}_{i,k+\frac{1}{2}}\right)\right\|^{2}\right] \\
&\overset{(3)}{\leq} K\sum_{k=0}^{K-1} \mathbb{E}\left[\left\|\tilde{g}^{r}_{i,k+\frac{1}{2}}\right\|^{2}\right] + L^{2}K\mathbb{E}\left[\frac{1}{N^{2}}\cdot N\cdot 2\left(\left\|w^{r}_{i,k+\frac{1}{2}} - \widehat{w}^{r}_{i,k+\frac{1}{2}}\right\|^{2} + \left\|\widetilde{w}^{r}_{i,k+\frac{1}{2}} - w^{r}_{i,k+\frac{1}{2}}\right\|^{2}\right)\right] \\
&\overset{(4)}{\leq} \sum_{k=0}^{K-1} \mathbb{E}\left[\left\|\tilde{g}^{r}_{i,k+\frac{1}{2}}\right\|^{2}\right] + \frac{2L^{2}K}{N}\mathbb{E}\left[\left\|w^{r}_{i,k-1} + \delta^{r}_{i,k}\left(w^{r}_{i,k-1},\xi^{r}_{i,k-1}\right) - w^{r}_{i,k-1} + \delta^{r}_{i,k}\left(w^{r}_{i,k-1}\right)\right\|^{2} + \left\|\widetilde{w}^{r}_{i,k} - w^{r}_{i,k}\right\|^{2}\right] \\
&\overset{(5)}{\leq} K\sum_{k=0}^{K-1} \mathbb{E}\left[\left\|\tilde{g}^{r}_{i,k+\frac{1}{2}}\right\|^{2}\right] + \frac{2L^{2}K}{N}\mathbb{E}\left[\left\|\rho\frac{\nabla f_i\left(w^{r}_{i,k-1},\xi_i\right)}{\left\|\nabla f_i\left(w^{r}_{i,k-1},\xi_i\right)\right\|} - \rho\frac{\nabla f_i\left(w^{r}_{i,k-1}\right)}{\left\|\nabla f_i\left(w^{r}_{i,k-1}\right)\right\|}\right\|^{2} + \left\|\widetilde{w}^{r}_{i,k} - w^{r}_{i,k}\right\|^{2}\right] \\
&\overset{(6)}{\leq} K\sum_{k=0}^{K-1} \mathbb{E}\left[\left\|\tilde{g}^{r}_{i,k+\frac{1}{2}}\right\|^{2}\right] + \frac{2L^{2}K\rho^{2}\sigma_l^{2}}{N} + \frac{2L^{2}K}{N}\mathbb{E}\left[\left\|\rho\frac{\nabla f_i\left(w^{r}_{i,k-1},\xi_i\right)}{\left\|\nabla f_i\left(w^{r}_{i,k-1},\xi_i\right)\right\|}\odot(1-m)\right\|^{2}\right] \\
&\overset{(7)}{\leq} K\sum_{k=0}^{K-1} \mathbb{E}\left[\left\|\tilde{g}^{r}_{i,k+\frac{1}{2}}\right\|^{2}\right] + \frac{2L^{2}K\rho^{2}}{N}\left(\sigma_l^{2} + G_s^{2}\right),
\end{aligned}
$$

where $\widehat{w}^{r}_{i,k}$ denotes unbiased perturbed weight without mask, $w^{r}_{i,k}$ denotes perturbed weight without mask, $\widetilde{w}^{r}_{i,k}$ denotes perturbed weight with mask. (1) is from the assumption that the stochastic gradient $\nabla f_i\left(\widetilde{w},\xi_i\right)$ computed by the i-th client of w using mini-batch ξ_i is an unbiased estimator $\nabla f_i(w)$ with variance bounded by σ_l. (2) is from Assumption 1. (3) is from Lemma 4.1 with $a = 1$ and Lemma 4.2. (4) is from the definition of perturbed weight. (5) extends the formulation of perturbation. (6) is similar to (4) and (5). (7) is from Assumption 4.

Lemma 4.5. *(Bounded variance of global perturbed gradient). The variance of local and global gradients with sparse perturbation $\tilde{\delta}$ can be bounded as:*

$$
\left\|\nabla f_i\left(\widetilde{w}_{+\frac{1}{2}}\right) - \nabla f\left(\widetilde{w}_{+\frac{1}{2}}\right)\right\|^{2} \leq 3\sigma_g^{2} + 6L^{2}\rho^{2}.
$$

Proof.

$$
\begin{aligned}
&\left\|\nabla f_i\left(\widetilde{w}_{+\frac{1}{2}}\right) - \nabla f\left(\widetilde{w}_{+\frac{1}{2}}\right)\right\|^{2} \\
&\overset{(1)}{=} \left\|\nabla f_i\left(w+\tilde{\delta}_i\right) - \nabla f\left(w+\tilde{\delta}\right)\right\|^{2} \\
&= \left\|\left(\nabla f_i\left(w+\tilde{\delta}_i\right) - \nabla f_i(w)\right) + \left(\nabla f_i(w) - \nabla f(w)\right) + \left(\nabla f(w) - \nabla f(w+\tilde{\delta})\right)\right\|^{2} \\
&\overset{(2)}{\leq} 3\left\|\nabla f_i\left(w+\tilde{\delta}_i\right) - \nabla f_i(w)\right\|^{2} + 3\left\|\nabla f_i(w) - \nabla f(w)\right\|^{2} + 3\left\|\nabla f(w) - \nabla f(w+\tilde{\delta})\right\|^{2} \\
&\overset{(3)}{\leq} 3\sigma_g^{2} + 3L^{2}\left\|w + \rho\cdot\mathrm{sign}\left(\nabla f_i(w)\right)\frac{\nabla f_i(w)}{\left\|\nabla f_i(w)\right\|}\odot m - w\right\|^{2} + 3L^{2}\left\|w + \rho\cdot\mathrm{sign}\left(\nabla f(w)\right)\frac{\nabla f(w)}{\left\|\nabla f(w)\right\|}\odot m - w\right\|^{2} \\
&\overset{(4)}{\leq} 3\sigma_g^{2} + 6L^{2}\rho^{2}.
\end{aligned}
$$

(1) is from the definition of perturbed weight. (2) is from Lemma 4.2. (3) is from Assumption 1, Assumption 2 and formulation of perturbation. (4) is from the fact that $\left\|\frac{\nabla f(w)}{\|\nabla f(w)\|}\odot m\right\|^{2} < 1$.

Lemma 4.6. *(Bounded ε_δ of sUSAM). Suppose $\exists\alpha_{g_s}$, s.t. $\frac{\left\|f_i\left(\widetilde{w}_{i,k+\frac{1}{2}}\right)\odot m\right\|^{2}}{\|\nabla f_i(w_{i,0})\|^{2}} \leq \alpha_{g_s}$, and local learning rates satisfy $\eta_l \leq \frac{1}{4KL}$. Denote $\delta_{i,k} = \rho\frac{\nabla f_i(w_{i,k}\xi_i)}{\|\nabla f_i(w_{i,k},\xi_i)\|}$, $\tilde{\delta} = \rho\frac{\nabla f(w)}{\|\nabla f(w)\|}\odot m, \tilde{\delta}_{i,k} = \rho\frac{\nabla f_i(w_{i,k},\xi_i)}{\|\nabla f_i(w_{i,k},\xi_i)\|}\odot m$. Under Assumption 1, 2, 3, 4, the shift of perturbation of sUSAM can be bounded as follows:*

$$
\varepsilon_\delta = \frac{1}{N}\sum_i \mathbb{E}\left[\|\tilde{\delta}_{i,k} - \tilde{\delta}\|^{2}\right] \leq 2\rho^{2}K^{2}L^{2}\eta_l^{2}\alpha_{g_s}.
$$

Proof.

When the local learning rate is small, the gradient $\nabla f_i\left(w_{i,k},\xi_i\right)$ is small too. Based on the first order Hessian approximation, the optimal gradient is:

$$\begin{aligned}\nabla f_i\left(w_{i,k}\right) &= \nabla f_i\left(w_{i,k-1}-\eta_l\tilde{g}_{i,k+\frac{1}{2}}\right)\\ &= \nabla f_i\left(w_{i,k-1}\right)-H\eta_l\tilde{g}_{i,k+\frac{1}{2}}-O\left(\left\|\eta_l\tilde{g}_{i,k+\frac{1}{2}}\right\|^2\right),\end{aligned}$$

where H is the Hessian matrix at $w_{i,k-1}$. The shift of perturbation is then:

$$\begin{aligned}\varepsilon_\delta &= \mathbb{E}\left[\|\tilde{\delta}_{i,k}-\tilde{\delta}\|^2\right]\\ &= \rho^2\mathbb{E}\left[\left\|\left(\frac{\nabla f_i\left(w_{i,k}\right)}{\left\|\nabla f_i\left(w_{i,k}\right)\right\|}-\frac{\nabla f(w)}{\|\nabla f(w)\|}\right)\odot m\right\|^2\right]\\ &\overset{(1)}{\leq} \rho^2\frac{\left\|\left(\nabla f_i\left(w_{i,k}\right)-\nabla f_i\left(w_{i,0}\right)\right)\odot m\right\|^2}{\left\|\nabla f_i\left(w_{i,0}\right)\right\|^2}\\ &\overset{(2)}{\leq} \rho^2\left(\left(1+\frac{1}{k-1}\right)\cdot\frac{\left\|\left(\nabla f_i\left(w_{i,k-1}\right)-\nabla f_i\left(w_{i,0}\right)\right)\odot m\right\|^2}{\left\|\nabla f_i\left(w_{i,0}\right)\right\|^2}+K\frac{\left\|\left(H\eta_l\tilde{g}_{i,k+\frac{1}{2}}+\left(\left\|\eta_l\tilde{g}_{i,k+\frac{1}{2}}\right\|^2\right)\right)\odot m\right\|^2}{\left\|\nabla f_i\left(w_{i,0}\right)\right\|^2}\right)\\ &\overset{(3)}{\leq} \rho^2\left(\left(1+\frac{1}{k-1}\right)\cdot\frac{\left\|\left(\nabla f_i\left(w_{i,k-1}\right)-\nabla f_i\left(w_{i,0}\right)\right)\odot m\right\|^2}{\left\|\nabla f_i\left(w_{i,0}\right)\right\|^2}+KL^2\eta_l^2\alpha_{g_s}\right)\\ &\frac{1}{N}\sum_{i\in[N]}\mathbb{E}\left[\|\tilde{\delta}_{i,k}-\tilde{\delta}\|^2\right]\\ &\overset{(4)}{\leq} \rho^2\left(\sum_{\tau=0}^{k-1}\left(1+\frac{1}{k-1}\right)^\tau KL^2\eta_l^2\alpha_{g_s}\right)\\ &\leq 2\rho^2K^2L^2\eta_l^2\alpha_{g_s}.\end{aligned}$$

(1) can be explained through spherical coordinate system (Please refer to FedSAM[10] for more details). (2) is from the first order Hessian approximation and Lemma 4.1 with $a=\frac{1}{K-1}$. (3) is due to top eigenvalue of H is bounded by L under Assumption 1. Expanding the recursion between (1) and (3) results in (4).

Lemma 4.7. *(Bounded ε_w of sUSAM). Suppose local learning rates satisfy $\eta_l\leq\frac{1}{10KL}$. Under Assumption 1, 2, 3, 4, the shift of model parameters can of sUSAM can be bounded as follows:*

$$\varepsilon_w=\frac{1}{N}\sum_i\mathbb{E}\left[\|w_{i,k}-w\|^2\right]\leq 5K^2\eta_l^2\left(2L^2\rho^2\sigma_l^2+6K\left(3\sigma_g^2+6L^2\rho^2\right)+6K\|\nabla f(\widetilde{w})\|^2+24K^3{\eta_l}^4L^4\rho^2\right).$$

Proof of this Lemma is same as the one in FedSAM[10].

Lemma 4.8.

$$\begin{aligned}&\left\langle\nabla f\left(\widetilde{w}^r_{+\frac{1}{2}}\right),\mathbb{E}_r\left[\Delta^r\ +\eta_lK\nabla f\left(\widetilde{w}^r_{+\frac{1}{2}}\right)\right]\right\rangle\\ &\leq\frac{\eta_lK}{2}\left\|\nabla f\left(\widetilde{w}^r_{+\frac{1}{2}}\right)\right\|^2+K\eta_lL^2\varepsilon_w+K\eta_lL^2\varepsilon_\delta-\frac{\eta_l}{2KN^2}\mathbb{E}_r\left\|\sum_{i,k}\nabla f_i\left(\widetilde{w}^r_{i,k+\frac{1}{2}}\right)\right\|^2.\end{aligned}$$

Proof.

$$\left\langle\nabla f\left(\widetilde{w}^r_{+\frac{1}{2}}\right),\mathbb{E}_r\left[\Delta^r+\eta_lK\nabla f\left(\widetilde{w}^r_{+\frac{1}{2}}\right)\right]\right\rangle$$

$$\overset{(1)}{=} \sqrt{K}\frac{1}{\sqrt{K}}\left\langle \nabla f\left(\widetilde{w}^r_{+\frac{1}{2}}\right), \mathbb{E}_r\left[-\frac{\eta_l}{N}\sum_{i,k}\tilde{g}^{r-1}_{i,k-1} \ +\frac{\eta_l}{N}\sum_{i,k}\nabla f_i\left(\widetilde{w}^r_{+\frac{1}{2}}\right)\right]\right\rangle$$

$$\overset{(2)}{=} \frac{\eta_l K}{2}\left\|\nabla f\left(\widetilde{w}^r_{+\frac{1}{2}}\right)\right\|^2 + \frac{\eta_l}{2KN^2}\mathbb{E}_r\left\|\sum_{i,k}\left(\nabla f_i\left(\widetilde{w}^r_{i,k+\frac{1}{2}}\right) - \nabla f_i\left(\widetilde{w}^r_{+\frac{1}{2}}\right)\right)\right\|^2 - \frac{\eta_l}{2KN^2}\mathbb{E}_r\left\|\sum_{i,k}\nabla f_i\left(\widetilde{w}^r_{i,k+\frac{1}{2}}\right)\right\|^2$$

$$\overset{(3)}{\le} \frac{\eta_l K}{2}\left\|\nabla f\left(\widetilde{w}^r_{+\frac{1}{2}}\right)\right\|^2 + \frac{\eta_l}{2N}\sum_{i,k}\mathbb{E}_r\left\|\nabla f_i\left(\widetilde{w}^r_{i,k+\frac{1}{2}}\right) - \nabla f_i\left(\widetilde{w}^r_{+\frac{1}{2}}\right)\right\|^2 - \frac{\eta_l}{2KN^2}\mathbb{E}_r\left\|\sum_{i,k}\nabla f_i\left(\widetilde{w}^r_{i,k+\frac{1}{2}}\right)\right\|^2$$

$$\overset{(4)}{\le} \frac{\eta_l K}{2}\left\|\nabla f\left(\widetilde{w}^r_{+\frac{1}{2}}\right)\right\|^2 + \frac{\eta_l L^2}{2N}\sum_{i,k}\mathbb{E}_r\left\|\widetilde{w}^r_{i,k+\frac{1}{2}} - \widetilde{w}^r_{+\frac{1}{2}}\right\|^2 - \frac{\eta_l}{2KN^2}\mathbb{E}_r\left\|\sum_{i,k}\nabla f_i\left(\widetilde{w}^r_{i,k+\frac{1}{2}}\right)\right\|^2$$

$$\overset{(5)}{\le} \frac{\eta_l K}{2}\left\|\nabla f\left(\widetilde{w}^r_{+\frac{1}{2}}\right)\right\|^2 + \frac{\eta_l L^2}{N}\sum_{i,k}\mathbb{E}_r\left\|w^r_{i,k} - w^r\right\|^2 + \frac{\eta_l L^2}{N}\sum_{i,k}\mathbb{E}_r\left\|\tilde{\delta}^r_{i,k} - \tilde{\delta}^r\right\|^2 - \frac{\eta_l}{2KN^2}\mathbb{E}_r\left\|\sum_{i,k}\nabla f_i\left(\widetilde{w}^r_{i,k+\frac{1}{2}}\right)\right\|^2$$

$$\overset{(6)}{\le} \frac{\eta_l K}{2}\left\|\nabla f\left(\widetilde{w}^r_{+\frac{1}{2}}\right)\right\|^2 + K\eta_l L^2\varepsilon_w + K\eta_l L^2\varepsilon_\delta - \frac{\eta_l}{2KN^2}\mathbb{E}_r\left\|\sum_{i,k}\nabla f_i\left(\widetilde{w}^r_{i,k+\frac{1}{2}}\right)\right\|^2.$$

(1) can be derived from:

$$\begin{aligned}
&\eta_l K\nabla f\left(\widetilde{w}^r_{+\frac{1}{2}}\right)\\
&= \eta_l K\frac{1}{N}\sum_{i\in[N]}\nabla f_i\left(\widetilde{w}^r_{+\frac{1}{2}}\right)\\
&= \eta_l K\frac{1}{N}\sum_{i\in[N]}\frac{1}{K}\sum_{k\in[K]}\nabla f_i\left(\widetilde{w}^r_{+\frac{1}{2}}\right)\\
&= \frac{\eta_l}{N}\sum_{i,k}\nabla f_i\left(\widetilde{w}^r_{+\frac{1}{2}}\right),
\end{aligned}$$

$$\begin{aligned}
\Delta^r &= \frac{1}{N}\sum_{i=1}^{N}\Delta_i^{r-1}\\
&= \frac{1}{N}\sum_{i=1}^{N}\left(w^{r-1}_{i,0} - \sum_k \eta_l\tilde{g}^{r-1}_{i,k-1} - w^{r-1}_i\right)\\
&= -\frac{\eta_l}{N}\sum_{i,k}\tilde{g}^{r-1}_{i,k-1}.
\end{aligned}$$

(2) is from the lemma that $\langle a,b\rangle = \frac{1}{2}\left(\|a\|^2 + \|b\|^2 - \|a-b\|^2\right)$ with $a = \sqrt{\eta_l K}\nabla f\left(\widetilde{w}^r_{+\frac{1}{2}}\right)$ and $b = -\frac{\sqrt{\eta_l}}{N\sqrt{K}}\sum_{i,k}\left(\nabla f_i\left(\widetilde{w}^r_{i,k+\frac{1}{2}}\right) - \nabla f_i\left(\widetilde{w}^r_{+\frac{1}{2}}\right)\right)$. (3) is from Lemma 4.2. (4) is from Assumption 1. (5) is from definition of sparse perturbation and Lemma 4.2. (6) is from Lemma 4.6 and Lemma 4.7.

Lemma 4.9. *For the full client participating scheme, the bound of* $\mathbb{E}_r\left[\|\Delta^r\|^2\right]$ *is:*

$$\mathbb{E}_r\left[\|\Delta^r\|^2\right] \le \frac{2K\eta_l^2L^2\rho^2}{N}\left(\sigma_l^2 + G_s^2\right) + \frac{\eta_l^2}{N^2}\left\|\sum_{i,k}\nabla f_i\left(\widetilde{w}^r_{i,k+\frac{1}{2}}\right)\right\|^2.$$

Proof.

$$\begin{aligned}
&\mathbb{E}_r\left[\|\Delta^r\|^2\right]\\
&\overset{(1)}{\le} \frac{\eta_l^2}{N^2}\mathbb{E}_r\left\|\sum_{i,k}\tilde{g}^r_{i,k+\frac{1}{2}}\right\|^2
\end{aligned}$$

$$\overset{(2)}{=} \frac{\eta_l^2}{N^2}\mathbb{E}_r \left\| \sum_{i,k} \left(\tilde{g}^r_{i,k+\frac{1}{2}} - \nabla f_i \left(\tilde{w}^r_{i,k+\frac{1}{2}} \right) \right) \right\|^2 + \frac{\eta_l^2}{N^2}\mathbb{E}_r \left\| \sum_{i,k} \nabla f_i \left(\tilde{w}^r_{i,k+\frac{1}{2}} \right) \right\|^2$$

$$\overset{(3)}{\leq} \frac{2K\eta_l^2 L^2 \rho^2}{N} \left(\sigma_l^2 + G_s^2 \right) + \frac{\eta_l^2}{N^2}\mathbb{E}_r \left\| \sum_{i,k} \nabla f_i \left(\tilde{w}^r_{i,k+\frac{1}{2}} \right) \right\|^2 .$$

(1) is from Lemma 4.2 and the fact that the optimal gradient is smaller than the empiral one. (2) is from Lemma 4.3 since the gradient is merely within one local epoch k and the mean for r is 0 in this situation. (3) is scaled from the second term in Lemma 4.4.(1).

Lemma 4.10. *For all $r \in [R-1]$, with proper choices of local and global learning rates, the iterates from sUSAM satisfy:*

$$\mathbb{E}_r \left[f\left(\tilde{w}^{r+1}_{+\frac{1}{2}} \right) \right] \leq f\left(\tilde{w}^r_{+\frac{1}{2}} \right) - K\eta_g\eta_L \left(\frac{1}{2} - 30K^2L^2\eta_L^2 \right) \left\| \nabla f\left(\tilde{w}^r_{+\frac{1}{2}} \right) \right\|^2 + K\eta_g\eta_L \left(10KL^4\eta_L^2\rho^2\sigma_l^2 \right.$$
$$\left. + 90K^2L^2\eta_L^2\sigma_g^2 + 180K^2L^4\eta_L^2\rho^2 + 24K^4L^6\eta_L^6\rho^2\alpha_{g_s} + \frac{\eta_g\eta_L L^3\rho^2}{N} \left(\sigma_l^2 + G_s^2 \right) \right).$$

Proof.

$$\mathbb{E}_r \left[f\left(\tilde{w}^{r+1}_{+\frac{1}{2}} \right) \right]$$

$$\overset{(1)}{\leq} f\left(\tilde{w}^r_{+\frac{1}{2}} \right) + \mathbb{E}_r \left\langle \nabla f\left(\tilde{w}^r_{+\frac{1}{2}} \right), \tilde{w}^{r+1}_{+\frac{1}{2}} - \tilde{w}^r_{+\frac{1}{2}} \right\rangle + \frac{L}{2}\mathbb{E}_r \left\| \tilde{w}^{r+1}_{+\frac{1}{2}} - \tilde{w}^r_{+\frac{1}{2}} \right\|^2$$

$$\overset{(2)}{=} f\left(\tilde{w}^r_{+\frac{1}{2}} \right) + \mathbb{E}_r \left\langle \nabla f\left(\tilde{w}^r_{+\frac{1}{2}} \right), -\eta_g\Delta^r + K\eta_g\eta_L\nabla f\left(\tilde{w}^r_{+\frac{1}{2}} \right) - K\eta_g\eta_L\nabla f\left(\tilde{w}^r_{+\frac{1}{2}} \right) \right\rangle + \frac{L\eta_g^2}{2}\mathbb{E}_r \|\Delta^r\|^2$$

$$\overset{(3)}{=} f\left(\tilde{w}^r_{+\frac{1}{2}} \right) - K\eta_g\eta_L \left\| \nabla f\left(\tilde{w}^r_{+\frac{1}{2}} \right) \right\|^2 + \eta_g \left\langle \nabla f\left(\tilde{w}^r_{+\frac{1}{2}} \right), \mathbb{E}_r \left[-\Delta^r + K\eta_L\nabla f\left(\tilde{w}^r_{+\frac{1}{2}} \right) \right] \right\rangle$$
$$+ \frac{L\eta_g^2}{2}\mathbb{E}_r \|\Delta^r\|^2$$

$$\overset{(4)}{\leq} f\left(\tilde{w}^r_{+\frac{1}{2}} \right) - \frac{K\eta_g\eta_L}{2} \left\| \nabla f\left(\tilde{w}^r_{+\frac{1}{2}} \right) \right\|^2 + K\eta_g\eta_L L^2\varepsilon_w + K\eta_g\eta_L L^2\varepsilon_\delta - \frac{\eta_g\eta_L}{2KN}\mathbb{E}_r \left\| \sum_{i,k} \nabla f_i \left(\tilde{w}^r_{i,k+\frac{1}{2}} \right) \right\|^2$$
$$+ \frac{L}{2}\eta_g^2\mathbb{E}_r \|\Delta^r\|^2$$

$$\overset{(5)}{\leq} f\left(\tilde{w}^r_{+\frac{1}{2}} \right) - \frac{K\eta_g\eta_L}{2} \left\| \nabla f\left(\tilde{w}^r_{+\frac{1}{2}} \right) \right\|^2 + K\eta_g\eta_L L^2\varepsilon_w + K\eta_g\eta_L L^2\varepsilon_\delta + \frac{K\eta_g^2\eta_l^2 L^3\rho^2}{N} \left(\sigma_l^2 + G_s^2 \right)$$

$$\overset{(6)}{\leq} f\left(\tilde{w}^r_{+\frac{1}{2}} \right) - K\eta_g\eta_L \left(\frac{1}{2} - 30K^2L^2\eta_L^2 \right) \left\| \nabla f\left(\tilde{w}^r_{+\frac{1}{2}} \right) \right\|^2 + K\eta_g\eta_L \left(10KL^4\eta_L^2\rho^2\sigma_l^2 + 90K^2L^2\eta_L^2\sigma_g^2 \right.$$
$$\left. + 180K^2L^4\eta_L^2\rho^2 + 24K^4L^6\eta_L^6\rho^2\alpha_{g_s} + \frac{\eta_g\eta_L L^3\rho^2}{N} \left(\sigma_l^2 + G_s^2 \right) \right).$$

(1) is from the lemma of L-smooth that $f(a) \leq f(b) + \nabla f(b)(a-b) + \frac{L}{2}\|a-b\|^2$. (2) is from the description of sUSAM. (3) is from the unbiased estimators. (4) is from Lemma 4.3. (5) is from Lemma 4.9 and neglection of the negative term. (6) is from Lemma 4.6, Lemma 4.7 and the assumption that $\eta_l \leq \frac{1}{10KL}$.

Theorem 2. *Let local and global learning rates be set as $\eta_l \leq \frac{1}{10KL}, \eta_l\eta_G \leq \frac{1}{KL}$. Under Assumption 1, 2, 3, 4 and full client participation, the sequence of iterates generated by sUSAM satisfies:*

$$\min_{r\in[R]} \mathbb{E}\left[\|\nabla f(w^r)\|^2 \right] \leq \frac{f^0 - f^*}{CK\eta_g\eta_L} + \Phi ,$$

where $\Phi = \frac{1}{C}\left(10KL^4\eta_L^2\rho^2\sigma_l^2 + 90K^2L^2\eta_L^2\sigma_g^2 + 180K^2L^4\eta_L^2\rho^2 + 24K^4L^6\eta_L^6\rho^2\alpha_{g_s} + 16K^3L^6\eta_L^4\rho^2\alpha_{g_s} + \frac{\eta_g\eta_L L^3\rho^2}{N}\left(\sigma_l^2 + G_s^2\right)\right)$. *If local and global learning rates are chosen as* $\eta_l = \frac{1}{\sqrt{RKL}}$ *and* $\eta_G = \sqrt{KN}$, *and perturbation radius is chosen as* $\rho = \frac{1}{\sqrt{R}}$, *the convergence rates can be expressed as:*

$$\frac{1}{R}\sum_{r=1}^{R}\mathbb{E}\left[\left\|f\left(w^{r+1}\right)\right\|^2\right] = O\left(\frac{FL}{\sqrt{RKN}} + \frac{\sigma_g^2}{R} + \frac{L^2\left(\sigma_l^2 + G_s^2\right)}{R^{\frac{3}{2}}\sqrt{KN}} + \frac{L^2}{R^2} + \frac{L^2\sigma_l^2}{R^2K}\right).$$

Proof.

$$\begin{aligned}
&\frac{1}{R}\sum_{r=1}^{R}\mathbb{E}\left[\left\|f\left(w^{r+1}\right)\right\|^2\right] \\
&\overset{(1)}{=} \frac{1}{R}\sum_{r=1}^{R}\mathbb{E}\left[\left\|f\left(\widetilde{w}^{r+1}_{+\frac{1}{2}}\right)\right\|^2\right] \\
&\overset{(2)}{\leq} \frac{f\left(\widetilde{w}^{r}_{+\frac{1}{2}}\right) - f\left(\widetilde{w}^{r+1}_{+\frac{1}{2}}\right)}{CK\eta_g\eta_L R} + \frac{1}{C}\left(10KL^4\eta_L^2\rho^2\sigma_l^2 + 90K^2L^2\eta_L^2\sigma_g^2 + 180K^2L^4\eta_L^2\rho^2 + 24K^4L^6\eta_L^6\rho^2\alpha_{g_s}\right. \\
&\left. + 16K^3L^6\eta_L^4\rho^2\alpha_{g_s} + \frac{\eta_g\eta_L L^3\rho^2}{N}\left(\sigma_l^2 + G_s^2\right)\right) \\
&\overset{(3)}{\leq} \frac{f\left(\widetilde{w}^{0}_{+\frac{1}{2}}\right) - f^*}{CK\eta_g\eta_L R} + \frac{1}{C}\left(10KL^4\eta_L^2\rho^2\sigma_l^2 + 90K^2L^2\eta_L^2\sigma_g^2 + 180K^2L^4\eta_L^2\rho^2 + 24K^4L^6\eta_L^6\rho^2\alpha_{g_s}\right. \\
&\left. + 16K^3L^6\eta_L^4\rho^2\alpha_{g_s} + \frac{\eta_g\eta_L L^3\rho^2}{N}\left(\sigma_l^2 + G_s^2\right)\right).
\end{aligned}$$

(1) is due to Assumption 1 and taking expectation of $f\left(\widetilde{w}^{r+1}_{+\frac{1}{2}}\right)$ over randomness at global round r. (2) is from transposition of Lemma 4.10, summing it for $r = [R]$, and multiplying both sides by $\frac{1}{CK\eta_g\eta_L R}$ with $0 < C < \frac{1}{2} - 30K^2L^2\eta_L^2$ if $\eta_L < \frac{1}{\sqrt{30}KL}$. (3) is from the relationship between current loss and optimal loss $f\left(\widetilde{w}^{r+1}_{+\frac{1}{2}}\right) \geq f^*$, and the one between current loss and initial loss $f\left(\widetilde{w}^{0}_{+\frac{1}{2}}\right) \geq f\left(\widetilde{w}^{r}_{+\frac{1}{2}}\right)$. If we choose local and global learning rates as $\eta_l = \frac{1}{\sqrt{RKL}}$ and $\eta_G = \sqrt{KN}$, and choose perturbation radius as $\rho = \frac{1}{\sqrt{R}}$, we have:

$$\frac{1}{R}\sum_{r=1}^{R}\mathbb{E}\left[\|f\left(w^{r+1}\right)\|^2\right] = O\left(\frac{FL}{\sqrt{RKN}} + \frac{\sigma_g^2}{R} + \frac{L^2\left(\sigma_l^2 + G_s^2\right)}{R^{\frac{3}{2}}\sqrt{KN}} + \frac{L^2}{R^2} + \frac{L^2\sigma_l^2}{R^2K} + \frac{L^2\alpha_{g_s}}{R^3K} + \frac{\alpha_{g_s}}{R^4K^2}\right).$$

After neglecting high order terms, we have:

$$\frac{1}{R}\sum_{r=1}^{R}\mathbb{E}\left[\left\|f\left(w^{r+1}\right)\right\|^2\right] = O\left(\frac{FL}{\sqrt{RKN}} + \frac{\sigma_g^2}{R} + \frac{L^2\left(\sigma_l^2 + G_s^2\right)}{R^{\frac{3}{2}}\sqrt{KN}} + \frac{L^2}{R^2} + \frac{L^2\sigma_l^2}{R^2K}\right).$$

Lemma 4.11. *For the circumstance of partial client participation without replacement, the upper bound of* $\mathbb{E}_r\left[\|\Delta^r\|^2\right]$ *is:*

$$\mathbb{E}_r\left[\|\Delta^r\|^2\right] \leq \frac{2K\eta_l^2L^2\rho^2}{N}\left(\sigma_l^2 + G_S^2\right) + \frac{\eta_l^2}{NS}\sum_i\left\|\sum_{j=1}^{K-1}\nabla f_i\left(\widetilde{w}^r_{i,j+\frac{1}{2}}\right)\right\|^2 + \frac{(S-1)\eta_L^2}{SN^2}\left\|\sum_{j=0}^{K-1}\nabla f_i\left(\widetilde{w}^r_{i,j+\frac{1}{2}}\right)\right\|^2.$$

Proof.

$$\mathbb{E}_r\left[\|\Delta^r\|^2\right]$$

$$
\begin{aligned}
&\overset{(1)}{\leq} \frac{\eta_l^2}{S^2}\mathbb{E}_r\left[\left\|\sum_{i\in S^r}\sum_k \tilde{g}_{i,k+\frac{1}{2}}\right\|^2\right] \\
&= \frac{\eta_l^2}{S^2}\mathbb{E}_r\left[\left\|\sum_i \mathbb{1}\left\{i\in S^r\right\}\sum_k \tilde{g}_{i,k+\frac{1}{2}}\right\|^2\right] \\
&\overset{(2)}{\leq} \frac{\eta_l^2}{NS}\mathbb{E}_r\left[\left\|\sum_i\sum_{j=0}^{K-1}\left(\tilde{g}_{i,j+\frac{1}{2}} - \nabla f_i\left(\widetilde{w}^r_{i,j+\frac{1}{2}}\right)\right)\right\|^2\right] + \frac{\eta_l^2}{S^2}\mathbb{E}_r\left[\left\|\sum_i \mathbb{1}\{i\in S^r\}\sum_{j=0}^{K-1}\nabla f_i\left(\widetilde{w}^r_{i,j+\frac{1}{2}}\right)\right\|^2\right] \\
&\overset{(3)}{\leq} \frac{2K\eta_l^2L^2\rho^2}{N}\left(\sigma_l^2+G_s^2\right) + \frac{\eta_l^2}{S^2}\mathbb{E}_r\left[\left\|\sum_{i=1}^{s}\sum_{j=0}^{K-1}\nabla f_i\left(\widetilde{w}^r_{i,j+\frac{1}{2}}\right)\right\|^2\right] \\
&\overset{(4)}{\leq} \frac{2K\eta_l^2L^2\rho^2}{N}\left(\sigma_l^2+G_s^2\right) + \frac{\eta_l^2}{NS}\sum_i\left\|\sum_{j=1}^{K-1}\nabla f_i\left(\widetilde{w}^r_{i,j+\frac{1}{2}}\right)\right\|^2 + \frac{(S-1)\eta_L^2}{SN^2}\left\|\sum_{j=0}^{K-1}\nabla f_i\left(\widetilde{w}^r_{i,j+\frac{1}{2}}\right)\right\|^2.
\end{aligned}
$$

(1) is from Lemma 4.2. (2) is from Lemma 4.3. (3) is from Lemma 4.4. (4) is from Yang's proof [13] in 'For strategy 1' of their Theorem 2.

Lemma 4.12. *If we choose all $k\in[K]$ and $i\in[N]$ for sUSAM, the upper bound of $\sum_i\mathbb{E}\left[\left\|\sum_k\nabla f_i\left(\widetilde{w}_{i,k+\frac{1}{2}}\right)\right\|^2\right]$ is then:*

$$
\begin{aligned}
\sum_i\mathbb{E}\left[\left\|\sum_k\nabla f_i\left(\widetilde{w}_{i,k+\frac{1}{2}}\right)\right\|^2\right] \leq 30NK^2L^2\eta_L^2&\left(2L^2\rho^2\sigma_l^2+6K\left(3\sigma_g^2+6L^2\rho^2\right)+6K\left\|\nabla f\left(\widetilde{w}_{+\frac{1}{2}}\right)\right\|^2\right. \\
&\left.+144K^4L^6\eta_L^4\rho^2+12NK^4L^2\eta_L^2\rho^2\alpha_{g_s}+3NK^2\left(3\sigma_g^2+6L^2\rho^2\right)+3NK^2\left\|\nabla f\left(\widetilde{w}_{+\frac{1}{2}}\right)\right\|^2\right).
\end{aligned}
$$

Proof.

$$
\begin{aligned}
&\sum_i\mathbb{E}\left[\left\|\sum_k\nabla f_i\left(\widetilde{w}_{i,k+\frac{1}{2}}\right)\right\|^2\right] \\
&= \sum_i\mathbb{E}\left[\left\|\sum_k\nabla f_i\left(\widetilde{w}_{i,k+\frac{1}{2}}\right)-\nabla f_i\left(\widetilde{w}_{+\frac{1}{2}}\right)+\nabla f_i\left(\widetilde{w}_{i,k+\frac{1}{2}}\right)-\nabla f\left(\widetilde{w}_{+\frac{1}{2}}\right)+\nabla f\left(\widetilde{w}_{+\frac{1}{2}}\right)\right\|^2\right] \\
&\overset{(1)}{\leq} 6KL^2\sum_{i,k}\mathbb{E}\left[\left\|w_{i,k}-w\right\|^2\right]+6KL^2\sum_{i,k}\mathbb{E}\left[\left\|\tilde{\delta}_{i,k}-\tilde{\delta}\right\|^2\right]+3NK^2\left(3\sigma_g^2+6L^2\rho^2\right)+3NK^2\left\|\nabla f\left(\widetilde{w}_{+\frac{1}{2}}\right)\right\|^2 \\
&\overset{(2)}{\leq} 30NK^2L^2\eta_L^2\left(2L^2\rho^2\sigma_l^2+6K\left(3\sigma_g^2+6L^2\rho^2\right)+6K\left\|\nabla f\left(\widetilde{w}_{+\frac{1}{2}}\right)\right\|^2+144K^4L^6\eta_L^4\rho^2+12NK^4L^2\eta_L^2\rho^2\alpha_{g_s}\right. \\
&\left.+3NK^2\left(3\sigma_g^2+6L^2\rho^2\right)+3NK^2\left\|\nabla f\left(\widetilde{w}_{+\frac{1}{2}}\right)\right\|^2\right).
\end{aligned}
$$

(1) is from Lemma 4.2, Assumption 1, definition of perturbed model parameter, and Lemma 4.5. (2) is from Lemma 4.6 and Lemma 4.7.

Theorem 3. *Let local and global learning rates be set as $\eta_l\leq\frac{1}{10KL}$, $\eta_l\eta_G\leq\frac{1}{KL}$. Under Assumption 1, 2, 3, 4 and part client participation, the sequence of iterates generated by sUSAM satisfies:*

$$
\min_{r\in[R]}\mathbb{E}\left[\left\|\nabla f\left(w^r\right)\right\|^2\right]\leq\frac{f^0-f^*}{CK\eta_g\eta_L}+\Phi,
$$

where $\Phi = \frac{1}{C}\left(10KL^4\eta_L^2\rho^2\sigma_l^2+90K^2L^2\eta_L^2\sigma_g^2+180K^2L^4\eta_L^2\rho^2+24K^4L^6\eta_L^6\rho^2+2K^2L^4\eta_L^2\rho^2\alpha_{g_s}+16K^3L^6\eta_L^4\rho^2+\frac{\eta_g\eta_L L^3\rho^2}{2S}\left(\sigma_l^2+G_s^2\right)+\frac{\eta_g\eta_L}{S}\left(30KL^5\eta_L^2\rho^2\sigma_l^2+270K^2L^3\eta_L^2\sigma_g^2+540K^2L^5\eta_L^2\rho^2+72K^3L^7\eta_L^4\rho^2+6K^3L^3\eta_L^2\rho^2\alpha_{g_s}+\frac{9}{2}KL\sigma_g^2+9KL^3\rho^2\right)\right)$. *If local and global learning rates are chosen as $\eta_l=\frac{1}{\sqrt{RKL}}$ and*

$\eta_G = \sqrt{KS}$, *and perturbation radius is chosen as* $\rho = \frac{1}{\sqrt{R}}$, *the convergence rate can be expressed as:*

$$\frac{1}{R}\sum_{r=1}^{R}\mathbb{E}\left[\left\|f\left(w^{r+1}\right)\right\|^2\right] = O\left(\frac{FL}{\sqrt{RKS}} + \frac{\sqrt{K}\sigma_g^2}{\sqrt{RS}} + \frac{\sigma_g^2}{R} + \frac{L^2\left(\sigma_l^2 + G_s^2\right) + \sigma_g^2}{R^{\frac{3}{2}}\sqrt{KS}} + \frac{\sqrt{K}L^2}{R^{\frac{3}{2}}} + \frac{L^2}{R^2}\left(1 + \alpha_{g_s}\right)\right).$$

Proof.

$$\begin{aligned}
&\mathbb{E}\left\|f\left(\widetilde{w}^{r+1}_{+\frac{1}{2}}\right)\right\| \\
&\overset{(1)}{\leq} f\left(\widetilde{w}^{r}_{+\frac{1}{2}}\right) - \frac{K\eta_g\eta_L}{2}\left\|\nabla f\left(\widetilde{w}^{r}_{+\frac{1}{2}}\right)\right\|^2 + K\eta_g\eta_L L^2\varepsilon_w + K\eta_g\eta_L L^2\varepsilon_\delta - \frac{K\eta_g\eta_L}{2}\mathbb{E}_r\left\|\sum_{i,k}\nabla f_i\left(\widetilde{w}^{r}_{i,k+\frac{1}{2}}\right)\right\|^2 \\
&+ \frac{L}{2}\eta_g^2\mathbb{E}_r\left[\|\Delta^r\|^2\right] \\
&\overset{(2)}{\leq} f\left(\widetilde{w}^{r}_{+\frac{1}{2}}\right) - \frac{K\eta_g\eta_L}{2}\left\|\nabla f\left(\widetilde{w}^{r}_{+\frac{1}{2}}\right)\right\|^2 + K\eta_g\eta_L L^2\varepsilon_w + K\eta_g\eta_L L^2\varepsilon_\delta - \frac{K\eta_g\eta_L}{2}\mathbb{E}_r\left\|\sum_{i,k}\nabla f_i\left(\widetilde{w}^{r}_{i,k+\frac{1}{2}}\right)\right\|^2 \\
&+ \frac{K\eta_g^2\eta_l^2L^3\rho^2}{2S}\left(\sigma_l^2 + G_s^2\right) + \frac{\eta_g^2LS}{2N}\sum_i\left\|\sum_{j=1}^{K-1}\nabla f_i\left(\widetilde{w}^{r}_{i,j+\frac{1}{2}}\right)\right\|^2 + \frac{\eta_g^2LS(S-1)}{2N^2}\left\|\sum_{j=0}^{K-1}\nabla f_i\left(\widetilde{w}^{r}_{i,j+\frac{1}{2}}\right)\right\|^2 \\
&\overset{(3)}{\leq} f\left(\widetilde{w}^{r}_{+\frac{1}{2}}\right) - \frac{K\eta_g\eta_L}{2}\left\|\nabla f\left(\widetilde{w}^{r}_{+\frac{1}{2}}\right)\right\|^2 + K\eta_g\eta_L L^2\varepsilon_w + K\eta_g\eta_L L^2\varepsilon_\delta + \frac{K\eta_g^2\eta_l^2L^3\rho^2}{2S}\left(\sigma_l^2 + G_s^2\right) \\
&+ \frac{L\eta_g^2\eta_l^2}{2NS}\sum_i\left\|\sum_k\nabla f_i\left(\widetilde{w}^{r}_{i,k+\frac{1}{2}}\right)\right\|^2 \\
&\overset{(4)}{\leq} f\left(\widetilde{w}^{r}_{+\frac{1}{2}}\right) - K\eta_g\eta_L\left(\frac{1}{2} - 30K^2L^2\eta_L^2 - \frac{K\eta_g\eta_L}{2S}\left(3K + 180K^2L^4\eta_L^2\rho^2\right)\right)\left\|\nabla f\left(\widetilde{w}^{r}_{+\frac{1}{2}}\right)\right\|^2 \\
&+ K\eta_g\eta_L\left(10KL^4\eta_L^2\rho^2\sigma_l^2 + 90K^2L^2\eta_L^2\sigma_g^2 + 180K^2L^4\eta_L^2\rho^2 + 24K^4L^6\eta_L^6\rho^2 + 2K^2L^4\eta_L^2\rho^2\alpha_{g_s} + 16K^3L^6\eta_L^4\rho^2\right. \\
&\left.+\frac{\eta_g\eta_L L^3\rho^2}{2S}\left(\sigma_l^2 + G_s^2\right)\right) + \frac{K\eta_g^2\eta_L^2}{S}\left(30KL^5\eta_L^2\rho^2\sigma_l^2 + 270K^2L^3\eta_L^2\sigma_g^2 + 540K^2L^5\eta_L^2\rho^2 + 72K^3L^7\eta_L^4\rho^2\right. \\
&\left.+6K^3L^3\eta_L^2\rho^2\alpha_{g_s} + \frac{9}{2}KL\sigma_g^2 + 9KL^3\rho^2\right)\Big) \\
&\overset{(5)}{\leq} f\left(\widetilde{w}^{r}_{+\frac{1}{2}}\right) - CK\eta_g\eta_L\left\|\nabla f\left(\widetilde{w}^{r}_{+\frac{1}{r}}\right)\right\|^2 + K\eta_g\eta_L\left(10KL^4\eta_L^2\rho^2\sigma_l^2 + 90K^2L^2\eta_L^2\sigma_g^2 + 180K^2L^4\eta_L^2\rho^2\right. \\
&\left.+ 24K^4L^6\eta_L^6\rho^2 + 2K^2L^4\eta_L^2\rho^2\alpha_{g_s} + 16K^3L^6\eta_L^4\rho^2 + \frac{\eta_g\eta_L L^3\rho^2}{2S}\left(\sigma_l^2 + G_s^2\right)\right) + \frac{K\eta_g^2\eta_L^2}{S}\left(30KL^5\eta_L^2\rho^2\sigma_l^2\right. \\
&\left.+270K^2L^3\eta_L^2\sigma_g^2 + 540K^2L^5\eta_L^2\rho^2 + 72K^3L^7\eta_L^4\rho^2 + 6K^3L^3\eta_L^2\rho^2\alpha_{g_s} + \frac{9}{2}KL\sigma_g^2 + 9KL^3\rho^2\right).
\end{aligned}$$

(1) is from Lemma 4.10.(4). (2) is from Lemma 4.11. (3) is due to taking the expectation of r-th round and under the assumption that learning rates satisfy $KL\eta_g\eta_L \leq \frac{S-1}{S}$. (4) is from Lemma 4.6, Lemma 4.7, Lemma 4.12. (5) is because there exists $C > 0$, $s.t.$ $0 < C < \left(\frac{1}{2} - 30K^2L^2\eta_L^2 - \frac{K\eta_g\eta_L}{2S}(3K + 180K^2L^4\eta_L^2\rho^2)\right)$.
After Lemma 4.10.(6) for $r = [R]$ is summed and both sides are multiplied by $\frac{1}{CK\eta_g\eta_L R}$, the following holds:

$$\begin{aligned}
&\frac{1}{R}\sum_{r=1}^{R}\mathbb{E}\left[\left\|f\left(w^{r+1}\right)\right\|^2\right] \\
&\overset{(6)}{\leq} \frac{f\left(\widetilde{w}^{0}_{+\frac{1}{2}}\right) - f^*}{CK\eta_g\eta_L R} + \frac{1}{C}\left(10KL^4\eta_L^2\rho^2\sigma_l^2 + 90K^2L^2\eta_L^2\sigma_g^2 + 180K^2L^4\eta_L^2\rho^2 + 24K^4L^6\eta_L^6\rho^2 + 2K^2L^4\eta_L^2\rho^2\alpha_{g_s}\right.
\end{aligned}$$

$$+16K^3L^6\eta_L^4\rho^2+\frac{\eta_g\eta_L L^3\rho^2}{2S}\left(\sigma_l^2+G_s^2\right)+\frac{\eta_g\eta_L}{S}\Big(30KL^5\eta_L^2\rho^2\sigma_l^2+270K^2L^3\eta_L^2\sigma_g^2+540K^2L^5\eta_L^2\rho^2+72K^3L^7\eta_L^4\rho^2$$
$$+6K^3L^3\eta_L^2\rho^2\alpha_{g_s}+\frac{9}{2}KL\sigma_g^2+9KL^3\rho^2\Big)\Big).$$

(6) is from the relationship between current loss and optimal loss $f\left(\widetilde{w}^{r+1}_{+\frac{1}{2}}\right)\geq f^*$, and the one between current loss and initial loss $f\left(\widetilde{w}^{0}_{+\frac{1}{2}}\right)\geq f\left(\widetilde{w}^{r}_{+\frac{1}{2}}\right)$. If we choose local and global learning rates as $\eta_l=\frac{1}{\sqrt{RKL}}$ and $\eta_G=\sqrt{KS}$, and choose perturbation radius as $\rho=\frac{1}{\sqrt{R}}$ and $F=f\left(\widetilde{w}^{0}_{+\frac{1}{2}}\right)-f^*$, we have:

$$\frac{1}{R}\sum_{r=1}^{R}\mathbb{E}\left[\left\|f\left(w^{r+1}\right)\right\|^2\right]$$
$$\leq\frac{FL}{CK\eta_g\eta_L R}+\frac{1}{C}\Bigg(10\frac{KL^2\sigma_l^2}{R}+90\frac{\sigma_g^2}{R}+180\frac{L^2}{R^2}+24\frac{1}{R^4K^2}+2\frac{L^2}{R^2}\alpha_{g_s}+16\frac{L^2}{R^3K}+\frac{L^2}{2R^{\frac{3}{2}}\sqrt{KS}}\left(\sigma_l^2+G_s^2\right)$$
$$+30\frac{L^2\sigma_l^2}{R^{\frac{5}{2}}K^{\frac{3}{2}}\sqrt{S}}+270\frac{\sigma_g^2}{R^{\frac{3}{2}}\sqrt{SK}}+540\frac{L^2}{R^{\frac{5}{2}}\sqrt{KS}}+72\frac{L^2}{R^{\frac{7}{2}}K^{\frac{3}{2}}\sqrt{S}}+6\frac{\sqrt{K}\alpha_{g_s}}{R^{\frac{5}{2}}\sqrt{S}}+\frac{9\sqrt{K}\sigma_g^2}{2\sqrt{SR}}+9\frac{\sqrt{K}L^2}{R^{\frac{3}{2}}}\Bigg).$$

If the number of sampled clients is larger than the one of epochs and high order terms are neglected, the convergence rate of part client participation for sUSAM is:

$$\frac{1}{R}\sum_{r=1}^{R}\mathbb{E}\left[\left\|f\left(w^{r+1}\right)\right\|^2\right]=O\left(\frac{FL}{\sqrt{RKS}}+\frac{\sqrt{K}\sigma_g^2}{\sqrt{RS}}+\frac{\sigma_g^2}{R}+\frac{L^2\left(\sigma_l^2+G_s^2\right)+\sigma_g^2}{R^{\frac{3}{2}}\sqrt{KS}}+\frac{\sqrt{K}L^2}{R^{\frac{3}{2}}}+\frac{L^2}{R^2}\left(1+\alpha_{g_s}\right)\right).$$

Discussion. It can be concluded for Theorem 2 and Theorem 3 that $\frac{\sigma_g^2}{R}$ and $\frac{\sqrt{K}\sigma_g^2}{\sqrt{RS}}$ are caused by heterogeneity between clients; $\frac{L^2\sigma_l^2}{R^{\frac{3}{2}}\sqrt{KN}}$, $\frac{L^2\sigma_l^2}{R^{\frac{3}{2}}\sqrt{KS}}$, $\frac{\sqrt{K}L^2}{R^{\frac{3}{2}}}$, $\frac{L^2}{R^2}$, $\frac{L^2\sigma_l^2}{R^2K}$ are relevant to local SGD; $\frac{L^2G_S^2}{R^{\frac{3}{2}}\sqrt{KN}}$, $\frac{L^2G_S^2}{R^{\frac{3}{2}}\sqrt{KS}}$, $\frac{L^2\alpha_{gS}}{R^2}$ depend on hyper-parameters of the sparse ratio. $\frac{FL}{\sqrt{RKN}}$, $\frac{FL}{\sqrt{RKS}}$ and $\frac{\sqrt{K}\sigma_g^2}{\sqrt{RS}}$ represent main terms for the convergence rate. If learning rates are set properly, the convergence rate for sUSAM can be compatible with existing non-convex FL works. Since the mask in sUSAM constrains sparse gradients, additional square and two-thirds terms are also negligible. Besides, sUSAM is potential to generalize better by the dynamic mask, thus alleviating weight shifts in $\frac{\sigma_g^2}{R}$ and $\frac{\sqrt{K}\sigma_g^2}{\sqrt{RS}}$.

Supplementary Note 5 : Complete Algorithms

Algorithm 1 describes the local pretraining process, which can be performed at any time before the federated training stage. Each client pretrains a teacher model specific to the organ(s) based on the partially-annotated dataset. Subsequently, all clients transmit locally pretrained teacher models to the server for the future pseudo labeling stage.

Algorithm 1 UFPS(pretraining)

Require: Number of clients N; number of local epochs K; initial local models $w^1,...,w^N$; partially-annotated local datasets $D_1^p,...,D_N^p$.

for each client $i\in\{1,...,N\}$ in parallel **do**
  for $k\in 1,...,K$ **do**
    $w_i^T=$ MiniBatchUpdate(D_i^p,w_i^T)
  end for
end for
return $\{w_i^T\}_{i=1}^N$

Algorithm 2 introduces the flow of federated learning. Gradient masks and statistics for uncertainty are communicated according to r_{sUSAM} and r_{UA}, respectively. r_{UA} is used to ensure that statistics of uncertainty banks are sufficient to modify the aggregation weight. Following uncertainty-aware global aggregation and the calculation of the nonintersecting global gradient mask, the global mask is sent to clients along with the global model.

Algorithm 2 UFPS(federated training)

Require: Number of local and global rounds K and R; number of clients N; partially-annotated local datasets $D_1^p, ..., D_N^p$; pretrained teacher models $w_1^T, ..., w_N^T$; initial global model w_0^0; uncertainty banks $U_{bank}^1, ..., U_{bank}^N$; global mask M_G; local masks $M_L^1, ..., w_L^N$; local momentum gradients $G_{mo}^1, ..., G_{mo}^N$; start epoch for global mean teacher, uncertainty-aware aggregation, sUSAM $r_{GMT}, r_{UA}, r_{sUSAM}$.

server sends all teacher models w^T to all clients
for each global round $r \in \{1, ..., R\}$ **do**
    for each client $i \in \{1, ..., N\}$ in parallel **do**
        $w_{i,0}^r = w_0^r$
        for each local round $k \in \{1, ..., K\}$ **do**
            if $r < r_{sUSAM}$ **then**
                $w_{i,k}^r = \text{LocalTraining}(D_i^{PL}, w^T)$
                client i sends $w_{i,k}^r$ to server
            else if $r \geq r_{sUSAM}$ and $r < r_{UA}$ **then**
                $w_{i,k}^r, M_L^i = \text{LocalTraining}(D_i^{PL}, w^T)$
                client i sends $w_{i,k}^r, M_L^i$ to server
            else
                $w_{i,k}^r, \mu^i, \sigma^i, M_L^i = \text{LocalTraining}(D_i^{PL}, w^T)$
                client i sends $w_{i,k}^r, \mu^i, \sigma^i, M_L^i$ to server
            end if
        end for
    end for
    if $r < r_{UA}$ **then**
        $w_0^r = \sum_{i \in [N]} A_i^w w_i^r$
    else
        $\hat{A}^w \leftarrow$ server calculates aggregation weights based on μ^i, σ^i according to Equation 7
        $w_0^r = \sum_{i \in [N]} \hat{A}_i^w w_i^r$
    end if
    server sends w_0^r to all clients
    if $r \geq r_{sUSAM}$ **then**
        $M_G \leftarrow$ server updates global mask according to Equation 12
        server sends global mask M_G to all clients
    end if
end for
return w_0^*

Algorithm 3 is about the local training stage in FL. At each local round, every client first uses pretrained local teacher models to generate the pseudo label and replaces classes with available ground truth labels. During this process, the uncertainty value is also calculated with logits from teacher models. Once local training converges, one can decide whether to use the global model to get pseudo labels and refine them by pretrained teacher models. With data and pseudo labels, each client updates the local model either through vanilla SGD or sUSAM, depending on the current training stage. After completing the local training, each client uploads information according to the current global epoch.

Algorithm 4 mainly discriminates the calculation of losses. During the warm-up global epochs, only basic losses are used. Later, the weight scheduler is used to enforce the local model to concentrate on classes with specific uncertainty values. When the local model converges, aRCE loss is adopted to help escape from local minima caused by noisy pseudo labels. Note that aRCE loss only works when predictions from local models are reasonable, so we use it after the end epoch of WS.

Algorithm 3 UFPS(LocalTraining)

Require: Current local round r; batch size B; partially-annotated dataset D^P; local model w; random initialized blender node and image transform node for CMIDG; warmup epoch r_{warmup}; end epoch for weight scheduler r_{WS}; start epoch for global mean teacher, uncertainty-aware aggregation, sUSAM r_{GMT}, r_{UA}, r_{sUSAM}; updating frequency for sUSAM r_{fre}; global mask M_G.

$\mu = 0, \sigma = 0$
for each batch $\{x, y\}_{i=1}^{B} \in D^P$ **do**
  $q^T \leftarrow$ forward all teacher models $w_1^T, ..., w_N^T$ on $\{x\}_{i=1}^{B}$
  $U \leftarrow$ calculate Equation 6 through q^T
  $\tilde{q}^T \leftarrow$ merge foreground classes and post-processing background class for $\hat{q}^T$
  if $r \geq r_{GMT}$ **then**
    $\tilde{q}^G \leftarrow$ forward global model on $\{x\}_{i=1}^{B}$
    $\tilde{q} \leftarrow$ refine $\tilde{q}^G$ through $\tilde{q}^T$ according to Equation 5
    $y' \leftarrow$ merge foreground class in groud truth y into $\tilde{q}$
  end if
  if $r < r_{sUSAM}$ **then**
    $\hat{q} \leftarrow$ forward w on $\{x\}_{i=1}^{B}$
    $f = CalculateLoss(\hat{q}, y', w(U))$
    $w = w - \eta \bigtriangledown f$
  else
    $\{x_{aug}\}_{i=1}^{B} \leftarrow$ perform CMIDG on $\{x\}_{i=1}^{B}$
    $\hat{q}_{aug} \leftarrow$ forward w on $\{x_{aug}\}_{i=1}^{B}$
    $f = CalculateLoss(\hat{q}_{aug}, y', w(U))$
    if $r = r_{sUSAM}$ **then**
      $M_L \leftarrow TopKSort_{T_L} \left|\bigtriangledown f\right|$
      $G_{mo} \leftarrow$ initialize local momentum gradient
    else if $r > r_{sUSAM}$ and $r\%r_{fre} = 1$ **then**
      $M_L \leftarrow TopKSort_{T_L} \left|\bigtriangledown f\right|$
      $G_{mo} \leftarrow$ update local momentum gradient according to Equation 11
    else
      take history mask M_E as current local mask
    end if
    $\tilde{w}_{+\frac{1}{2}} \leftarrow$ perturb model parameters with sparse mask
    $\hat{q} \leftarrow$ forward $\tilde{w}_{+\frac{1}{2}}$ on $\{x\}_{i=1}^{B}$
    $\tilde{f}_{+\frac{1}{2}} = CalculateLoss(\hat{q}, y', w(U))$
    $w = w - \eta \bigtriangledown \tilde{f}_{+\frac{1}{2}}$
  end if
end for
if $r \geq r_{UA}$ **then**
  $\mu = \frac{1}{|U_{bank}|} \sum_{i=1}^{|U_{bank}|} U_{bank}^i$
  $\sigma = \frac{1}{|U_{bank}|} \sum_{i=1}^{|U_{bank}|} (U_{bank}^i - \mu)^2$
end if
if $r \geq r_{sUSAM}$ **then**
  $M_L \leftarrow argmax_{T_L} G_{mo}$
end if
if $r < r_{sUSAM}$ **then**
  return w
else if $r \geq r_{sUSAM}$ and $r < r_{UA}$ **then**
  return w, M_L
else
  return w, μ, σ, M_L
end if

Algorithm 4 CalculateLoss

Require: Model prediction $\hat{q}$; pseudo label y'; model parameter w; uncertainty based weight scheduler $w(U)$.

if $r < r_{warmup}$ **then**
    $w = w - \eta \bigtriangledown f_{Dice+BCE}(\hat{q}, y')$
else if $r \geq r_{warmup}$ and $r < r_{WS}$ **then**
    $w = w - \eta \bigtriangledown (w(U) f_{Dice+BCE}(\hat{q}, y'))$
else
    $w = w - \eta \bigtriangledown f_{Dice+BCE+RCE}(\hat{q}, y')$
end if
return f

Supplementary Note 6 : Detailed Experimental Setups

6.1. Datasets

In our experiments, we used four CT image datasets that are originally fully annotated (labels for liver, kidney (left + right), spleen, pancreas). These datasets include WORD, AbdomenCT-1K, AMOS (AMOS2022), and BTCV. In the preprocessing stage, we first crop the foreground region with Hounsfield units (HU) more than 0 with a bounding box. Image intensities are then truncated into [-500, 500] to filtrate non-organ volumes. We divide each dataset with a ratio of 7:1:2 for training, validation, and testing sets.

WORD. Whole abdominal ORgan Dataset (WORD) totally contains 150 abdominal CT volumes (30495 slices) from 150 patients before the radiation therapy. Each volume consists of 159 to 330 slices of 512×512 pixels. The in-plane resolution is 0.976 *mm* $\times$ 0.976 *mm* and slice spacing is between 2.5-3.0 *mm*, indicating that the WORD is a high-resolution dataset. The whole dataset has 16 organs with fine pixel-level annotations and scribble-based sparse annotation, including the liver, spleen, kidney (L), kidney (R), stomach, gallbladder, esophagus, duodenum, colon, intestine, adrenal, rectum, bladder, head of the femur (L), and head of the femur (R). There is a significant imbalance in the pixel distribution between large and small organs within the dataset, as shown in Fig2 in the paper of WORD. 120 volumes with annotation and 30 without annotation are published.

AbdomenCT-1K. AbdomenCT-1K totally contains 1112 abdominal CT volumes from 12 medical centers. Since the information about which center each data belongs to is privacy concerned, we do not further split this dataset into more subdatasets. Organs in this dataset are liver, kidney, spleen, and pancreas. Resolution of all CT scans is 512×512 pixels with varying pixel sizes and slice thickness between 1.25-5 *mm*. 1000 volumes with annotation and 63 without annotation are published. We pick 266 samples from 1000 labeled volumes with the smallest file size to accelerate the training process.

AMOS. AMOS consists of 500 CT and 100 MRI with 15 organs, including spleen, right kidney, left kidney, gallbladder, esophagus, liver, stomach, aorta, inferior vena cava, pancreas, right adrenal gland, left adrenal gland, duodenum, bladder, prostate/uterus. The dataset is from five domains, whereas we do not split it since no domain information is provided. 200 annotated CT images and 40 annotated MRI images are published. We only use CT images.

BTCV. BTCV provides 50 CT volumes captured during portal venous contrast phase with variable volume sizes $512 \times 512 \times$ (85-198) and field of views $280 \times 280 \times 280$ mm^3 - $500 \times 500 \times 650$ mm^3. The in-plane resolution varies from 0.54×0.54 mm^2 to 0.98×0.98 mm^2, while the slice thickness ranges from 2.5-5.0 *mm*. 13 organs are in BTCV, i.e., spleen, right kidney, left kidney, gallbladder, esophagus, liver, stomach, aorta, inferior vena cava, portal vein and splenic vein, pancreas, right adrenal gland, left adrenal gland. We merge classes of left kidney and right kidney when testing. We choose 30 training samples in divided BTCV as our out-FL client.

6.2. Training

All methods are based on generic modular UNet from nnUNet [6] with auto-mixed precision to save GPU memory and to accelerate training. We modify deep supervision brunches after decoder to ones after encoder to extract more informative features. The attribute 'tracking_running_states' of batch normalization layers is set to False, which is the same as FedAvg. The output channel number of the segmentation head is set according to the specific method.

The best global model evaluated on validation sets is used to perform testing for the final result. Only the partial target set and its inverse set (background) are used to train local teacher models. Training transformation is conducted by MONAI [2], including normalizing to [-1,1], spatial padding, random crop patch generation (pos:neg=1:1, pos=HU>0) with size (80,192,192), random rotation and flipping in x and y axis, affine transformations, grid distortion, cutout, random scaling and intensity shifting. Dice and BCE losses are used as default loss functions. Transformations for validation and testing only include normalization and spatial padding. Sliding window inference from MONAI is used with ROI size (80,192,192), sw batch size 2 and overlap 0.5. We train all methods for 500 communication rounds. In each communication round, all clients in FL are selected for one epoch of local training. We choose AdamW [8] as our optimizer with weight decay 10^{-6}. We

conduct 10 warm-up rounds from minimal learning rate 10^{-6} to the initiate learning rate 10^{-4} and change the learning rate by warm-up restart scheduler in rest rounds. All methods are implemented in PyTorch on two Nvidia GeForce RTX 3090s.

6.3. Metric

Here, we use TP, TN, FP, FN as abbreviation for true positive, true negative, false positive and false negative. Besides, x_{GT}, x_{pred} represent ground truth and model prediction, respectively.

Dice. The ratio between the intersection part of two objects and the total area:

$$\text{Dice} = \frac{2\left|x_{GT} \cap x_{pred}\right|}{\left|x_{GT}\right| + \left|x_{pred}\right|} = \frac{2TP}{2TP + FP + FN}.$$

Hausdorff Distance (HD). HD measures the similarity between the point sets $\{X, Y\}$ for segmentation border:

$$\begin{aligned} HD &= \max\left\{d_{XY}, d_{YX}\right\} \\ &= \max\left\{\max_{x\in X}\min_{y\in Y} d(x,y), \min_{y\in Y}\max_{x\in X} d(x,y)\right\}. \end{aligned}$$

Jaccard Coefficient (JC). The ratio between intersection and union:

$$JC = \frac{\left|x_{GT} \cap x_{pred}\right|}{\left|x_{GT} \cup x_{pred}\right|} = \frac{TP}{TP + FP + FN}.$$

Sensitivity (Sen). Sensitivity is also called recall, which measures the model ability to segment interested regions:

$$Sen = \frac{\left|x_{GT} \cap x_{pred}\right|}{\left|x_{GT}\right|} = \frac{TP}{TP + FN}.$$

Specificity (Spe). Specificity measures the model ability to judge uninterested regions correctly:

$$Spe = \frac{TN}{TN + FP}.$$

Relative Volume Error (RVE). The ratio between absolute error and ground truth is taken:

$$RVE = \frac{abs\left(\left|x_{GT}\right| - \left|x_{pred}\right|\right)}{\left|x_{GT}\right|}.$$

6.4. Complete model aggregation

Datasets, i.e., WORD, AbdomenCT-1K, AMOS, with partially-annotated labels are used to train the models by Dice loss and binary cross entropy (BCE) loss only for annotated foreground class(es). Except for Multi-head, Multi-encoder, and Multi-decoder, we only use foreground label(s) to calculate Dice and BCE losses.

FedAvg. Above three datasets are used to train three local models. The aggregated global model is used for evaluation.

Cond-dec. Above three datasets are used to train three local models. Client index is additionally input into the decoder after each BN layer as a hash value matching the magnitude of the BN output. Other procedures are the same with FedAvg. During validation, the same operation as the one in training is performed to get foreground classes corresponding to client indexes. For the background class, three logits for corresponding indexes are averaged in the 0-th channel.

FedBN. Above three datasets are used to train three local models. All procedures are the same as FedAvg except that batch normalization layers are personalized. During validation, the same operations as ones in Cond-dec are performed for foreground classes and the background class.

FedASAM. Above three datasets are used to train three local models. All procedures are the same as FedAvg except that methods in FedASAM are additionally used.

MENU-Net. Above three datasets are used to train local models with multi-encoders by marginal and exclusive (ME) losses. The total number of encoders is equal to the organ amount in our experiment, i.e., four encoders. During the forward pass of training, all features from the last layer of four encoders are concatenated as inputs to the decoder and all features from each layer of four encoders are concatenated as inputs to deep supervision layers. Moreover, only the encoder corresponding to the specific organ, which is the current training target, is updated (thus we update Abdomen-1K twice). It should be noted that the whole patch training strategy in MENU-Net is changed to one random patch chosen by the percentage of pos:neg = 1:1, since the time gap can be tens of times in our experiment. When the server aggregates local models, for the multi-encoder part(s), only the encoder(s) from the client owning the annotated organ label is(are) used to update the part(s) in the global model. When testing, all clients forward all encoders to get four features of the bottleneck layer as inputs to the decoder.

6.5. Part model aggregation

Datasets, i.e., WORD, AbdomenCT-1K, AMOS, with partially-annotated labels are used to train models by Dice and BCE losses only for annotated foreground class(es). Validation in this category is performed before model aggregation. For Multi-encoder and Multi-decoder, the same operation is performed for foreground class(es) and the background class, since the output channel of the segmentation head is five (four for the foreground and one for the background).

Multi-head. All procedures are the same with FedAvg except for what is mentioned above and that segmentation heads (the final convolution layer) are personalized.

Multi-encoder. All procedures are the same with FedAvg except for what is mentioned above and that encoders are personalized.

Multi-decoder. All procedures are the same with FedAvg except for what is mentioned above and that decoders are personalized.

6.6. Baseline and SOTAs

We modify existing methods to better suit our experiment and tune hyper-parameters for each method. For all methods except SOLO, Centralized, FedCRLD, and DOD*, model aggregation is conducted before validation. We evaluate them on test sets only by the global model. Unless specified, Dice and BCE losses are used to train the model(s).

SOLO (partial). Each client trains a model on the partially-annotated local dataset by Dice and BCE losses. During test time, we use three models to get three predictions and merge metrics from all of them. These models are used as pretrained teacher models for all SOTAs except FedCRLD and DOD*.

Centralized (full). Only a centralized model is trained on WORD, AbdomenCT-1K, and AMOS with fully-annotated labels.

FedCRLD. FedCRLD is a pFL method with encoders personalized, so we perform evaluation before aggregation. This method is based on contrastive learning similar to BYOL [5]. Cross-attention and self-attention of features are incorporated into the network. In addition to the segmentation loss, there are three additional losses: mean square error (MSE) loss for predictions between the current local model and the momentum local model; MSE loss for features between the current local model and the momentum local model, and between the global model and the momentum local model; knowledge distillation (KD) loss between the current local model and the global model.

DOD*. DOD is originally a PSS method designed for centralized learning. A controller is proposed to generate dynamic weights for the segmentation head. The inputs of the controller are the organ index and the bottleneck feature. The encoder and deep supervision layers are globally aggregated and the rest remains local. We perform evaluation before model aggregation for this method, just as other pFL methods.

CPS*. CPS is originally a PSS method designed for centralized learning. It is inspired by the co-training technique, in which two local models with different initializations offer extra pseudo labels for cooperative learning. We modify it into our pseudo labeling framework.

MS-KD*. MS-KD is originally a PSS method designed for centralized learning. Two losses are used: multi-scale knowledge distillation loss for original logits between the local model and teacher models, and the one for features between the local model and teacher models. Both of them use one-hot pseudo labels from teacher models to serve as foreground masks.

FedAvg*. FedAvg is originally an FL method for datasets with all class annotated. We modify it into our pseudo labeling framework. After all clients send their pretained teacher models to the server, the server sends all these models and the initialized global model to clients. Other procedures are the same with FedAvg except that each client uses pretrained teacher models to generate pseudo labels in each round.

FedProx*. FedProx is originally an FL method for datasets with all classes annotated. We modify it into our pseudo labeling framework. The MSE loss for model parameters between local and global models is used.

MOON*. MOON is originally an FL method for datasets with all classes annotated. We modify it into our pseudo labeling framework. The contrastive loss of MOON includes features from current local and global models as positive pairs and ones from the current local model and the local model in the last round as negative pairs.

FedAlign*. FedAlign is originally an FL method for datasets with all classes annotated. A regularization term of the Lipschitz constant for features between the complete local model and the slimmed local model is used.

FedASAM*. The two-step minimization method in FedASAM is the most similar to the one in our work. Original data are used in the ascent step. Stochastic weight averaging (SWA) is utilized on the server-side after model aggregation. FedASAM is modified into the pseudo label framework as FedASAM*. For ASAM, we set the start epoch as 0, perturbation radius ρ as 0.7, and balancing coefficient η as 0.001. For SWA, we tune the start epoch percentage from 0.25, 0.5, 0.75 and choose 0.75 as the best one, we also tune the number of cycles from 2,4,6 and choose 2 as the best one.

UFPS. Here we list all hyper-parameters for all modules in our method. The coefficient for aRCE loss is set to 0.01 and the start epoch is set to 200. For weight scheduler based on uncertainty scores, we choose tail shift with the value of 0.7 as the tail percentage threshold and set the end epoch for weight scheduler as 200. For uncertainty-aware global aggregation, we choose decoder as the target part and set τ^m, τ^v as 0.05, 0.001, respectively. The corresponding start epoch r_{UA} is set as 300 to accumulate enough statistics in the uncertainty bank. For global mean teacher, we choose the value of 300 as the start

epoch and the intersection percentage is set to 0.8. For sUSAM, we set the start epoch r_{sUSAM} as 300, perturbation radius ρ as 0.7, balancing coefficient η as 0.001, non-masked percentage for local mask T_L as 0.4, non-masked percentage for global mask T_L as 0.1, gradient momentum coefficient α_{mo} as 0.9, local mask update frequency r_{fre} as 5.

Supplementary Note 7 : Complete comparison between SOTAs

Unless 'Post' is written as the header of a table or 'post-processing' is marked in the title of the table, the default setting does not include any post-processing. Post-processing methods consist of filling up binary holes and removing small connected components which are less than 20% of the size of the largest non-background connected component.

Original results and ones with post-processing are demonstrated in Table S2 and Table S3, separately. Dice is considered as the most important metric in our experiment, as it directly reflects the overlap between the ground truth and the prediction. No matter whether post-processing is used, our model is able to predict accurate results for whole organs, thus getting a great result in Dice and other supplementary metrics, i.e., JC and Sen. Just achieving a large overlap is not enough, since medical diagnosis requires precise segmentation. The ability to refine segmentation borders is closely related to HD and RVE. We note that FL-based methods all suffer from excessive smoothness, thus not satisfying for the two metrics. However, they can be significantly reduced by simple post-processing techniques in practice. For Specificity, all methods achieve a great score close to 1, meaning all methods are capable of filtering out uninterested regions Therefore, the slight difference in specificity between our method and other methods is of minimal impact in real medical applications.

Supplementary Note 8 : More ablation study

Ablation for adaptive RCE. From Table S4, it can be concluded that the model performance is better when the coefficient for aRCE loss is set to a moderate value. When it is set too large, model predictions are dominant, because they are not reliable enough compared to pseudo labels. In the opposite case, noises in pseudo labels are not sufficiently alleviated.

Ablation for Weight schedulers. Here, we propose three schedulers focusing on different ranges of uncertainty values for various situations as shown in Figure S1.

Tail shift (TS).

$$w(U_j) = \begin{cases} 2 - e^{\operatorname{norm}(U_j) - \frac{r}{R}}, U > U_{\mathcal{T}}, \\ 2 - e^{\operatorname{norm}(U_j)}, \text{ else }, \end{cases}$$

where $\operatorname{norm}(U_j) = \frac{U_j - \mu}{U_{\max} - U_{\min}}$, $U_{\mathcal{T}}$ corresponds to the uncertainty value at the lowest $\mathcal{T}$ percentage. μ, U_{max}, U_{min} represent mean, maximal, minimal uncertainty values in the uncertainty bank, respectively.

Base Decrement (BD).

$$w(U_j) = 2 - \left(\frac{\alpha - e}{R} r + e\right)^{norm(U_j)},$$

where $\operatorname{norm}(U_j) = \frac{U_j - \mu}{U_{\max} - U_{\min}}$, α is a hyper-parameter determining the minimal base, and we empirically set it to 1.

Round-trip Gaussian (RG)

$$w(U_j) = (1 - \beta) \cdot G(U_j) + \beta \cdot \left(2 - e^{\operatorname{norm}(U_j)}\right),$$

$$\text{where} \quad G(U_j) = \frac{1}{\sqrt{2\pi}\varrho} \cdot e^{-\frac{\left(\operatorname{norm}(U_j) - \operatorname{norm}(\mu) - \operatorname{Range}(r)\right)^2}{2\varrho^2}},$$

$$\operatorname{Range}(r) = \begin{cases} \frac{U_{\text{range}} \cdot r}{\left\lfloor \frac{R}{2} \right\rfloor}, r \le \left\lfloor \frac{R}{2} \right\rfloor, \\ U_{\text{range}} - \frac{U_{\text{range}} \cdot \left(r - \left\lfloor \frac{R}{2} \right\rfloor\right)}{\left\lfloor \frac{R}{2} \right\rfloor}, \text{ else }, \end{cases}$$

$$U_{\text{range}} = \operatorname{norm}\left(U_{\mathcal{T}}\right) - \operatorname{norm}\left(U_{\min}\right),$$

$$\operatorname{norm}(x) = \frac{x - \mu}{U_{\max} - U_{\min}},$$

where β is a balancing factor, ϱ is used to calibrate amplitude with other schedulers, $\lfloor \cdot \rfloor$ is a rounding operator.

At the beginning of training, more certain samples are assigned to higher loss weights compared to uncertain ones, regardless of the weight scheduler used. Afterwards, TS emphasizes least confident patches, which mainly consist of noisy pseudo labels and samples from hard classes. BD equally treats each part, which is equivalent to a weight decreasement for the head part and an increasement for the middle and tail parts. RG pays less attention to the tail part, as the shift in the

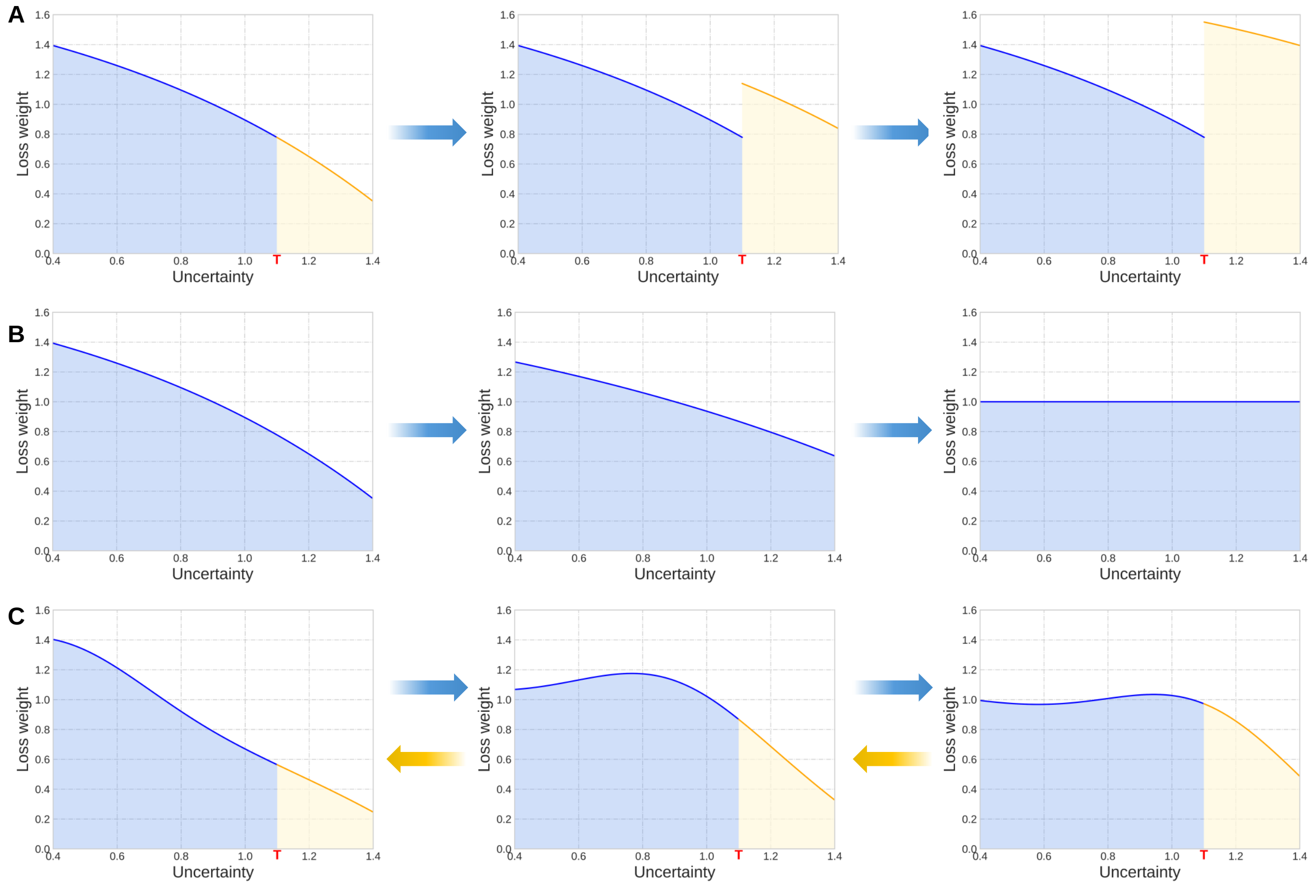

Figure S 1: Explanatory chart for three schedulers.
(A) Tail shift.
(B) Base decrement.
(C) Round-trip gaussian.

uncertainty axis is blocked before the threshold. In our experiment, the loss weight scheduler for any client is disabled during warm-up epochs r_{warmup} until the distribution for uncertainty values can be approximately estimated.

From Table S5, it can be seen that TS, focusing on the head and tail parts, achieves the best result in our experimental setting. The final epoch for weight scheduler should be decided depending on the fitting degree for different classes, which can be implicitly reflected from the model performance. Let us take TS as an example. As shown in Figure S2, the model performance on the tail class, i.e., pancreas, is relatively stable after 150 epochs. Then, the weight on the tail class should be set larger after this point. It is observed from the results that prolonging the final epoch makes little difference, as long as the noisy degree of tail classes is low. By using any of these loss weight schedulers, the convergence speed is obviously accelerated due to the concentration on uncertain samples in earlier rounds and exploration of hard but valuable ones in later stages.

Ablation for UA. Table S6 demonstrates complete results for each organ and each client under more metrics. The conclusion for the usage of different model parts is the same as what is discussed in our main paper.

To investigate the effect of changing aggregation weights on different parts of models, we use linear centralized kernel alignment [7] (Figure S3) as a measurement for the layer-wise similarity of network parameters. It can be observed that when global aggregation weights are combined with the statistics of uncertainty, the similarity between deep layers of the network is greatly changed. It indicates that the high level information related to the noise level may be learned. When UA is performed on different layers, the main difference falls around the bottleneck layer, which is related to the deepest global context.

Ablation for GMT. Table S7 demonstrates a complete result for each organ and each client under more metrics. The conclusion for the strategies is the same as what is discussed in our main paper.

In the first row of Figure S4, the intersectant percentage is over 80%, so predictions between pretrained teacher models and the global one are intersected. Although the global model over-segments some fragments and parts of segmentation borders, the over-segmented areas are eliminated with assistance from pretrained teacher models. In the third row, the circumstances happen are reversed. Thus, two kinds of main teacher models support each other by eliminating noisy points, when their

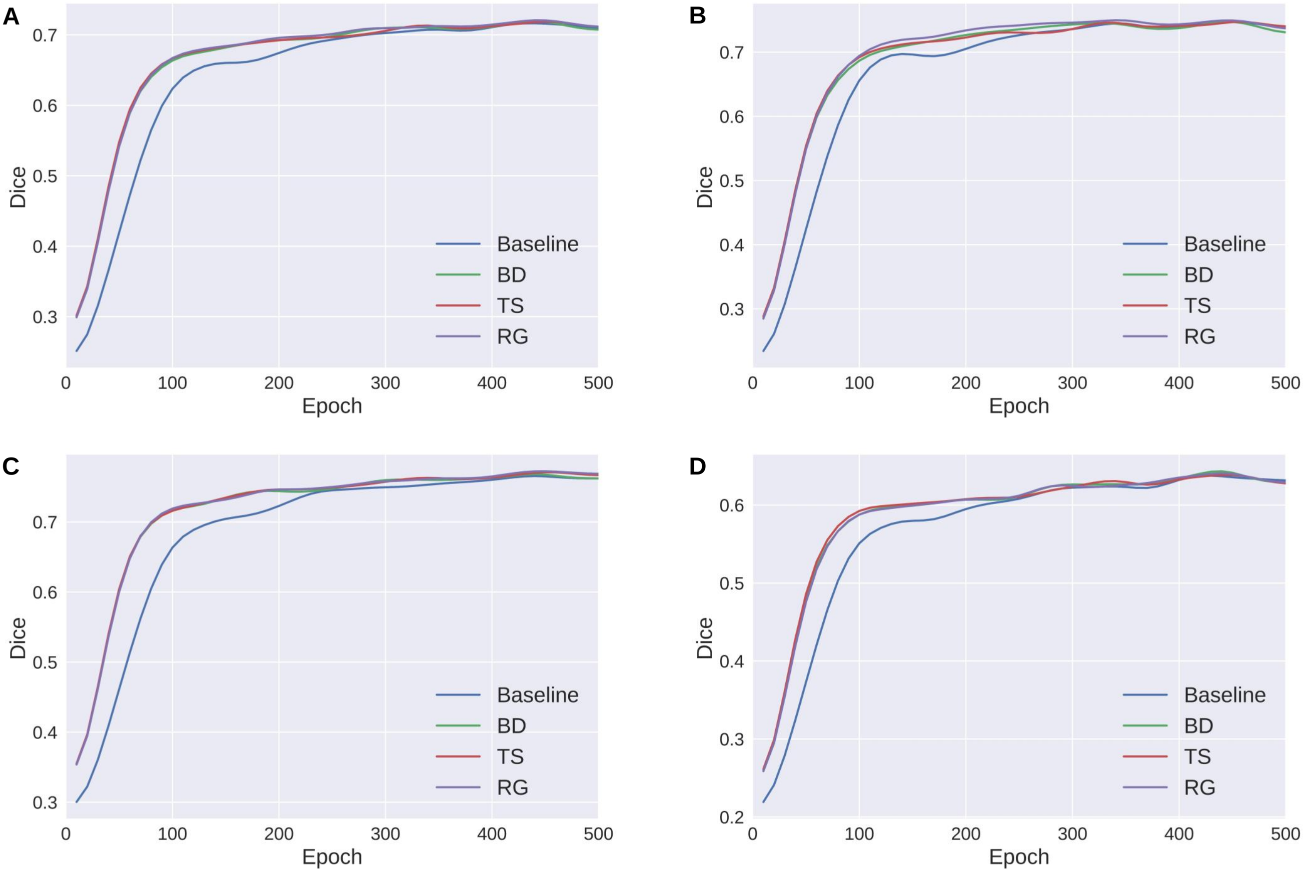

Figure S 2: Training curves for loss weight schedulers.
(A) Testing dice during training for the mean of all clients.
(B) Testing dice during training for the mean of client 1.
(C) Testing dice during training for the mean of client 2.
(D) Testing dice during training for the mean of client 3.

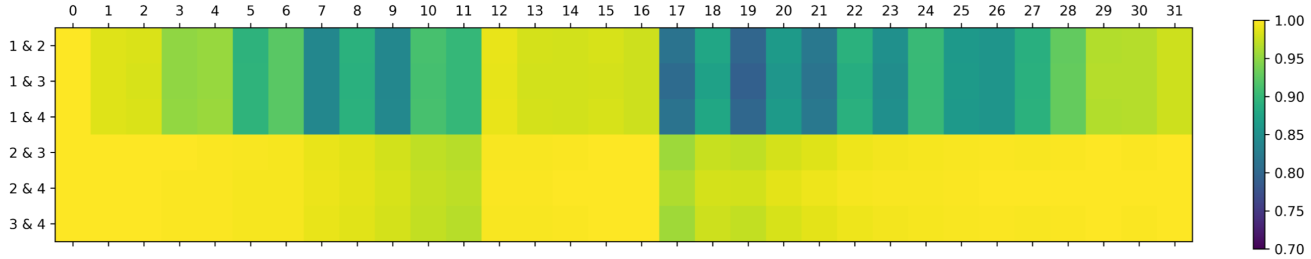

Figure S 3: Linear centered kernel alignment.
Numbers on the left side of the bar refer to method index, where FedAvg*, Whole, Encoder, Decoder are in the number sequence. Numbers above the bar are layer indexes, where encoder corresponds to 0 to 11, transpose convolution layers in decoder are from 12 to 16, vanilla convolution layers in decoder are from 17 to 26, deep supervision layers are from 27 to 30, The last one is segmentation head.

segmentations are similar. In the second row, the intersectant percentage is below 80%, so the accurate result from the global teacher is retained. This suggests that even when locally pretrained teacher models may be hindered by domain gaps sometimes, pseudo labels from the GMT module can still be convincing owing to the generalization ability of the global model.

Complete results for sUSAM. Table S8 shows a complete result for each organ and each client under more metrics. (1) is 'CMIDG from 0 epoch'. We apply CMIDG to the data of the baseline based on pseudo labels during the training process, i.e., 500 epochs, in which only descent steps are performed. (2) is 'CMIDG from 300 epoch', the mere difference between (1) and (2) is the starting epoch, which is closely related to the generated data distribution. (3) is 'Original data + ASAM', in which

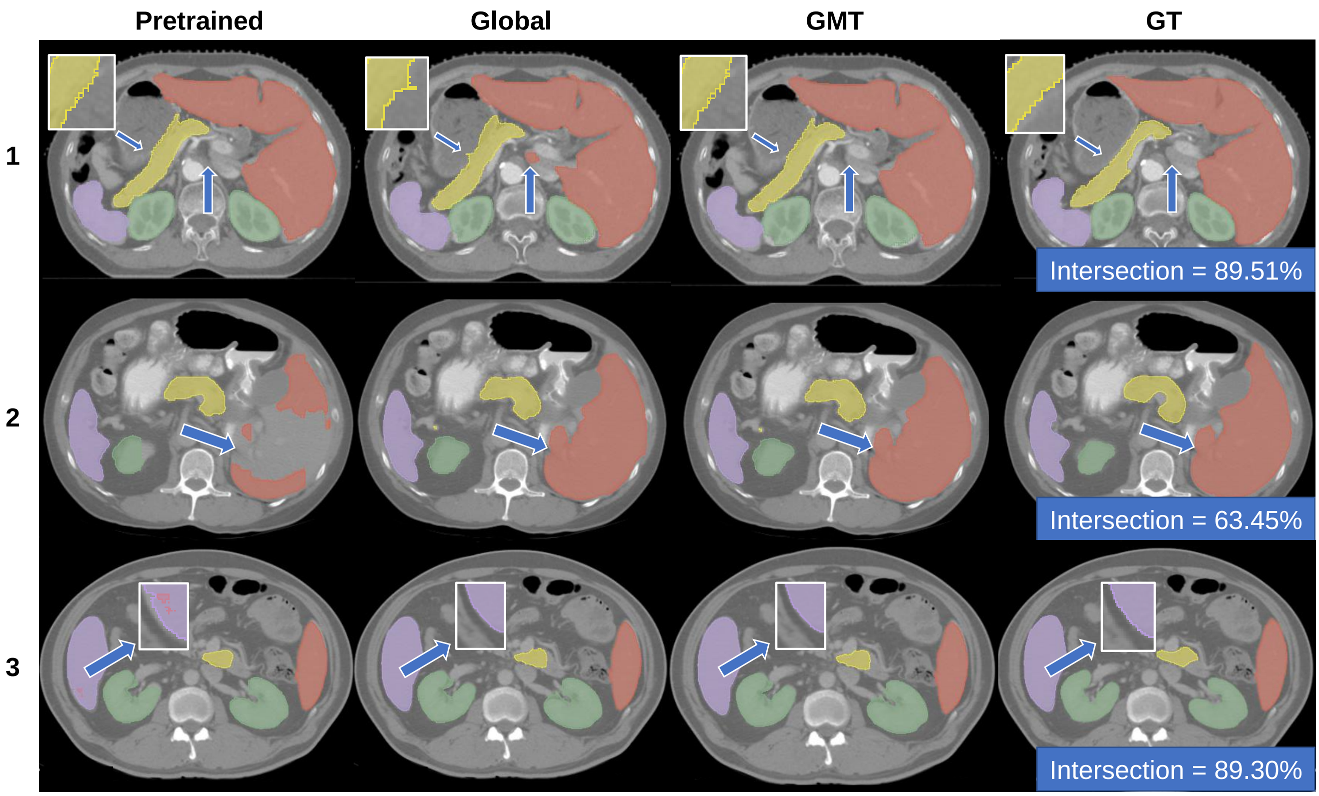

Figure S 4: Effect of global main teacher.
Numbers on the left side of images refer to client index. Green, red, purple, yellow regions represent kidney, liver, spleen and pancreas, respectively. Numbers in the blue box denotes intersection percentage between pretrained teacher models and the global one.

ascent and descend steps are both performed on the original data without CMIDG. (4) is 'Random perturbation + ASAM', in which ascent steps are only performed to get a reference to the perturbation amplitude and the perturbation follows a normal distribution. (5) is our proposed USAM, in which CMIDG is only applied to the data in the ascent step. (6) is 'USAM + (0.8 * original weight + 0.2 * perturbed weight for descent step)', in which original gradients without perturbation and perturbed gradients by CMIDG are fused according to weights to update the model in the descent step. (7) is 'CMIDG for both ascent and descent step', in which CMIDG is applied to data in both steps. (8) is 'USAM + top k perturbation' based on (5), in which the percentage of the sparse local perturbation is set to 50% and the global gradient mask is not used. (9) is our proposed sUSAM. Except (1), we use these modules only from the 300th epoch to the 500th epoch. For 'ASAM for 500 epochs', please refer to FedASAM in our SOTA comparison, in which extra modules are used in the same way as what is mentioned in the original paper. The ablation of perturbation radius hints that a moderate perturbation is better for the trade-off between generalization and stability. The conclusion for the strategies is the same as discussed in our main paper.

Table S9 displays hyper-parameter ablation for sUSAM. The conclusion for the start epoch is the same as what is discussed in our main paper. For the perturbation radius, USAM is sensitive to this hyper-parameter and 0.7 is the best one for this experimental setting.

Supplementary Note 9 : Generalization for different class divsion

Experiments in the main paper are based on one kind of setting for the class division. To prove that UFPS is universal under different class divisions, we perform more experiments on the Non-IID benchmark. Similar to the experiments in our main paper, there is no intersection between annotated classes, so the demand for labels is reduced to the lowest level.

Setting 2. In setting 2, client 1 is only annotated for spleen, client 2 is only annotated for liver, and client 3 is annotated for kidney and pancreas. In Table S10, it can be seen that although the class division has changed, our proposed method is still robust due to its universal design for the class division and strong generalization ability to different degrees of data heterogeneity.

Setting 3. In setting 3, client 1 is annotated for liver and pancreas, client 2 is only annotated for kidney, and client 3 is only annotated for spleen. The noise in this setting is the largest among three settings. In Table S11, it can be concluded that the

model performance is mainly affected by pancreas due to the domain gap. Even though the noise for pancreas is quite large, our method is capable of denoising this hard class for all clients by WS compared with other methods.

Supplementary Note 10 : Privacy Analysis

Due to extra communication compared with FedAvg, i.e., the data package and teacher models, it is crucial to discuss the underlying privacy leakage. First of all, uncertainty values in the data package are merely two numbers, so the information revealed is negligible. Local masks in data packages do not leak any more privacy than the local model because of its generation process.

However, these pretained teacher models are sent to all clients, which means either the server or other clients may utilize them to perform an attack, like membership inference attack [11]. On account of the fact that local models are uploaded to the server for the model aggregation, whether teacher models are distributed or not, it makes little difference for the attack of the server. Besides, local models can provide more information relevant to the membership because predictions for is all classes are available. Therefore, we only concentrate on the circumstance for MIA from client sides.

As we have mentioned in the experimental setting, we use a patch-cropping strategy for training. It can drastically alleviate the risk of data leakage, as proven in the previous paper[3]. Another factor influencing the vulnerability of these models to MIA is data augmentation. To give an analysis, we start from the key assumption of MIA, that is, there is a large generalization gap between training and unseen datasets for the target model. Data in the training set of a teacher model are regarded as members, while these in the validation set, testing set, and datasets owned by other clients are marked as 'not member'. Figure S5 demonstrates uncertainty distributions for three teacher models with or without data augmentation. When data augmentation is used to train a teacher model, the overall uncertainty is reduced. Besides, no matter whether data augmentation is utilized, in our experimental setting, the overall shape of any uncertainty distribution between membership and non-membership is approximate, which is adverse for MIA. We attribute the phenomenon to the patch-cropping training strategy.

Considering the above difficulty for MIA, we assume that there is an honest but curious client who is able to get half of the training dataset from the target model to train the shadow model, and that all ground-truth labels are accessible as extra input to the attack model. Note that it is a rather strong assumption, which is hard to satisfy in practice. Here we only use it to estimate a rough upper bound of the privacy risk for our method.

10.1. Experimental setup for MIA

For the shadow model, the training set is composed of half of the training dataset from the target model, 60% of the data with the lowest uncertainty values from 75% of the data of the global dataset union, and pseudo labels from the target teacher model. The testing set is composed of 40% of the data with the highest uncertainty values from the remaining data of the global dataset union. Whether data augmentation is used is identical to the setting of the target model. The training losses are Dice and BCE. Metrics are Dice and HD between predictions from the shadow model and pseudo labels from the target model.

For the attack model, the training set is composed of all data for the shadow model, predictions from the shadow model, ground-truth segmentation labels, and binary membership labels. Whether data augmentation is used is identical to the setting of the target model. The training loss is CE and metrics are class-wise accuracy and F1 score. The testing set consists of another half of the training dataset from the target model, the remaining 25% of data from the global dataset union, predictions from the shadow model, and ground-truth segmentation labels.

10.2. Performance comparison between teacher models

The comparison of uncertainty values is only relevant to prediction distributions, so we supplement experiments about the performance for in-distribution and out-distribution datasets in Table S12, where DP is a shorthand for differential privacy [4] discussed in the later analysis. It can be concluded that the domain gap for the global dataset union is extremely large but the effect of it is significantly alleviated through abundant data augmentation. Besides, the protection of DP comes from the cost of the performance degradation.

10.3. MIA attack on teacher models

The imitation performance and attack performance under the strong attack assumption are shown in Table S13. The imitation performance of shadow models is in the positive correlation with the performance of teacher models except for client 2, which proves the reasonability of predictions from teacher models. Note that when data augmentation is not used when training the teacher model from client 2, it completely fails to generalize on datasets from other clients. When segmentation results are meaningless, such as fixed simple patterns, the mode is easier to imitate to.

As for the attack performance, when data augmentation is employed, the accuracy for class 1 is sharply reduced by 24.09, indicating the attacker cannot distinguish these data in the training set from others, and all clients get better privacy protection in terms of this metric. However, the F1 score for class 1 increases by 5.93, meaning that the attack model takes more positive

predictions into account. On the other hand, two metrics in the row 'Attack class mean' are increased for 'Aug' and 'Aug + DP', suggesting that predictions for the background class are precise due to the better imitation ability. When DP is used for the privacy protection, all metrics except for the accuracy of class 1 significantly decline. It demonstrates that with a slight increasement in the accuracy of class 1 (1.07), the ability of the attack model to distinguish between both classes is limited because of the ambiguity of prediction distributions.

In conclusion, even under a strong assumption for MIA, data augmentation proves to be beneficial in protecting the membership privacy for these training data. With DP, the overall protection effect can be improved for data from all clients. Other methods, such as knowledge distillation mentioned in the previous paper[3], may also have great impacts and can be thoroughly analyzed in future studies.

Table S2. Complete comparison between SOTAs.

Method & Metric	Client index (the next line) & Organ index (after name of method)																				
	1					2					3					4					Mean
SOLO partial	1	2	3	4	mean	1	2	3	4	mean	1	2	3	4	mean	1	2	3	4	mean	
Dice	91.480	85.928	53.943	45.359	69.178	68.785	72.387	91.847	70.010	75.757	53.098	90.202	64.664	34.435	60.600	77.485	86.167	75.398	59.763	74.703	70.059
HD	0.803	1.219	1.229	0.852	1.026	3.204	6.348	1.050	1.530	3.033	2.113	1.745	1.270	1.352	1.620	1.575	2.282	1.129	1.116	1.526	1.801
JC	0.168	0.205	0.070	0.047	0.123	0.275	0.405	0.282	0.181	0.286	0.107	0.250	0.100	0.042	0.125	0.193	0.327	0.159	0.094	0.193	0.182
RVE	0.061	0.095	0.398	0.351	0.226	0.345	2.045	0.092	0.220	0.676	0.471	0.063	0.265	0.421	0.305	0.210	0.080	0.120	0.226	0.159	0.341
Sen	0.910	0.824	0.472	0.404	0.653	0.648	0.719	0.897	0.699	0.741	0.513	0.887	0.604	0.307	0.578	0.774	0.863	0.729	0.604	0.743	0.678
Spe	1.000	0.999	1.000	1.000	1.000	0.999	0.929	1.000	0.999	0.982	1.000	0.999	1.000	1.000	1.000	0.999	0.997	1.000	1.000	0.999	0.995
Centralized full	1	2	3	4	mean	1	2	3	4	mean	1	2	3	4	mean	1	2	3	4	mean	
Dice	87.979	83.599	70.348	73.130	78.764	94.994	95.440	91.348	71.520	88.325	88.422	89.558	72.715	66.019	79.179	88.684	89.583	77.146	67.706	80.780	81.762
HD	0.825	2.238	0.684	0.678	1.106	1.681	2.733	1.055	1.428	1.724	1.211	2.314	0.838	0.958	1.330	1.383	2.286	1.011	0.985	1.416	1.394
JC	0.159	0.196	0.097	0.086	0.134	0.415	0.557	0.279	0.187	0.360	0.194	0.246	0.116	0.089	0.161	0.232	0.343	0.164	0.109	0.212	0.217
RVE	0.090	0.222	0.278	0.120	0.178	0.044	0.032	0.076	0.216	0.092	0.078	0.114	0.166	0.186	0.136	0.416	0.085	0.114	0.167	0.196	0.151
Sen	0.850	0.889	0.642	0.719	0.775	0.957	0.969	0.896	0.700	0.880	0.892	0.919	0.697	0.641	0.787	0.921	0.913	0.762	0.672	0.817	0.815
Spe	1.000	0.996	1.000	1.000	0.999	0.999	0.998	1.000	1.000	0.999	1.000	0.997	1.000	1.000	0.999	0.999	0.996	1.000	1.000	0.999	0.999
FedCRLD	1	2	3	4	mean	1	2	3	4	mean	1	2	3	4	mean	1	2	3	4	mean	
Dice	89.638	79.277	62.261	40.113	67.822	71.392	79.237	93.171	67.283	77.771	56.759	84.789	61.519	29.407	58.119	74.840	84.667	71.439	58.277	72.306	69.004
HD	1.155	5.394	1.283	1.020	2.213	3.402	6.175	1.092	1.580	3.062	2.560	2.795	1.645	1.253	2.063	2.021	2.721	1.448	1.201	1.848	2.297
JC	0.162	0.182	0.084	0.042	0.118	0.284	0.441	0.288	0.172	0.296	0.111	0.228	0.095	0.035	0.117	0.181	0.317	0.149	0.091	0.185	0.179
RVE	0.090	0.302	0.287	0.344	0.256	0.340	1.634	0.067	0.268	0.577	0.345	0.182	0.345	0.520	0.348	0.188	0.088	0.166	0.242	0.171	0.338
Sen	0.893	0.852	0.575	0.359	0.670	0.681	0.784	0.933	0.688	0.772	0.529	0.874	0.589	0.259	0.563	0.746	0.846	0.700	0.596	0.722	0.682
Spe	1.000	0.995	1.000	1.000	0.999	0.998	0.945	1.000	0.999	0.986	0.999	0.995	1.000	1.000	0.999	0.999	0.996	1.000	1.000	0.999	0.995
DOD*	1	2	3	4	mean	1	2	3	4	mean	1	2	3	4	mean	1	2	3	4	mean	
Dice	86.762	83.903	54.178	22.977	61.955	79.231	89.113	92.128	66.570	81.760	51.877	89.853	64.434	36.129	60.573	73.214	85.997	69.744	53.529	70.621	70.832
HD	0.996	1.328	1.454	0.829	1.152	3.117	4.607	1.117	1.534	2.594	2.005	1.789	1.673	1.315	1.695	1.916	2.423	1.437	1.142	1.729	1.857
JC	0.154	0.199	0.067	0.023	0.111	0.320	0.503	0.281	0.171	0.319	0.104	0.247	0.096	0.044	0.123	0.176	0.325	0.142	0.083	0.182	0.186
RVE	0.101	0.097	0.653	0.713	0.391	0.214	0.433	0.080	0.242	0.242	0.472	0.066	0.333	0.451	0.331	0.287	0.083	0.193	0.289	0.213	0.271
Sen	0.831	0.799	0.551	0.179	0.590	0.759	0.890	0.902	0.645	0.799	0.481	0.880	0.612	0.348	0.580	0.710	0.851	0.677	0.522	0.690	0.693
Spe	1.000	0.999	0.999	1.000	0.999	0.999	0.987	1.000	1.000	0.996	1.000	0.999	0.999	1.000	0.999	0.999	0.997	0.999	1.000	0.999	0.997
CPS*	1	2	3	4	mean	1	2	3	4	mean	1	2	3	4	mean	1	2	3	4	mean	
Dice	89.739	89.348	75.950	48.116	75.788	73.501	77.192	93.272	68.266	78.058	59.186	89.972	74.797	39.061	65.754	80.089	87.951	75.219	59.170	75.607	73.802
HD	1.008	1.193	0.966	0.806	0.993	3.028	6.065	1.050	1.525	2.917	2.185	1.931	1.376	1.317	1.702	1.849	2.293	1.336	1.146	1.656	1.817
JC	0.163	0.217	0.106	0.051	0.134	0.302	0.437	0.288	0.176	0.301	0.121	0.249	0.117	0.048	0.134	0.200	0.335	0.158	0.092	0.196	0.191
RVE	0.068	0.055	0.174	0.293	0.147	0.290	2.112	0.089	0.221	0.678	0.411	0.098	0.246	0.391	0.287	0.270	0.075	0.153	0.197	0.174	0.322
Sen	0.884	0.883	0.698	0.432	0.724	0.702	0.779	0.933	0.677	0.773	0.578	0.901	0.711	0.359	0.637	0.820	0.891	0.734	0.595	0.760	0.724
Spe	1.000	0.999	1.000	1.000	1.000	0.999	0.927	1.000	0.999	0.981	0.999	0.997	1.000	1.000	0.999	0.999	0.996	1.000	1.000	0.999	0.995
MS-KD*	1	2	3	4	mean	1	2	3	4	mean	1	2	3	4	mean	1	2	3	4	mean	
Dice	89.920	88.511	71.978	46.995	74.351	72.861	75.769	90.539	66.100	76.317	55.820	89.298	69.066	39.118	63.326	77.914	86.683	71.644	56.173	73.104	71.775
HD	0.899	1.282	1.020	0.813	1.004	2.978	6.159	1.209	1.585	2.983	2.088	1.880	1.360	1.336	1.666	1.777	2.332	1.323	1.132	1.641	1.824
JC	0.163	0.214	0.098	0.050	0.131	0.298	0.428	0.277	0.168	0.293	0.114	0.246	0.106	0.048	0.128	0.193	0.328	0.149	0.086	0.189	0.185
RVE	0.067	0.071	0.191	0.268	0.149	0.319	1.955	0.141	0.240	0.664	0.462	0.094	0.239	0.396	0.298	0.210	0.081	0.145	0.236	0.168	0.320
Sen	0.895	0.860	0.656	0.442	0.713	0.690	0.748	0.903	0.665	0.752	0.543	0.877	0.645	0.374	0.610	0.783	0.858	0.698	0.580	0.730	0.701
Spe	1.000	0.999	1.000	1.000	1.000	0.999	0.932	0.999	0.999	0.983	1.000	0.998	1.000	1.000	0.999	0.999	0.997	1.000	1.000	0.999	0.995
FedAvg*	1	2	3	4	mean	1	2	3	4	mean	1	2	3	4	mean	1	2	3	4	mean	
Dice	90.114	84.690	72.959	51.983	74.937	72.890	79.384	93.662	66.448	78.096	59.062	88.036	68.126	42.890	64.528	79.517	87.911	75.399	56.123	74.738	73.075
HD	0.857	2.593	0.810	0.910	1.292	2.770	6.033	0.993	1.527	2.831	1.869	2.590	1.552	1.310	1.830	1.730	2.548	1.291	1.206	1.694	1.912
JC	0.164	0.200	0.101	0.056	0.130	0.296	0.449	0.290	0.169	0.301	0.119	0.239	0.107	0.054	0.130	0.197	0.333	0.158	0.086	0.194	0.189
RVE	0.065	0.176	0.223	0.196	0.165	0.310	2.211	0.063	0.240	0.706	0.409	0.221	0.343	0.348	0.330	0.249	0.093	0.125	0.210	0.169	0.343
Sen	0.897	0.892	0.677	0.485	0.738	0.692	0.803	0.926	0.645	0.766	0.571	0.920	0.640	0.402	0.633	0.807	0.904	0.734	0.561	0.752	0.722
Spe	1.000	0.997	1.000	1.000	0.999	0.999	0.925	1.000	0.999	0.981	1.000	0.994	0.999	1.000	0.998	0.999	0.995	1.000	1.000	0.998	0.994
FedProx*	1	2	3	4	mean	1	2	3	4	mean	1	2	3	4	mean	1	2	3	4	mean	
Dice	89.394	83.151	73.447	51.684	74.419	70.491	76.653	94.049	68.145	77.334	55.708	88.826	72.188	41.519	64.560	79.392	86.664	76.453	59.897	75.601	72.979
HD	0.915	2.924	0.917	0.816	1.393	2.880	6.108	1.051	1.509	2.887	1.906	2.284	1.663	1.183	1.759	1.699	2.416	1.327	1.151	1.648	1.922
JC	0.162	0.194	0.101	0.056	0.128	0.285	0.433	0.291	0.175	0.296	0.113	0.243	0.112	0.052	0.130	0.198	0.328	0.161	0.094	0.195	0.187
RVE	0.076	0.233	0.220	0.247	0.194	0.363	2.060	0.057	0.239	0.680	0.459	0.148	0.276	0.378	0.315	0.228	0.075	0.129	0.214	0.162	0.338
Sen	0.876	0.885	0.673	0.479	0.728	0.661	0.767	0.939	0.680	0.762	0.534	0.900	0.685	0.388	0.627	0.799	0.872	0.756	0.618	0.761	0.720
Spe	1.000	0.996	1.000	1.000	0.999	0.999	0.929	1.000	0.999	0.982	1.000	0.996	0.999	1.000	0.999	0.999	0.996	1.000	1.000	0.999	0.995
MOON*	1	2	3	4	mean	1	2	3	4	mean	1	2	3	4	mean	1	2	3	4	mean	
Dice	90.051	87.758	78.271	43.931	75.003	73.214	76.661	93.128	68.591	77.899	58.463	88.662	72.081	37.228	64.109	79.703	85.711	75.293	60.303	75.252	73.066
HD	1.078	1.997	1.004	0.819	1.225	2.781	6.167	1.046	1.527	2.880	2.255	2.240	1.703	1.143	1.836	1.904	2.426	1.388	1.069	1.697	1.910
JC	0.164	0.211	0.109	0.046	0.132	0.299	0.432	0.287	0.176	0.299	0.119	0.243	0.112	0.046	0.130	0.198	0.324	0.158	0.095	0.194	0.189
RVE	0.092	0.060	0.137	0.331	0.155	0.305	2.022	0.073	0.234	0.658	0.440	0.125	0.290	0.381	0.309	0.311	0.077	0.169	0.229	0.196	0.330
Sen	0.898	0.869	0.734	0.395	0.724	0.704	0.764	0.924	0.686	0.769	0.574	0.891	0.684	0.343	0.623	0.828	0.859	0.743	0.608	0.760	0.719
Spe	1.000	0.999	1.000	1.000	0.999	0.999	0.930	1.000	0.999	0.982	0.999	0.997	0.999	1.000	0.999	0.999	0.996	1.000	1.000	0.999	0.995
FedAlign*	1	2	3	4	mean	1	2	3	4	mean	1	2	3	4	mean	1	2	3	4	mean	
Dice	89.215	85.110	73.804	52.606	75.184	70.796	76.502	94.095	66.913	77.077	56.253	88.256	73.643	39.114	64.317	81.152	87.635	76.489	59.623	76.225	73.201
HD	0.825	2.248	0.968	0.782	1.206	2.804	6.204	0.997	1.559	2.891	1.840	2.453	1.613	1.205	1.778	1.617	2.475	1.281	1.116	1.622	1.874
JC	0.161	0.202	0.101	0.057	0.130	0.282	0.430	0.292	0.170	0.294	0.113	0.240	0.115	0.048	0.129	0.202	0.332	0.161	0.093	0.197	0.188
RVE	0.073	0.099	0.207	0.272	0.163	0.387	2.124	0.068	0.257	0.709	0.451	0.142	0.281	0.426	0.325	0.169	0.069	0.149	0.226	0.153	0.338
Sen	0.866	0.863	0.689	0.482	0.725	0.652	0.759	0.941	0.670	0.755	0.532	0.897	0.705	0.370	0.626	0.810	0.884	0.756	0.611	0.765	0.718
Spe	1.000	0.998	1.000	1.000	0.999	1.000	0.927	1.000	0.999	0.981	1.000	0.996	0.999	1.000	0.999	0.999	0.996	1.000	1.000	0.999	0.995
FedASAM*	1	2	3	4	mean	1	2	3	4	mean	1	2	3	4	mean	1	2	3	4	mean	
Dice	89.823	84.635	76.024	57.602	77.021	71.257	80.009	93.295	68.073	78.159	56.630	89.340	69.946	46.494	65.603	78.792	87.480	73.454	60.859	75.146	73.982
HD	1.059	2.882	0.791	0.849	1.395	2.998	5.976	1.020	1.658	2.913	1.854	2.628	1.269	1.258	1.752	1.686	2.603	1.301	1.260	1.712	1.943
JC	0.163	0.200	0.105	0.063	0.133	0.288	0.451	0.288	0.173	0.300	0.115	0.245	0.109	0.058	0.132	0.196	0.331	0.153	0.094	0.194	0.190
RVE	0.090	0.200	0.193	0.180	0.166	0.347	2.012	0.085	0.302	0.686	0.451	0.161	0.269	0.332	0.303	0.237	0.086	0.143	0.248	0.178	0.333
Sen	0.885	0.885	0.703	0.564	0.759	0.672	0.802	0.921	0.738	0.783	0.547	0.919	0.654	0.473	0.648	0.794	0.894	0.716	0.668	0.768	0.740
Spe	1.000	0.997	1.000	1.000	0.999	0.999	0.932	1.000	0.999	0.982	1.000	0.996	1.000	1.000	0.999	0.999	0.995	1.000	0.999	0.998	0.995
UFPS(Ours)	1	2	3	4	mean	1	2	3	4	mean	1	2	3	4	mean	1	2	3	4	mean	
Dice	90.186	85.345	76.248	53.126	76.226	76.428	79.529	93.512	68.788	79.564	60.284	88.419	74.441	44.140	66.821	81.082	88.691	76.677	62.456	77.226	74.959
HD	0.954	2.534	1.530	0.798	1.454	2.626	6.015	1.027	1.615	2.821	2.279	2.847	1.864	1.182	2.043	1.847	2.518	1.364	1.176	1.726	2.011
JC	0.164	0.202	0.106	0.057	0.132	0.315	0.449	0.289	0.176	0.307	0.124	0.242	0.115	0.055	0.134	0.204	0.338	0.162	0.097	0.200	0.193
RVE	0.081	0.128	0.198	0.209	0.154	0.264	2.133	0.068	0.261	0.681	0.417	0.180	0.254	0.365	0.304	0.340	0.082	0.129	0.182	0.183	0.331
Sen	0.911	0.882	0.742	0.518	0.763	0.739	0.803	0.931	0.713	0.796	0.595	0.914	0.726	0.429	0.666	0.842	0.903	0.758	0.657	0.790	0.754
Spe	1.000	0.997	1.000	1.000	0.999	0.999	0.927	1.000	0.999	0.981	0.999	0.995	0.999	1.000	0.998	0.999	0.996	1.000	0.999	0.998	0.994

Organ indexes are numbers on the straight right side of the name of method.

Model performance is better when metrics are higher, except for HD and RVE.

The best method (except Centralized full) for each metric is marked in red.

Table S3. Complete comparison between SOTAs with post-processing.

Method & Metric	Client index (the next line) & Organ index (after name of method)																				
	1					2					3					4					Mean
SOLO partial	1	2	3	4	mean	1	2	3	4	mean	1	2	3	4	mean	1	2	3	4	mean	
Dice	91.486	86.121	54.106	44.041	68.939	68.976	70.662	91.953	70.088	75.420	52.805	90.450	65.057	33.989	60.575	77.569	86.495	75.585	59.494	74.786	69.930
HD	0.693	1.192	0.912	0.743	0.885	3.037	5.934	0.979	1.522	2.868	1.887	1.583	1.014	1.228	1.428	1.521	2.204	1.041	1.073	1.460	1.660
JC	0.168	0.206	0.071	0.046	0.123	0.277	0.398	0.283	0.181	0.285	0.107	0.251	0.101	0.041	0.125	0.193	0.329	0.160	0.094	0.194	0.182
RVE	0.059	0.092	0.436	0.382	0.242	0.357	1.860	0.090	0.224	0.633	0.477	0.054	0.273	0.440	0.311	0.216	0.076	0.119	0.227	0.160	0.336
Sen	0.909	0.825	0.470	0.392	0.649	0.646	0.705	0.897	0.697	0.736	0.510	0.888	0.604	0.302	0.576	0.773	0.863	0.729	0.601	0.742	0.676
Spe	1.000	0.999	1.000	1.000	1.000	0.999	0.936	1.000	0.999	0.984	1.000	0.999	1.000	1.000	1.000	0.999	0.997	1.000	1.000	0.999	0.996
Centralized full	1	2	3	4	mean	1	2	3	4	mean	1	2	3	4	mean	1	2	3	4	mean	
Dice	88.096	87.610	70.339	73.258	79.826	95.015	95.699	91.432	71.640	88.446	88.620	91.840	72.749	65.846	79.764	88.743	90.148	77.260	67.745	80.974	82.253
HD	0.743	1.365	0.684	0.628	0.855	1.669	2.410	1.036	1.424	1.635	1.024	1.625	0.788	0.893	1.082	1.325	2.120	0.998	0.968	1.353	1.231
JC	0.159	0.211	0.097	0.086	0.138	0.415	0.560	0.280	0.187	0.361	0.195	0.256	0.116	0.089	0.164	0.232	0.347	0.165	0.110	0.213	0.219
RVE	0.084	0.132	0.278	0.125	0.155	0.043	0.022	0.076	0.212	0.088	0.071	0.059	0.170	0.197	0.124	0.415	0.079	0.112	0.160	0.191	0.140
Sen	0.851	0.890	0.642	0.719	0.775	0.957	0.969	0.896	0.699	0.880	0.891	0.919	0.696	0.639	0.787	0.921	0.913	0.762	0.670	0.816	0.815
Spe	1.000	0.998	1.000	1.000	0.999	0.999	0.998	1.000	1.000	0.999	1.000	0.998	1.000	1.000	0.999	0.999	0.996	1.000	1.000	0.999	0.999
FedCRLD	1	2	3	4	mean	1	2	3	4	mean	1	2	3	4	mean	1	2	3	4	mean	
Dice	84.112	85.878	62.329	38.752	67.768	67.770	74.864	93.433	67.373	75.860	45.890	85.537	62.287	28.543	55.565	77.017	86.740	72.047	57.701	73.376	68.142
HD	0.821	1.638	0.905	0.821	1.046	2.793	5.796	1.017	1.563	2.793	1.829	2.512	1.116	1.159	1.654	1.556	2.402	1.163	1.081	1.550	1.761
JC	0.150	0.204	0.084	0.041	0.120	0.271	0.422	0.289	0.172	0.288	0.092	0.229	0.097	0.034	0.113	0.190	0.326	0.151	0.090	0.189	0.178
RVE	0.126	0.139	0.319	0.388	0.243	0.390	1.887	0.066	0.271	0.653	0.567	0.231	0.328	0.545	0.418	0.258	0.084	0.157	0.257	0.189	0.376
Sen	0.804	0.875	0.575	0.347	0.650	0.624	0.748	0.933	0.686	0.748	0.428	0.893	0.589	0.250	0.540	0.750	0.871	0.700	0.586	0.727	0.666
Spe	1.000	0.998	1.000	1.000	0.999	1.000	0.935	1.000	0.999	0.983	1.000	0.994	1.000	1.000	0.998	0.999	0.996	1.000	1.000	0.999	0.995
DOD*	1	2	3	4	mean	1	2	3	4	mean	1	2	3	4	mean	1	2	3	4	mean	
Dice	87.269	84.003	65.498	41.000	69.443	79.903	86.098	92.608	67.929	81.635	51.056	89.947	62.968	35.182	59.788	73.609	86.049	71.120	58.136	72.229	70.773
HD	0.789	1.247	0.969	0.769	0.944	2.687	4.955	1.078	1.590	2.578	1.767	1.744	1.209	1.147	1.467	1.634	2.259	1.255	1.075	1.556	1.636
JC	0.156	0.199	0.087	0.044	0.122	0.324	0.486	0.284	0.174	0.317	0.103	0.249	0.095	0.043	0.123	0.178	0.327	0.147	0.091	0.186	0.187
RVE	0.102	0.098	0.216	0.326	0.186	0.220	0.852	0.078	0.272	0.356	0.489	0.072	0.334	0.462	0.339	0.306	0.077	0.185	0.208	0.194	0.269
Sen	0.834	0.799	0.593	0.402	0.657	0.757	0.864	0.924	0.713	0.815	0.471	0.884	0.593	0.337	0.571	0.699	0.850	0.697	0.607	0.713	0.689
Spe	1.000	0.999	1.000	1.000	1.000	0.999	0.973	1.000	0.999	0.993	1.000	0.998	1.000	1.000	1.000	0.999	0.997	1.000	1.000	0.999	0.998
CPS*	1	2	3	4	mean	1	2	3	4	mean	1	2	3	4	mean	1	2	3	4	mean	
Dice	89.827	89.562	76.114	47.121	75.656	73.555	76.436	93.895	68.271	78.039	59.407	90.512	75.327	37.740	65.747	80.476	88.199	75.757	58.339	75.693	73.784
HD	0.805	1.158	0.660	0.720	0.836	2.894	5.609	0.958	1.511	2.743	1.710	1.783	0.917	1.208	1.404	1.564	2.185	1.148	1.092	1.498	1.620
JC	0.164	0.218	0.106	0.050	0.135	0.303	0.434	0.291	0.176	0.301	0.122	0.252	0.118	0.047	0.135	0.201	0.336	0.160	0.091	0.197	0.192
RVE	0.068	0.056	0.208	0.313	0.161	0.306	1.917	0.078	0.221	0.630	0.419	0.090	0.221	0.421	0.288	0.268	0.073	0.135	0.201	0.169	0.312
Sen	0.884	0.883	0.698	0.423	0.722	0.699	0.773	0.933	0.675	0.770	0.576	0.901	0.710	0.346	0.633	0.820	0.891	0.734	0.587	0.758	0.721
Spe	1.000	0.999	1.000	1.000	1.000	0.999	0.934	1.000	0.999	0.983	1.000	0.997	1.000	1.000	0.999	0.999	0.997	1.000	1.000	0.999	0.995
MS-KD*	1	2	3	4	mean	1	2	3	4	mean	1	2	3	4	mean	1	2	3	4	mean	
Dice	90.098	88.623	72.650	46.607	74.494	72.711	72.907	90.868	66.194	75.670	55.131	89.829	69.580	38.031	63.143	77.887	86.950	71.788	55.553	73.044	71.588
HD	0.738	1.182	0.705	0.750	0.844	2.868	5.687	1.140	1.555	2.812	1.748	1.743	0.984	1.249	1.431	1.562	2.213	1.171	1.106	1.513	1.650
JC	0.164	0.215	0.100	0.050	0.132	0.298	0.416	0.278	0.169	0.290	0.113	0.249	0.108	0.047	0.129	0.193	0.330	0.151	0.085	0.190	0.185
RVE	0.069	0.072	0.247	0.289	0.169	0.333	1.743	0.147	0.240	0.616	0.468	0.087	0.238	0.397	0.298	0.218	0.081	0.153	0.239	0.173	0.314
Sen	0.895	0.860	0.655	0.437	0.712	0.686	0.725	0.903	0.663	0.744	0.535	0.877	0.644	0.362	0.604	0.780	0.858	0.696	0.571	0.726	0.697
Spe	1.000	0.999	1.000	1.000	1.000	0.999	0.939	1.000	0.999	0.984	1.000	0.998	1.000	1.000	0.999	0.999	0.997	1.000	1.000	0.999	0.996
FedAvg*	1	2	3	4	mean	1	2	3	4	mean	1	2	3	4	mean	1	2	3	4	mean	
Dice	90.278	86.680	73.132	51.525	75.404	73.066	77.390	94.021	66.579	77.764	58.541	88.700	68.305	42.171	64.429	79.725	88.599	75.677	55.678	74.920	73.129
HD	0.727	1.443	0.677	0.730	0.894	2.652	5.732	0.942	1.500	2.707	1.613	2.122	1.090	1.088	1.478	1.558	2.252	1.065	1.097	1.493	1.643
JC	0.165	0.207	0.101	0.056	0.132	0.298	0.440	0.292	0.170	0.300	0.119	0.242	0.107	0.053	0.130	0.198	0.337	0.159	0.086	0.195	0.189
RVE	0.060	0.126	0.230	0.212	0.157	0.314	1.992	0.063	0.247	0.654	0.419	0.176	0.333	0.374	0.326	0.251	0.081	0.119	0.215	0.167	0.326
Sen	0.897	0.892	0.677	0.479	0.736	0.690	0.787	0.926	0.642	0.761	0.565	0.920	0.639	0.393	0.629	0.806	0.905	0.734	0.555	0.750	0.719
Spe	1.000	0.998	1.000	1.000	0.999	0.999	0.931	1.000	1.000	0.983	1.000	0.995	1.000	1.000	0.998	0.999	0.996	1.000	1.000	0.999	0.995
FedProx*	1	2	3	4	mean	1	2	3	4	mean	1	2	3	4	mean	1	2	3	4	mean	
Dice	89.571	86.139	73.563	51.262	75.134	70.363	74.443	94.310	68.048	76.791	55.753	89.735	72.597	40.929	64.754	79.412	86.976	76.844	59.107	75.585	73.066
HD	0.787	1.550	0.672	0.715	0.931	2.781	5.726	0.973	1.508	2.747	1.644	1.871	1.081	1.076	1.418	1.513	2.240	1.145	1.065	1.491	1.647
JC	0.163	0.205	0.101	0.056	0.131	0.285	0.424	0.293	0.175	0.294	0.113	0.247	0.114	0.051	0.131	0.199	0.330	0.163	0.093	0.196	0.188
RVE	0.073	0.151	0.236	0.260	0.180	0.375	1.826	0.055	0.252	0.627	0.466	0.108	0.247	0.399	0.305	0.233	0.075	0.115	0.220	0.161	0.318
Sen	0.876	0.886	0.672	0.475	0.727	0.657	0.751	0.939	0.677	0.756	0.533	0.900	0.683	0.382	0.625	0.796	0.872	0.756	0.608	0.758	0.717
Spe	1.000	0.998	1.000	1.000	0.999	1.000	0.937	1.000	0.999	0.984	1.000	0.997	1.000	1.000	0.999	0.999	0.997	1.000	1.000	0.999	0.995
MOON*	1	2	3	4	mean	1	2	3	4	mean	1	2	3	4	mean	1	2	3	4	mean	
Dice	90.157	88.650	79.007	42.719	75.133	73.336	75.883	93.420	68.633	77.818	58.531	89.054	72.496	37.069	64.288	80.056	86.124	75.664	59.187	75.258	73.124
HD	0.824	1.198	0.606	0.760	0.847	2.665	5.586	0.954	1.520	2.681	1.705	1.868	1.011	1.105	1.422	1.616	2.256	1.112	1.056	1.510	1.615
JC	0.164	0.215	0.110	0.045	0.134	0.301	0.430	0.289	0.177	0.299	0.120	0.244	0.113	0.046	0.131	0.200	0.326	0.159	0.093	0.195	0.190
RVE	0.088	0.062	0.168	0.348	0.166	0.312	1.798	0.074	0.241	0.606	0.442	0.106	0.264	0.410	0.306	0.304	0.075	0.153	0.237	0.192	0.318
Sen	0.897	0.869	0.734	0.385	0.721	0.702	0.759	0.924	0.684	0.767	0.572	0.891	0.683	0.337	0.621	0.825	0.859	0.743	0.595	0.756	0.716
Spe	1.000	0.999	1.000	1.000	1.000	0.999	0.938	1.000	0.999	0.984	1.000	0.997	1.000	1.000	0.999	0.999	0.997	1.000	1.000	0.999	0.996
FedAlign*	1	2	3	4	mean	1	2	3	4	mean	1	2	3	4	mean	1	2	3	4	mean	
Dice	89.234	88.484	74.082	52.271	76.018	71.218	74.609	94.943	66.882	76.913	55.168	89.198	73.160	38.166	63.923	80.834	88.516	76.812	58.904	76.267	73.280
HD	0.737	1.224	0.660	0.709	0.833	2.728	5.815	0.941	1.560	2.761	1.660	1.931	0.950	1.092	1.408	1.479	2.191	1.060	1.059	1.447	1.612
JC	0.161	0.213	0.102	0.057	0.133	0.281	0.419	0.292	0.168	0.290	0.111	0.244	0.115	0.047	0.129	0.202	0.338	0.162	0.092	0.198	0.188
RVE	0.078	0.072	0.221	0.280	0.163	0.395	1.875	0.071	0.265	0.652	0.464	0.115	0.215	0.448	0.311	0.185	0.065	0.124	0.235	0.152	0.320
Sen	0.866	0.873	0.677	0.479	0.724	0.671	0.763	0.959	0.683	0.769	0.521	0.901	0.694	0.356	0.618	0.803	0.888	0.752	0.601	0.761	0.718
Spe	1.000	0.999	1.000	1.000	1.000	1.000	0.935	1.000	0.999	0.983	1.000	0.997	1.000	1.000	0.999	0.999	0.997	1.000	1.000	0.999	0.995
FedASAM*	1	2	3	4	mean	1	2	3	4	mean	1	2	3	4	mean	1	2	3	4	mean	
Dice	89.817	88.664	76.178	57.691	78.087	71.096	78.939	93.439	68.168	77.910	56.006	90.685	69.427	45.598	65.429	78.840	88.049	73.625	60.937	75.363	74.197
HD	0.830	1.230	0.654	0.721	0.859	2.864	5.473	0.976	1.638	2.738	1.625	1.840	0.930	1.134	1.382	1.536	2.252	1.191	1.143	1.531	1.628
JC	0.163	0.215	0.106	0.063	0.136	0.288	0.447	0.289	0.174	0.300	0.114	0.251	0.109	0.057	0.133	0.196	0.335	0.154	0.095	0.195	0.191
RVE	0.087	0.074	0.214	0.157	0.133	0.359	1.809	0.089	0.292	0.637	0.459	0.120	0.280	0.342	0.300	0.241	0.076	0.148	0.243	0.177	0.312
Sen	0.885	0.886	0.703	0.562	0.759	0.669	0.794	0.921	0.737	0.780	0.539	0.919	0.649	0.463	0.643	0.792	0.894	0.714	0.664	0.766	0.737
Spe	1.000	0.999	1.000	1.000	0.999	1.000	0.938	1.000	0.999	0.984	1.000	0.997	1.000	1.000	0.999	0.999	0.996	1.000	0.999	0.999	0.995
UFPS(Ours)	1	2	3	4	mean	1	2	3	4	mean	1	2	3	4	mean	1	2	3	4	mean	
Dice	90.203	87.908	76.880	53.169	77.040	76.509	78.394	93.868	68.789	79.390	60.630	89.088	75.939	43.346	67.251	81.274	89.099	77.232	62.164	77.442	75.281
HD	0.794	1.236	0.678	0.692	0.850	2.527	5.545	0.967	1.587	2.657	1.726	2.057	0.980	1.082	1.461	1.560	2.223	1.135	1.101	1.505	1.618
JC	0.164	0.212	0.107	0.057	0.135	0.316	0.445	0.291	0.176	0.307	0.125	0.245	0.119	0.055	0.136	0.204	0.340	0.164	0.097	0.201	0.195
RVE	0.071	0.084	0.212	0.207	0.143	0.273	1.901	0.064	0.255	0.623	0.421	0.141	0.207	0.384	0.288	0.312	0.077	0.100	0.168	0.164	0.305
Sen	0.911	0.882	0.741	0.517	0.763	0.737	0.795	0.931	0.711	0.793	0.595	0.914	0.725	0.418	0.663	0.841	0.903	0.758	0.650	0.788	0.752
Spe	1.000	0.998	1.000	1.000	0.999	0.999	0.935	1.000	0.999	0.983	1.000	0.996	1.000	1.000	0.999	0.999	0.996	1.000	1.000	0.999	0.995

Organ indexes are numbers on the straight right side of the name of method.

Model performance is better when metrics are higher, except for HD and RVE.

The best method (except Centralized full) for each metric is marked in red.

Table S4. Ablation study on aRCE loss.

Strategy & Metric	Client index (the next line) & Organ index (after name of method)																				Mean
	1					2					3					4					
Fixed	1	2	3	4	mean	1	2	3	4	mean	1	2	3	4	mean	1	2	3	4	mean	
Dice	89.303	87.873	73.221	50.823	75.305	71.233	74.049	91.461	66.335	75.769	59.378	88.175	66.823	42.846	64.306	80.671	85.775	74.408	59.726	75.145	72.631
HD	0.998	1.610	0.874	0.782	1.066	2.928	6.232	1.001	1.556	2.929	2.082	2.217	1.272	1.190	1.690	1.714	2.413	1.196	1.161	1.621	1.827
JC	0.162	0.211	0.100	0.054	0.132	0.289	0.416	0.281	0.169	0.289	0.120	0.241	0.104	0.054	0.130	0.202	0.324	0.157	0.093	0.194	0.186
RVE	0.090	0.075	0.224	0.257	0.161	0.396	1.999	0.118	0.265	0.694	0.404	0.148	0.266	0.360	0.294	0.242	0.082	0.130	0.198	0.163	0.328
Sen	0.887	0.878	0.666	0.470	0.725	0.670	0.732	0.888	0.642	0.733	0.575	0.884	0.620	0.403	0.620	0.816	0.857	0.714	0.613	0.750	0.707
Spe	1.000	0.998	1.000	1.000	0.999	0.999	0.930	1.000	1.000	0.982	0.999	0.996	1.000	1.000	0.999	0.999	0.996	1.000	1.000	0.999	0.995
Increasing	1	2	3	4	mean	1	2	3	4	mean	1	2	3	4	mean	1	2	3	4	mean	
Dice	90.001	84.213	75.411	52.485	75.527	73.361	78.513	93.009	65.937	77.705	59.755	87.164	69.408	45.136	65.366	80.774	87.242	73.534	57.458	74.752	73.338
HD	0.893	2.347	0.786	0.895	1.230	2.839	6.107	0.994	1.559	2.875	1.974	2.504	1.400	1.261	1.785	1.782	2.536	1.330	1.224	1.718	1.902
JC	0.164	0.200	0.104	0.057	0.131	0.299	0.443	0.287	0.168	0.299	0.122	0.238	0.109	0.057	0.131	0.202	0.330	0.154	0.089	0.194	0.189
RVE	0.070	0.146	0.201	0.194	0.153	0.291	2.238	0.076	0.238	0.711	0.395	0.193	0.277	0.311	0.294	0.286	0.088	0.154	0.205	0.183	0.335
Sen	0.898	0.874	0.691	0.494	0.739	0.697	0.791	0.910	0.642	0.760	0.580	0.904	0.652	0.428	0.641	0.822	0.889	0.709	0.586	0.752	0.723
Spe	1.000	0.997	1.000	1.000	0.999	0.999	0.924	1.000	0.999	0.981	1.000	0.995	1.000	1.000	0.998	0.999	0.995	1.000	1.000	0.998	0.994

Coefficient & Metric	Client index (the next line) & Organ index (after name of method)																				Mean
	1					2					3					4					
0.1	1	2	3	4	mean	1	2	3	4	mean	1	2	3	4	mean	1	2	3	4	mean	
Dice	88.956	87.513	73.106	47.670	74.311	70.003	77.757	91.819	64.715	76.074	55.920	88.767	73.197	36.014	63.474	79.886	87.630	75.264	57.077	74.964	72.206
HD	0.925	2.081	0.912	0.757	1.169	2.885	6.096	1.054	1.549	2.896	1.946	2.575	1.737	1.114	1.843	1.724	2.447	1.334	1.118	1.656	1.891
JC	0.161	0.210	0.100	0.051	0.130	0.282	0.438	0.282	0.164	0.292	0.113	0.243	0.114	0.044	0.128	0.200	0.332	0.157	0.088	0.194	0.186
RVE	0.076	0.093	0.223	0.360	0.188	0.376	2.047	0.093	0.290	0.702	0.452	0.153	0.331	0.460	0.349	0.237	0.082	0.159	0.226	0.176	0.354
Sen	0.872	0.890	0.660	0.421	0.711	0.649	0.777	0.891	0.617	0.733	0.533	0.903	0.683	0.318	0.609	0.796	0.886	0.719	0.557	0.739	0.698
Spe	1.000	0.998	1.000	1.000	0.999	0.999	0.929	1.000	1.000	0.982	1.000	0.996	0.999	1.000	0.999	0.999	0.996	1.000	1.000	0.999	0.995
0.01	1	2	3	4	mean	1	2	3	4	mean	1	2	3	4	mean	1	2	3	4	mean	
Dice	90.001	84.213	75.411	52.485	75.527	73.361	78.513	93.009	65.937	77.705	59.755	87.164	69.408	45.136	65.366	80.774	87.242	73.534	57.458	74.752	73.338
HD	0.893	2.347	0.786	0.895	1.230	2.839	6.107	0.994	1.559	2.875	1.974	2.504	1.400	1.261	1.785	1.782	2.536	1.330	1.224	1.718	1.902
JC	0.164	0.200	0.104	0.057	0.131	0.299	0.443	0.287	0.168	0.299	0.122	0.238	0.109	0.057	0.131	0.202	0.330	0.154	0.089	0.194	0.189
RVE	0.070	0.146	0.201	0.194	0.153	0.291	2.238	0.076	0.238	0.711	0.395	0.193	0.277	0.311	0.294	0.286	0.088	0.154	0.205	0.183	0.335
Sen	0.898	0.874	0.691	0.494	0.739	0.697	0.791	0.910	0.642	0.760	0.580	0.904	0.652	0.428	0.641	0.822	0.889	0.709	0.586	0.752	0.723
Spe	1.000	0.997	1.000	1.000	0.999	0.999	0.924	1.000	0.999	0.981	1.000	0.995	1.000	1.000	0.998	0.999	0.995	1.000	1.000	0.998	0.994
0.001	1	2	3	4	mean	1	2	3	4	mean	1	2	3	4	mean	1	2	3	4	mean	
Dice	89.363	86.046	75.599	50.741	75.437	71.975	74.055	91.625	66.485	76.035	60.252	87.589	69.150	41.460	64.613	80.935	85.855	75.241	60.220	75.563	72.912
HD	0.985	1.913	0.917	0.810	1.157	2.996	6.236	1.035	1.554	2.955	2.038	2.228	1.369	1.245	1.720	1.775	2.398	1.279	1.163	1.654	1.872
JC	0.162	0.205	0.105	0.054	0.132	0.293	0.416	0.281	0.170	0.290	0.122	0.239	0.107	0.052	0.130	0.202	0.324	0.159	0.094	0.195	0.187
RVE	0.077	0.117	0.182	0.249	0.157	0.352	2.030	0.108	0.248	0.685	0.402	0.149	0.259	0.367	0.294	0.260	0.078	0.114	0.207	0.165	0.325
Sen	0.891	0.877	0.700	0.466	0.734	0.683	0.732	0.893	0.651	0.740	0.584	0.881	0.644	0.391	0.625	0.820	0.855	0.731	0.617	0.756	0.714
Spe	1.000	0.998	1.000	1.000	0.999	0.999	0.930	1.000	0.999	0.982	0.999	0.996	1.000	1.000	0.999	0.999	0.997	1.000	1.000	0.999	0.995

Organ indexes are numbers on the straight right side of the name of method.

Model performance is better when metrics are higher, except for HD and RVE.

Table S5. Ablation study on weight scheduler.

Strategy & Metric	Client index (the next line) & Organ index (after name of method)																				
	1					2					3					4					Mean
TS	1	2	3	4	mean	1	2	3	4	mean	1	2	3	4	mean	1	2	3	4	mean	
Dice	90.162	87.442	76.888	49.430	75.980	72.955	76.875	94.070	69.066	78.242	58.440	89.017	75.613	42.264	66.334	80.019	86.902	76.442	62.072	76.359	74.229
HD	0.936	2.275	2.318	0.817	1.587	2.889	6.066	0.989	1.526	2.867	2.111	2.070	1.629	1.175	1.746	1.768	2.366	1.389	1.107	1.658	1.965
JC	0.164	0.210	0.106	0.053	0.133	0.298	0.433	0.292	0.177	0.300	0.119	0.245	0.118	0.053	0.134	0.200	0.329	0.161	0.098	0.197	0.191
RVE	0.069	0.084	0.156	0.288	0.149	0.320	1.990	0.053	0.231	0.649	0.431	0.103	0.251	0.390	0.294	0.270	0.079	0.133	0.175	0.164	0.314
Sen	0.903	0.877	0.725	0.451	0.739	0.694	0.767	0.937	0.685	0.771	0.566	0.891	0.719	0.397	0.643	0.817	0.871	0.753	0.635	0.769	0.731
Spe	1.000	0.998	1.000	1.000	0.999	0.999	0.932	1.000	0.999	0.983	1.000	0.997	0.999	1.000	0.999	0.999	0.997	1.000	1.000	0.999	0.995
BD	1	2	3	4	mean	1	2	3	4	mean	1	2	3	4	mean	1	2	3	4	mean	
Dice	90.054	85.203	73.070	52.624	75.238	73.605	79.252	93.431	67.569	78.464	58.398	87.818	72.080	45.090	65.846	79.404	87.661	75.007	58.221	75.073	73.655
HD	0.884	2.983	1.917	0.901	1.671	2.827	6.017	1.026	1.545	2.854	1.954	2.450	1.491	1.283	1.794	1.755	2.464	1.423	1.205	1.712	2.008
JC	0.164	0.203	0.099	0.057	0.131	0.301	0.447	0.289	0.173	0.302	0.119	0.240	0.113	0.057	0.132	0.198	0.332	0.157	0.090	0.194	0.190
RVE	0.069	0.118	0.207	0.194	0.147	0.295	2.220	0.072	0.244	0.708	0.426	0.173	0.279	0.343	0.305	0.282	0.081	0.135	0.223	0.180	0.335
Sen	0.898	0.871	0.676	0.499	0.736	0.702	0.803	0.923	0.671	0.775	0.563	0.904	0.689	0.426	0.646	0.809	0.894	0.733	0.600	0.759	0.729
Spe	1.000	0.998	1.000	1.000	0.999	0.999	0.925	1.000	0.999	0.981	1.000	0.995	1.000	1.000	0.998	0.999	0.996	1.000	1.000	0.998	0.994
RG	1	2	3	4	mean	1	2	3	4	mean	1	2	3	4	mean	1	2	3	4	mean	
Dice	89.983	83.416	74.119	54.278	75.449	73.284	77.649	94.167	67.751	78.213	59.423	87.513	70.165	45.833	65.734	80.209	87.297	74.953	58.421	75.220	73.654
HD	0.944	3.106	2.341	0.971	1.840	2.872	6.073	1.071	1.555	2.893	1.995	2.415	1.944	1.309	1.916	1.742	2.485	1.513	1.220	1.740	2.097
JC	0.164	0.198	0.101	0.059	0.130	0.300	0.438	0.292	0.173	0.301	0.121	0.239	0.108	0.058	0.131	0.200	0.331	0.157	0.090	0.195	0.189
RVE	0.083	0.111	0.250	0.194	0.159	0.293	2.209	0.069	0.244	0.704	0.414	0.177	0.387	0.339	0.329	0.290	0.078	0.211	0.236	0.204	0.349
Sen	0.904	0.849	0.717	0.519	0.747	0.702	0.780	0.943	0.682	0.777	0.577	0.895	0.683	0.441	0.649	0.823	0.881	0.752	0.606	0.765	0.735
Spe	1.000	0.998	0.999	1.000	0.999	0.999	0.925	1.000	0.999	0.981	1.000	0.995	0.999	1.000	0.998	0.999	0.996	0.999	0.999	0.998	0.994

Final epoch & Metric	Client index (the next line) & Organ index (after name of method)																				
	1					2					3					4					Mean
150	1	2	3	4	mean	1	2	3	4	mean	1	2	3	4	mean	1	2	3	4	mean	
Dice	89.037	83.958	74.620	51.831	74.861	71.923	74.138	92.544	66.905	76.378	62.249	87.084	71.308	42.420	65.765	81.640	85.712	74.988	60.166	75.626	73.158
HD	1.204	3.000	2.408	0.817	1.857	2.960	6.235	1.072	1.553	2.955	2.133	2.189	1.629	1.244	1.799	1.697	2.361	1.408	1.158	1.656	2.067
JC	0.161	0.197	0.102	0.056	0.129	0.292	0.417	0.285	0.171	0.291	0.125	0.238	0.110	0.053	0.132	0.204	0.323	0.158	0.094	0.195	0.187
RVE	0.108	0.157	0.188	0.230	0.171	0.392	1.982	0.089	0.247	0.677	0.369	0.131	0.254	0.391	0.286	0.245	0.084	0.154	0.219	0.175	0.327
Sen	0.892	0.866	0.698	0.484	0.735	0.679	0.729	0.914	0.666	0.747	0.601	0.865	0.674	0.403	0.636	0.827	0.849	0.737	0.623	0.759	0.719
Spe	1.000	0.997	1.000	1.000	0.999	0.999	0.931	1.000	0.999	0.982	0.999	0.997	1.000	1.000	0.999	0.999	0.997	1.000	1.000	0.999	0.995
200	1	2	3	4	mean	1	2	3	4	mean	1	2	3	4	mean	1	2	3	4	mean	
Dice	90.162	87.442	76.888	49.430	75.980	72.955	76.875	94.070	69.066	78.242	58.440	89.017	75.613	42.264	66.334	80.019	86.902	76.442	62.072	76.359	74.229
HD	0.936	2.275	2.318	0.817	1.587	2.889	6.066	0.989	1.526	2.867	2.111	2.070	1.629	1.175	1.746	1.768	2.366	1.389	1.107	1.658	1.965
JC	0.164	0.210	0.106	0.053	0.133	0.298	0.433	0.292	0.177	0.300	0.119	0.245	0.118	0.053	0.134	0.200	0.329	0.161	0.098	0.197	0.191
RVE	0.069	0.084	0.156	0.288	0.149	0.320	1.990	0.053	0.231	0.649	0.431	0.103	0.251	0.390	0.294	0.270	0.079	0.133	0.175	0.164	0.314
Sen	0.903	0.877	0.725	0.451	0.739	0.694	0.767	0.937	0.685	0.771	0.566	0.891	0.719	0.397	0.643	0.817	0.871	0.753	0.635	0.769	0.731
Spe	1.000	0.998	1.000	1.000	0.999	0.999	0.932	1.000	0.999	0.983	1.000	0.997	0.999	1.000	0.999	0.999	0.997	1.000	1.000	0.999	0.995
250	1	2	3	4	mean	1	2	3	4	mean	1	2	3	4	mean	1	2	3	4	mean	
Dice	90.046	83.004	77.278	52.935	75.816	72.447	79.672	93.434	67.472	78.256	58.184	86.879	74.119	46.158	66.335	79.815	87.472	77.062	58.387	75.684	74.023
HD	0.905	2.335	1.494	0.945	1.420	2.858	6.040	1.101	1.562	2.890	1.908	2.517	1.866	1.315	1.902	1.724	2.545	1.511	1.231	1.753	1.991
JC	0.164	0.196	0.105	0.057	0.130	0.296	0.450	0.288	0.172	0.302	0.118	0.237	0.115	0.058	0.132	0.199	0.331	0.160	0.090	0.195	0.190
RVE	0.070	0.133	0.203	0.197	0.151	0.342	2.268	0.066	0.251	0.732	0.427	0.182	0.392	0.337	0.334	0.263	0.086	0.215	0.246	0.202	0.355
Sen	0.896	0.854	0.722	0.510	0.746	0.691	0.807	0.928	0.679	0.776	0.561	0.896	0.705	0.448	0.653	0.813	0.886	0.753	0.610	0.766	0.735
Spe	1.000	0.997	1.000	1.000	0.999	0.999	0.923	1.000	0.999	0.980	1.000	0.995	0.999	1.000	0.998	0.999	0.995	0.999	0.999	0.998	0.994

U_T & Metric	Client index (the next line) & Organ index (after name of method)																				
	1					2					3					4					Mean
0.6	1	2	3	4	mean	1	2	3	4	mean	1	2	3	4	mean	1	2	3	4	mean	
Dice	90.087	86.578	75.214	48.588	75.117	72.938	77.279	91.823	68.181	77.555	58.373	89.477	72.041	41.848	65.435	80.097	86.761	76.434	62.076	76.342	73.612
HD	0.976	2.313	1.540	0.756	1.396	2.916	6.124	1.070	1.525	2.908	2.193	2.227	1.394	1.153	1.742	1.814	2.397	1.218	1.104	1.633	1.920
JC	0.164	0.207	0.104	0.051	0.131	0.298	0.435	0.281	0.175	0.297	0.119	0.246	0.111	0.052	0.132	0.201	0.328	0.161	0.098	0.197	0.189
RVE	0.067	0.113	0.176	0.330	0.172	0.313	2.030	0.095	0.240	0.670	0.429	0.126	0.226	0.395	0.294	0.269	0.083	0.121	0.201	0.168	0.326
Sen	0.899	0.883	0.702	0.432	0.729	0.695	0.774	0.896	0.666	0.758	0.568	0.900	0.673	0.386	0.632	0.823	0.874	0.741	0.629	0.767	0.722
Spe	1.000	0.998	1.000	1.000	0.999	0.999	0.930	1.000	1.000	0.982	1.000	0.997	1.000	1.000	0.999	0.999	0.996	1.000	1.000	0.999	0.995
0.7	1					2					3					4					
Dice	90.162	87.442	76.888	49.430	75.980	72.955	76.875	94.070	69.066	78.242	58.440	89.017	75.613	42.264	66.334	80.019	86.902	76.442	62.072	76.359	74.229
HD	0.936	2.275	2.318	0.817	1.587	2.889	6.066	0.989	1.526	2.867	2.111	2.070	1.629	1.175	1.746	1.768	2.366	1.389	1.107	1.658	1.965
JC	0.164	0.210	0.106	0.053	0.133	0.298	0.433	0.292	0.177	0.300	0.119	0.245	0.118	0.053	0.134	0.200	0.329	0.161	0.098	0.197	0.191
RVE	0.069	0.084	0.156	0.288	0.149	0.320	1.990	0.053	0.231	0.649	0.431	0.103	0.251	0.390	0.294	0.270	0.079	0.133	0.175	0.164	0.314
Sen	0.903	0.877	0.725	0.451	0.739	0.694	0.767	0.937	0.685	0.771	0.566	0.891	0.719	0.397	0.643	0.817	0.871	0.753	0.635	0.769	0.731
Spe	1.000	0.998	1.000	1.000	0.999	0.999	0.932	1.000	0.999	0.983	1.000	0.997	0.999	1.000	0.999	0.999	0.997	1.000	1.000	0.999	0.995
0.8	1					2					3					4					
Dice	89.937	83.021	69.056	53.694	73.927	72.708	78.784	93.477	67.148	78.029	58.746	87.359	70.843	46.870	65.954	79.690	87.442	75.228	57.643	75.001	73.228
HD	0.885	2.836	2.019	0.929	1.667	2.786	6.049	1.010	1.553	2.849	1.884	2.503	1.611	1.310	1.827	1.714	2.486	1.354	1.228	1.695	2.010
JC	0.164	0.197	0.093	0.058	0.128	0.297	0.445	0.289	0.172	0.300	0.119	0.239	0.110	0.059	0.132	0.199	0.331	0.158	0.089	0.194	0.189
RVE	0.070	0.150	0.219	0.209	0.162	0.312	2.215	0.068	0.243	0.710	0.420	0.185	0.287	0.327	0.305	0.271	0.082	0.162	0.224	0.185	0.341
Sen	0.892	0.860	0.622	0.517	0.723	0.691	0.795	0.916	0.667	0.767	0.563	0.900	0.664	0.454	0.645	0.808	0.885	0.732	0.596	0.755	0.723
Spe	1.000	0.997	1.000	1.000	0.999	0.999	0.925	1.000	0.999	0.981	1.000	0.995	0.999	1.000	0.998	0.999	0.996	1.000	0.999	0.998	0.994

Organ indexes are numbers on the straight right side of the name of method.

Model performance is better when metrics are higher, except for HD and RVE.

Table S6. Ablation study on uncertainty-based aggregation.

Part & Metric	Client index (the next line) & Organ index (after name of method)																				
	1					2					3					4					Mean
Whole	1	2	3	4	mean	1	2	3	4	mean	1	2	3	4	mean	1	2	3	4	mean	
Dice	89.152	87.143	72.443	55.928	76.167	72.077	80.032	92.170	68.436	78.179	55.467	88.943	68.425	44.227	64.266	78.831	86.120	75.791	60.651	75.348	73.490
HD	1.019	2.313	1.171	0.927	1.357	2.959	6.003	1.079	1.642	2.921	1.933	2.425	1.845	1.370	1.893	1.734	2.569	1.306	1.260	1.717	1.972
JC	0.162	0.209	0.100	0.060	0.133	0.294	0.451	0.284	0.174	0.301	0.113	0.244	0.107	0.055	0.130	0.196	0.325	0.159	0.094	0.194	0.190
RVE	0.095	0.147	0.203	0.206	0.163	0.335	2.010	0.085	0.299	0.682	0.459	0.142	0.323	0.342	0.317	0.242	0.087	0.135	0.260	0.181	0.336
Sen	0.875	0.886	0.668	0.550	0.745	0.680	0.802	0.911	0.732	0.781	0.533	0.912	0.655	0.448	0.637	0.788	0.877	0.741	0.648	0.764	0.732
Spe	1.000	0.997	1.000	1.000	0.999	0.999	0.932	1.000	0.999	0.983	1.000	0.996	0.999	1.000	0.999	0.999	0.995	1.000	0.999	0.998	0.995
Decoder	1	2	3	4	mean	1	2	3	4	mean	1	2	3	4	mean	1	2	3	4	mean	
Dice	89.189	87.566	74.843	55.024	76.655	71.651	79.422	92.767	68.377	78.054	55.613	88.663	71.228	43.742	64.812	78.643	85.612	76.312	60.216	75.196	73.679
HD	0.988	2.413	1.342	0.849	1.398	2.940	6.004	1.133	1.629	2.927	1.973	2.468	1.906	1.249	1.899	1.750	2.574	1.356	1.226	1.726	1.988
JC	0.162	0.211	0.103	0.059	0.134	0.290	0.448	0.285	0.174	0.300	0.114	0.243	0.111	0.054	0.130	0.196	0.323	0.161	0.093	0.193	0.189
RVE	0.101	0.112	0.170	0.199	0.145	0.339	1.971	0.080	0.301	0.673	0.458	0.118	0.323	0.368	0.317	0.275	0.083	0.137	0.260	0.189	0.331
Sen	0.872	0.884	0.706	0.529	0.748	0.673	0.796	0.926	0.729	0.781	0.535	0.900	0.679	0.438	0.638	0.787	0.867	0.752	0.640	0.761	0.732
Spe	1.000	0.998	1.000	1.000	0.999	0.999	0.933	1.000	0.999	0.983	1.000	0.997	0.999	1.000	0.999	0.999	0.996	1.000	0.999	0.999	0.995
Encoder	1	2	3	4	mean	1	2	3	4	mean	1	2	3	4	mean	1	2	3	4	mean	
Dice	89.160	86.205	73.917	56.563	76.461	71.797	77.977	92.006	68.408	77.547	54.908	88.393	70.014	44.558	64.468	78.398	86.128	75.169	60.078	74.943	73.355
HD	0.988	2.688	0.829	0.966	1.368	2.876	6.055	1.053	1.626	2.903	1.993	2.466	1.514	1.278	1.813	1.791	2.597	1.357	1.288	1.758	1.961
JC	0.162	0.205	0.102	0.061	0.132	0.292	0.439	0.283	0.174	0.297	0.112	0.241	0.110	0.056	0.130	0.195	0.325	0.158	0.093	0.193	0.188
RVE	0.092	0.177	0.197	0.211	0.169	0.331	1.993	0.095	0.303	0.681	0.468	0.144	0.298	0.353	0.316	0.277	0.087	0.153	0.246	0.191	0.339
Sen	0.878	0.887	0.679	0.551	0.749	0.678	0.784	0.907	0.730	0.775	0.531	0.905	0.666	0.447	0.637	0.787	0.874	0.737	0.641	0.760	0.730
Spe	1.000	0.997	1.000	1.000	0.999	0.999	0.932	1.000	0.999	0.983	1.000	0.997	1.000	1.000	0.999	0.999	0.996	1.000	0.999	0.998	0.995

Organ indexes are numbers on the straight right side of the name of method.

Model performance is better when metrics are higher, except for HD and RVE.

Table S7. Ablation study on global main teacher.

Strategy & Metric	Client index (the next line) & Organ index (after name of method)																				
	1					2					3					4					Mean
Pretrained	1	2	3	4	mean	1	2	3	4	mean	1	2	3	4	mean	1	2	3	4	mean	
Dice	90.114	84.690	72.959	51.983	74.937	72.890	79.384	93.662	66.448	78.096	59.062	88.036	68.126	42.890	64.528	79.517	87.911	75.399	56.123	74.738	73.075
HD	0.857	2.593	0.810	0.910	1.292	2.770	6.033	0.993	1.527	2.831	1.869	2.590	1.552	1.310	1.830	1.730	2.548	1.291	1.206	1.694	1.912
JC	0.164	0.200	0.101	0.056	0.130	0.296	0.449	0.290	0.169	0.301	0.119	0.239	0.107	0.054	0.130	0.197	0.333	0.158	0.086	0.194	0.189
RVE	0.065	0.176	0.223	0.196	0.165	0.310	2.211	0.063	0.240	0.706	0.409	0.221	0.343	0.348	0.330	0.249	0.093	0.125	0.210	0.169	0.343
Sen	0.897	0.892	0.677	0.485	0.738	0.692	0.803	0.926	0.645	0.766	0.571	0.920	0.640	0.402	0.633	0.807	0.904	0.734	0.561	0.752	0.722
Spe	1.000	0.997	1.000	1.000	0.999	0.999	0.925	1.000	0.999	0.981	1.000	0.994	0.999	1.000	0.998	0.999	0.995	1.000	1.000	0.998	0.994
Global	1	2	3	4	mean	1	2	3	4	mean	1	2	3	4	mean	1	2	3	4	mean	
Dice	85.067	82.719	76.878	53.277	74.485	69.884	79.873	92.888	66.808	77.363	67.640	88.265	73.222	47.373	69.125	81.954	85.995	75.902	59.939	75.947	74.230
HD	2.812	6.117	2.720	1.854	3.376	2.858	6.063	1.290	1.598	2.952	3.772	3.205	2.803	2.008	2.947	2.755	2.984	1.914	1.686	2.335	2.903
JC	0.151	0.193	0.107	0.057	0.127	0.273	0.447	0.285	0.170	0.294	0.134	0.240	0.113	0.059	0.136	0.204	0.323	0.159	0.093	0.195	0.188
RVE	0.321	0.234	0.753	0.435	0.436	0.394	1.598	0.072	0.298	0.591	0.229	0.165	0.614	0.478	0.371	0.414	0.082	0.248	0.248	0.248	0.412
Sen	0.847	0.878	0.766	0.522	0.753	0.634	0.787	0.934	0.642	0.749	0.673	0.912	0.735	0.507	0.707	0.841	0.865	0.774	0.622	0.776	0.746
Spe	0.999	0.996	0.998	0.999	0.998	0.999	0.945	1.000	0.999	0.986	0.998	0.995	0.998	0.999	0.998	0.998	0.996	0.999	0.999	0.998	0.995
Intersection	1	2	3	4	mean	1	2	3	4	mean	1	2	3	4	mean	1	2	3	4	mean	
Dice	89.913	87.246	75.142	58.777	77.770	71.843	77.264	93.326	67.922	77.589	59.374	87.560	68.836	46.827	65.649	80.884	86.757	74.798	61.954	76.098	74.277
HD	0.923	1.928	0.900	1.001	1.188	2.771	6.104	1.003	1.546	2.856	2.123	2.729	1.629	1.379	1.965	1.805	2.536	1.338	1.274	1.738	1.937
JC	0.163	0.209	0.104	0.064	0.135	0.291	0.436	0.288	0.174	0.298	0.121	0.238	0.107	0.059	0.131	0.203	0.328	0.158	0.097	0.196	0.190
RVE	0.068	0.095	0.185	0.191	0.135	0.341	2.054	0.072	0.251	0.680	0.418	0.206	0.259	0.347	0.308	0.262	0.082	0.132	0.216	0.173	0.324
Sen	0.900	0.885	0.698	0.566	0.762	0.674	0.773	0.921	0.675	0.761	0.573	0.901	0.652	0.462	0.647	0.825	0.876	0.731	0.649	0.770	0.735
Spe	1.000	0.998	1.000	1.000	0.999	0.999	0.929	1.000	0.999	0.982	1.000	0.995	1.000	1.000	0.998	0.999	0.996	1.000	1.000	0.999	0.995

Organ indexes are numbers on the straight right side of the name of method.

Model performance is better when metrics are higher, except for HD and RVE.

Table S8. Complete result for strategies of sUSAM.

Strategy & Metric	Client index (the next line) & Organ index (after name of method)																				Mean
	1					2					3					4					
(1)	1	2	3	4	mean	1	2	3	4	mean	1	2	3	4	mean	1	2	3	4	mean	
Dice	89.056	87.866	78.359	48.093	75.844	83.081	79.221	90.457	57.690	77.612	68.886	88.081	69.481	38.603	66.263	85.400	87.724	74.219	55.043	75.597	73.829
HD	1.085	1.561	0.794	0.718	1.039	2.408	6.069	1.098	1.632	2.801	2.019	2.631	1.401	1.127	1.795	1.857	2.932	1.321	1.174	1.821	1.864
JC	0.161	0.212	0.110	0.052	0.134	0.340	0.447	0.275	0.143	0.301	0.140	0.239	0.106	0.047	0.133	0.216	0.330	0.155	0.084	0.196	0.191
RVE	0.073	0.068	0.124	0.360	0.156	0.280	2.057	0.095	0.343	0.694	0.307	0.146	0.254	0.374	0.270	0.226	0.098	0.157	0.264	0.186	0.327
Sen	0.887	0.881	0.759	0.430	0.739	0.787	0.802	0.884	0.525	0.750	0.670	0.902	0.656	0.360	0.647	0.861	0.907	0.721	0.516	0.751	0.722
Spe	1.000	0.998	1.000	1.000	0.999	1.000	0.929	1.000	1.000	0.982	0.999	0.996	1.000	1.000	0.999	0.999	0.995	1.000	1.000	0.998	0.995
(2)	1	2	3	4	mean	1	2	3	4	mean	1	2	3	4	mean	1	2	3	4	mean	
Dice	88.112	85.961	76.718	52.858	75.912	78.615	76.530	89.125	60.981	76.313	67.829	85.355	69.328	41.341	65.963	83.853	86.615	72.927	57.625	75.255	73.361
HD	1.357	1.553	1.117	0.801	1.207	2.581	6.415	1.259	1.607	2.965	2.231	3.059	1.951	1.231	2.118	1.942	3.231	1.596	1.170	1.985	2.069
JC	0.159	0.205	0.107	0.057	0.132	0.316	0.430	0.269	0.152	0.292	0.136	0.231	0.106	0.051	0.131	0.210	0.326	0.151	0.089	0.194	0.187
RVE	0.097	0.087	0.231	0.194	0.152	0.329	2.096	0.107	0.307	0.710	0.300	0.146	0.338	0.325	0.277	0.191	0.080	0.180	0.200	0.162	0.325
Sen	0.866	0.838	0.757	0.495	0.739	0.725	0.753	0.882	0.572	0.733	0.643	0.843	0.672	0.399	0.639	0.826	0.866	0.719	0.561	0.743	0.714
Spe	0.999	0.999	1.000	1.000	0.999	1.000	0.927	0.999	1.000	0.982	0.999	0.996	0.999	1.000	0.999	0.999	0.996	0.999	1.000	0.999	0.995
(3)	1	2	3	4	mean	1	2	3	4	mean	1	2	3	4	mean	1	2	3	4	mean	
Dice	89.315	84.561	75.661	50.342	74.970	71.804	76.503	93.050	66.334	76.923	58.716	87.325	70.761	41.486	64.572	81.227	85.450	74.157	59.398	75.058	72.881
HD	0.941	2.400	0.917	0.778	1.259	2.791	6.141	1.040	1.558	2.883	1.896	2.523	1.548	1.170	1.784	1.661	2.438	1.320	1.157	1.644	1.893
JC	0.162	0.200	0.105	0.054	0.130	0.290	0.432	0.287	0.169	0.295	0.119	0.237	0.109	0.051	0.129	0.204	0.322	0.156	0.093	0.193	0.187
RVE	0.070	0.127	0.176	0.272	0.161	0.349	2.033	0.074	0.252	0.677	0.420	0.159	0.274	0.368	0.305	0.227	0.080	0.165	0.203	0.169	0.328
Sen	0.879	0.865	0.711	0.458	0.728	0.669	0.757	0.923	0.653	0.751	0.559	0.877	0.678	0.393	0.627	0.813	0.853	0.734	0.605	0.751	0.714
Spe	1.000	0.998	1.000	1.000	0.999	1.000	0.930	1.000	0.999	0.982	1.000	0.996	1.000	1.000	0.999	0.999	0.996	1.000	1.000	0.999	0.995
(4)	1	2	3	4	mean	1	2	3	4	mean	1	2	3	4	mean	1	2	3	4	mean	
Dice	89.898	85.934	73.955	51.902	75.422	72.841	78.147	92.656	66.228	77.468	59.050	88.062	68.214	43.595	64.730	79.909	87.912	75.289	56.800	74.978	73.150
HD	0.886	2.052	0.793	0.891	1.155	2.840	6.088	1.001	1.541	2.868	1.969	2.508	1.400	1.315	1.798	1.763	2.508	1.233	1.225	1.682	1.876
JC	0.164	0.205	0.102	0.056	0.132	0.296	0.441	0.286	0.169	0.298	0.120	0.240	0.107	0.055	0.130	0.200	0.333	0.158	0.088	0.195	0.189
RVE	0.066	0.128	0.214	0.204	0.153	0.306	2.188	0.076	0.229	0.700	0.406	0.205	0.280	0.322	0.303	0.283	0.087	0.135	0.214	0.180	0.334
Sen	0.890	0.893	0.675	0.484	0.735	0.688	0.788	0.909	0.642	0.757	0.571	0.915	0.639	0.406	0.633	0.808	0.901	0.729	0.570	0.752	0.719
Spe	1.000	0.998	1.000	1.000	0.999	0.999	0.925	1.000	0.999	0.981	1.000	0.995	1.000	1.000	0.998	0.999	0.995	1.000	1.000	0.998	0.994
(5)	1	2	3	4	mean	1	2	3	4	mean	1	2	3	4	mean	1	2	3	4	mean	
Dice	89.529	84.727	76.069	49.922	75.062	73.099	76.691	93.864	67.759	77.853	58.920	88.507	72.711	42.560	65.675	79.970	87.155	76.630	61.661	76.354	73.736
HD	1.033	2.749	0.948	0.785	1.379	2.841	6.141	1.002	1.530	2.879	2.256	2.312	1.641	1.158	1.842	1.853	2.404	1.296	1.133	1.671	1.943
JC	0.162	0.200	0.106	0.053	0.130	0.298	0.433	0.290	0.174	0.299	0.120	0.243	0.114	0.053	0.133	0.200	0.330	0.162	0.097	0.197	0.190
RVE	0.093	0.143	0.177	0.285	0.174	0.335	2.056	0.060	0.236	0.672	0.404	0.154	0.279	0.373	0.302	0.278	0.077	0.122	0.194	0.168	0.329
Sen	0.904	0.883	0.711	0.450	0.737	0.697	0.765	0.934	0.663	0.764	0.576	0.896	0.698	0.400	0.643	0.822	0.879	0.753	0.624	0.770	0.729
Spe	1.000	0.997	1.000	1.000	0.999	0.999	0.929	1.000	1.000	0.982	0.999	0.996	0.999	1.000	0.999	0.999	0.996	1.000	1.000	0.999	0.995
(6)	1	2	3	4	mean	1	2	3	4	mean	1	2	3	4	mean	1	2	3	4	mean	
Dice	89.846	82.857	75.490	49.649	74.461	73.496	76.523	93.876	67.588	77.871	59.113	88.522	73.592	41.519	65.686	80.467	86.866	76.002	60.874	76.052	73.518
HD	1.057	3.105	1.007	0.766	1.484	2.807	6.119	1.020	1.518	2.866	2.250	2.408	1.759	1.150	1.892	1.795	2.364	1.430	1.108	1.674	1.979
JC	0.163	0.194	0.105	0.053	0.129	0.300	0.432	0.290	0.173	0.299	0.120	0.243	0.114	0.052	0.132	0.201	0.329	0.160	0.096	0.197	0.189
RVE	0.091	0.195	0.194	0.299	0.195	0.317	2.013	0.065	0.242	0.659	0.411	0.141	0.313	0.392	0.314	0.296	0.078	0.153	0.186	0.178	0.337
Sen	0.902	0.878	0.712	0.447	0.735	0.703	0.764	0.939	0.652	0.765	0.578	0.892	0.703	0.384	0.639	0.832	0.872	0.755	0.606	0.766	0.726
Spe	1.000	0.997	1.000	1.000	0.999	0.999	0.931	1.000	1.000	0.982	0.999	0.996	0.999	1.000	0.999	0.999	0.996	1.000	1.000	0.999	0.995
(7)	1	2	3	4	mean	1	2	3	4	mean	1	2	3	4	mean	1	2	3	4	mean	
Dice	88.075	85.074	76.997	54.502	76.162	80.481	77.577	88.778	61.513	77.087	64.175	83.632	67.529	43.325	64.665	82.431	85.277	71.514	57.243	74.116	73.008
HD	1.079	3.088	0.962	0.753	1.471	2.496	6.427	1.222	1.615	2.940	2.153	3.847	1.758	1.206	2.241	1.871	3.743	1.430	1.187	2.058	2.178
JC	0.159	0.202	0.107	0.059	0.132	0.324	0.437	0.266	0.153	0.295	0.129	0.223	0.103	0.054	0.127	0.205	0.318	0.147	0.088	0.190	0.186
RVE	0.069	0.124	0.139	0.202	0.133	0.319	2.028	0.122	0.302	0.693	0.346	0.242	0.291	0.381	0.315	0.141	0.119	0.172	0.198	0.158	0.325
Sen	0.857	0.858	0.750	0.534	0.750	0.740	0.777	0.880	0.595	0.748	0.601	0.860	0.658	0.435	0.639	0.802	0.873	0.705	0.575	0.738	0.719
Spe	1.000	0.997	1.000	1.000	0.999	1.000	0.929	0.999	0.999	0.982	0.999	0.994	0.999	1.000	0.998	0.999	0.994	0.999	1.000	0.998	0.994
(8)	1	2	3	4	mean	1	2	3	4	mean	1	2	3	4	mean	1	2	3	4	mean	
Dice	89.468	86.396	72.558	55.431	75.963	72.648	78.573	93.068	68.255	78.136	55.325	88.577	70.853	45.212	64.992	78.437	86.464	74.982	60.753	75.159	73.563
HD	1.017	2.511	1.138	0.887	1.388	3.120	6.008	1.110	1.641	2.970	1.965	2.464	2.028	1.253	1.927	1.777	2.488	1.454	1.236	1.739	2.006
JC	0.162	0.205	0.100	0.060	0.132	0.297	0.443	0.287	0.174	0.300	0.113	0.242	0.111	0.056	0.130	0.195	0.327	0.157	0.094	0.193	0.189
RVE	0.107	0.149	0.223	0.214	0.173	0.314	1.988	0.083	0.328	0.678	0.467	0.135	0.355	0.338	0.324	0.291	0.083	0.169	0.277	0.205	0.345
Sen	0.883	0.888	0.679	0.542	0.748	0.694	0.790	0.928	0.731	0.786	0.535	0.906	0.687	0.460	0.647	0.792	0.880	0.747	0.656	0.769	0.738
Spe	1.000	0.997	1.000	1.000	0.999	0.999	0.932	1.000	0.999	0.983	1.000	0.996	0.999	1.000	0.999	0.999	0.996	1.000	0.999	0.998	0.995
(9)	1	2	3	4	mean	1	2	3	4	mean	1	2	3	4	mean	1	2	3	4	mean	
Dice	89.607	86.188	75.868	56.591	77.064	72.590	78.745	93.043	68.564	78.236	56.931	88.331	70.639	46.224	65.531	78.799	86.347	75.005	61.218	75.342	74.043
HD	1.058	2.354	1.184	0.933	1.382	2.856	6.023	1.083	1.636	2.899	1.922	2.545	1.874	1.239	1.895	1.758	2.562	1.427	1.221	1.742	1.980
JC	0.163	0.205	0.105	0.061	0.133	0.297	0.445	0.287	0.174	0.301	0.116	0.241	0.111	0.058	0.132	0.196	0.326	0.157	0.095	0.194	0.190
RVE	0.104	0.149	0.175	0.216	0.161	0.339	1.993	0.079	0.325	0.684	0.452	0.145	0.313	0.336	0.311	0.274	0.081	0.153	0.259	0.192	0.337
Sen	0.886	0.886	0.706	0.560	0.759	0.691	0.790	0.922	0.739	0.785	0.550	0.905	0.673	0.470	0.650	0.797	0.876	0.736	0.660	0.767	0.740
Spe	1.000	0.997	1.000	1.000	0.999	0.999	0.932	1.000	0.999	0.983	1.000	0.996	0.999	1.000	0.999	0.999	0.996	1.000	0.999	0.999	0.995

Organ indexes are numbers on the straight right side of the name of method.

Model performance is better when metrics are higher, except for HD and RVE.

Table S9. Hyper-parameter ablation for sUSAM.

Perturbation radius & Metric	Client index (the next line) & Organ index (after name of method)																				
	1					2					3					4					Mean
0.6	1	2	3	4	mean	1	2	3	4	mean	1	2	3	4	mean	1	2	3	4	mean	
Dice	88.789	84.057	73.915	52.409	74.793	72.391	72.412	91.661	67.626	76.022	62.499	86.911	70.869	42.980	65.815	81.889	85.269	74.171	60.902	75.558	73.047
HD	1.279	2.428	0.964	0.831	1.375	2.989	6.251	1.065	1.584	2.972	2.433	2.203	1.562	1.281	1.870	1.978	2.341	1.307	1.205	1.707	1.981
JC	0.160	0.198	0.101	0.056	0.129	0.295	0.407	0.281	0.173	0.289	0.126	0.238	0.109	0.053	0.131	0.204	0.321	0.155	0.095	0.194	0.186
RVE	0.147	0.148	0.192	0.211	0.174	0.353	1.980	0.104	0.233	0.668	0.357	0.129	0.258	0.360	0.276	0.309	0.086	0.133	0.236	0.191	0.327
Sen	0.905	0.865	0.680	0.500	0.737	0.699	0.713	0.903	0.684	0.750	0.620	0.859	0.669	0.422	0.643	0.849	0.843	0.725	0.643	0.765	0.724
Spe	0.999	0.997	1.000	1.000	0.999	0.999	0.931	1.000	0.999	0.982	0.999	0.997	1.000	1.000	0.999	0.999	0.997	1.000	1.000	0.999	0.995
0.7	1	2	3	4	mean	1	2	3	4	mean	1	2	3	4	mean	1	2	3	4	mean	
Dice	89.529	84.727	76.069	49.922	75.062	73.099	76.691	93.864	67.759	77.853	58.920	88.507	72.711	42.560	65.675	79.970	87.155	76.630	61.661	76.354	73.736
HD	1.033	2.749	0.948	0.785	1.379	2.841	6.141	1.002	1.530	2.879	2.256	2.312	1.641	1.158	1.842	1.853	2.404	1.296	1.133	1.671	1.943
JC	0.162	0.200	0.106	0.053	0.130	0.298	0.433	0.290	0.174	0.299	0.120	0.243	0.114	0.053	0.133	0.200	0.330	0.162	0.097	0.197	0.190
RVE	0.093	0.143	0.177	0.285	0.174	0.335	2.056	0.060	0.236	0.672	0.404	0.154	0.279	0.373	0.302	0.278	0.077	0.122	0.194	0.168	0.329
Sen	0.904	0.883	0.711	0.450	0.737	0.697	0.765	0.934	0.663	0.764	0.576	0.896	0.698	0.400	0.643	0.822	0.879	0.753	0.624	0.770	0.729
Spe	1.000	0.997	1.000	1.000	0.999	0.999	0.929	1.000	1.000	0.982	0.999	0.996	0.999	1.000	0.999	0.999	0.996	1.000	1.000	0.999	0.995
0.8	1	2	3	4	mean	1	2	3	4	mean	1	2	3	4	mean	1	2	3	4	mean	
Dice	89.563	81.818	73.000	54.332	74.678	71.880	77.954	92.785	67.677	77.574	58.873	87.737	70.434	44.521	65.391	80.880	87.264	75.637	60.457	76.060	73.426
HD	0.881	3.826	0.817	0.859	1.596	2.858	6.104	1.022	1.528	2.878	1.953	2.862	1.442	1.212	1.867	1.713	2.496	1.228	1.175	1.653	1.999
JC	0.163	0.191	0.101	0.059	0.128	0.292	0.441	0.286	0.173	0.298	0.120	0.239	0.109	0.056	0.131	0.203	0.330	0.159	0.095	0.197	0.189
RVE	0.071	0.278	0.220	0.207	0.194	0.380	2.094	0.075	0.247	0.699	0.416	0.195	0.260	0.366	0.309	0.224	0.081	0.141	0.173	0.155	0.339
Sen	0.880	0.881	0.677	0.508	0.737	0.678	0.779	0.914	0.660	0.758	0.567	0.895	0.670	0.424	0.639	0.822	0.885	0.737	0.620	0.766	0.725
Spe	1.000	0.996	1.000	1.000	0.999	0.999	0.928	1.000	0.999	0.982	1.000	0.995	1.000	1.000	0.998	0.999	0.996	1.000	1.000	0.999	0.995
0.9	1	2	3	4	mean	1	2	3	4	mean	1	2	3	4	mean	1	2	3	4	mean	
Dice	89.705	82.774	72.666	53.104	74.562	71.986	77.983	93.409	66.928	77.576	57.547	86.915	69.881	45.579	64.980	80.307	87.796	76.619	58.002	75.681	73.200
HD	0.910	2.553	0.971	0.898	1.333	2.815	6.092	1.023	1.570	2.875	1.937	2.840	1.652	1.274	1.926	1.698	2.559	1.250	1.215	1.680	1.954
JC	0.163	0.196	0.099	0.058	0.129	0.292	0.440	0.288	0.171	0.298	0.117	0.237	0.109	0.058	0.130	0.201	0.333	0.162	0.090	0.196	0.188
RVE	0.073	0.165	0.197	0.176	0.153	0.329	2.264	0.073	0.241	0.727	0.436	0.226	0.318	0.325	0.326	0.268	0.088	0.134	0.205	0.174	0.345
Sen	0.887	0.863	0.673	0.502	0.731	0.680	0.784	0.925	0.665	0.763	0.553	0.905	0.667	0.439	0.641	0.812	0.895	0.750	0.598	0.764	0.725
Spe	1.000	0.997	1.000	1.000	0.999	0.999	0.922	1.000	0.999	0.980	1.000	0.994	0.999	1.000	0.998	0.999	0.995	1.000	1.000	0.998	0.994

Start epoch & Metric	Client index (the next line) & Organ index (after name of method)																				
	1					2					3					4					Mean
200	1	2	3	4	mean	1	2	3	4	mean	1	2	3	4	mean	1	2	3	4	mean	
Dice	89.849	85.316	75.887	51.235	75.572	73.545	77.112	93.514	67.290	77.865	59.906	88.837	71.160	42.631	65.634	80.654	87.517	75.827	61.718	76.429	73.875
HD	1.013	2.652	1.073	0.810	1.387	2.795	6.135	1.041	1.543	2.878	2.216	2.409	1.781	1.224	1.907	1.814	2.443	1.357	1.164	1.694	1.967
JC	0.163	0.202	0.106	0.054	0.131	0.301	0.435	0.289	0.172	0.299	0.121	0.244	0.111	0.053	0.132	0.201	0.331	0.159	0.097	0.197	0.190
RVE	0.102	0.145	0.168	0.278	0.173	0.312	2.092	0.064	0.243	0.678	0.401	0.145	0.293	0.377	0.304	0.284	0.080	0.152	0.194	0.177	0.333
Sen	0.911	0.888	0.713	0.468	0.745	0.707	0.773	0.932	0.653	0.766	0.587	0.907	0.682	0.403	0.645	0.835	0.887	0.753	0.626	0.775	0.733
Spe	1.000	0.997	1.000	1.000	0.999	0.999	0.928	1.000	1.000	0.982	0.999	0.996	0.999	1.000	0.999	0.999	0.996	1.000	1.000	0.999	0.995
300	1	2	3	4	mean	1	2	3	4	mean	1	2	3	4	mean	1	2	3	4	mean	
Dice	89.529	84.727	76.069	49.922	75.062	73.099	76.691	93.864	67.759	77.853	58.920	88.507	72.711	42.560	65.675	79.970	87.155	76.630	61.661	76.354	73.736
HD	1.033	2.749	0.948	0.785	1.379	2.841	6.141	1.002	1.530	2.879	2.256	2.312	1.641	1.158	1.842	1.853	2.404	1.296	1.133	1.671	1.943
JC	0.162	0.200	0.106	0.053	0.130	0.298	0.433	0.290	0.174	0.299	0.120	0.243	0.114	0.053	0.133	0.200	0.330	0.162	0.097	0.197	0.190
RVE	0.093	0.143	0.177	0.285	0.174	0.335	2.056	0.060	0.236	0.672	0.404	0.154	0.279	0.373	0.302	0.278	0.077	0.122	0.194	0.168	0.329
Sen	0.904	0.883	0.711	0.450	0.737	0.697	0.765	0.934	0.663	0.764	0.576	0.896	0.698	0.400	0.643	0.822	0.879	0.753	0.624	0.770	0.729
Spe	1.000	0.997	1.000	1.000	0.999	0.999	0.929	1.000	1.000	0.982	0.999	0.996	0.999	1.000	0.999	0.999	0.996	1.000	1.000	0.999	0.995
400	1	2	3	4	mean	1	2	3	4	mean	1	2	3	4	mean	1	2	3	4	mean	
Dice	90.077	84.171	75.436	53.716	75.850	72.514	77.703	93.665	66.522	77.601	59.985	87.820	69.560	44.576	65.485	80.053	88.187	75.371	57.203	75.203	73.535
HD	0.859	3.096	0.835	0.920	1.428	2.822	6.115	1.004	1.546	2.872	1.893	2.686	1.691	1.289	1.890	1.724	2.586	1.320	1.205	1.708	1.975
JC	0.164	0.198	0.104	0.058	0.131	0.295	0.439	0.290	0.170	0.298	0.121	0.240	0.109	0.056	0.132	0.199	0.334	0.159	0.088	0.195	0.189
RVE	0.065	0.191	0.196	0.169	0.155	0.323	2.252	0.063	0.237	0.719	0.405	0.212	0.307	0.309	0.308	0.241	0.098	0.143	0.204	0.172	0.339
Sen	0.895	0.898	0.697	0.513	0.751	0.685	0.788	0.927	0.655	0.764	0.576	0.919	0.663	0.434	0.648	0.808	0.911	0.732	0.584	0.759	0.731
Spe	1.000	0.997	1.000	1.000	0.999	0.999	0.922	1.000	0.999	0.980	1.000	0.995	1.000	1.000	0.998	0.999	0.995	1.000	1.000	0.998	0.994

Organ indexes are numbers on the straight right side of the name of method.
Model performance is better when metrics are higher, except for HD and RVE.

Table S10. Comparison with SOTAs under setting 2.

Method & Metric	Client index (the next line) & Organ index (after name of method)																					
	1					2					3					4					Mean	Post
SOLO partial	1	2	3	4	mean	1	2	3	4	mean	1	2	3	4	mean	1	2	3	4	mean		
Dice	86.597	80.353	78.451	63.418	77.205	80.222	96.593	64.549	41.904	70.817	83.817	86.602	47.438	57.571	68.857	86.267	87.972	49.282	43.355	66.719	70.899	70.944
HD	0.869	1.916	0.705	0.704	1.049	2.536	2.312	2.006	2.215	2.267	1.111	2.640	1.463	0.941	1.539	1.414	2.383	1.599	1.246	1.661	1.629	1.449
JC	0.155	0.190	0.112	0.071	0.132	0.333	0.570	0.182	0.099	0.296	0.183	0.233	0.070	0.075	0.140	0.224	0.336	0.093	0.064	0.179	0.187	0.187
RVE	0.111	0.124	0.145	0.179	0.140	0.313	0.019	0.476	0.374	0.296	0.068	0.104	0.453	0.231	0.214	0.118	0.077	0.393	0.345	0.233	0.221	0.226
Sen	0.813	0.768	0.779	0.612	0.743	0.756	0.969	0.626	0.385	0.684	0.816	0.844	0.442	0.543	0.661	0.850	0.870	0.453	0.392	0.641	0.682	0.680
Spe	1.000	0.998	1.000	1.000	1.000	0.999	0.999	0.999	0.999	0.999	1.000	0.997	1.000	1.000	0.999	1.000	0.997	1.000	1.000	0.999	0.899	0.999
FedAvg*	1	2	3	4	mean	1	2	3	4	mean	1	2	3	4	mean	1	2	3	4	mean		
Dice	87.491	85.710	75.439	64.302	78.235	81.843	94.902	80.121	46.119	75.746	81.613	87.483	63.385	56.393	72.218	86.713	87.892	64.366	48.071	71.761	74.490	74.879
HD	0.835	1.930	0.885	0.773	1.106	2.422	2.583	1.635	2.234	2.218	1.334	2.658	1.652	1.234	1.720	1.627	2.473	1.549	1.277	1.731	1.694	1.426
JC	0.157	0.203	0.105	0.071	0.134	0.341	0.554	0.233	0.110	0.309	0.177	0.237	0.095	0.072	0.145	0.224	0.333	0.127	0.071	0.189	0.194	0.196
RVE	0.083	0.100	0.181	0.126	0.122	0.292	0.038	0.204	0.386	0.230	0.097	0.132	0.326	0.225	0.195	0.300	0.090	0.230	0.249	0.217	0.191	0.192
Sen	0.832	0.842	0.720	0.650	0.761	0.776	0.942	0.794	0.434	0.737	0.801	0.867	0.591	0.572	0.708	0.877	0.876	0.611	0.467	0.708	0.729	0.727
Spe	1.000	0.998	1.000	1.000	0.999	1.000	0.999	0.999	0.999	0.999	1.000	0.996	0.999	1.000	0.999	0.999	0.996	1.000	1.000	0.999	0.999	0.999
UFPS(Ours)	1	2	3	4	mean	1	2	3	4	mean	1	2	3	4	mean	1	2	3	4	mean		
Dice	86.947	84.396	80.748	61.723	78.454	86.442	94.902	89.666	45.739	79.187	84.747	87.565	72.425	57.972	75.677	87.138	87.976	69.928	51.247	74.072	76.848	77.678
HD	1.930	3.362	1.563	2.395	2.312	2.285	2.678	1.378	2.150	2.123	2.309	2.975	2.335	1.884	2.376	1.955	2.724	2.035	1.698	2.103	2.229	1.454
JC	0.156	0.199	0.115	0.068	0.134	0.362	0.553	0.270	0.108	0.323	0.184	0.238	0.111	0.075	0.152	0.226	0.334	0.142	0.077	0.195	0.201	0.204
RVE	0.119	0.160	0.188	0.581	0.262	0.317	0.039	0.122	0.390	0.217	0.117	0.122	0.382	0.361	0.245	0.423	0.088	0.272	0.236	0.255	0.245	0.186
Sen	0.851	0.852	0.805	0.605	0.778	0.824	0.957	0.901	0.412	0.774	0.853	0.884	0.720	0.599	0.764	0.889	0.887	0.715	0.488	0.745	0.765	0.763
Spe	0.999	0.997	1.000	0.999	0.999	1.000	0.998	0.999	0.999	0.999	0.999	0.996	0.999	0.999	0.998	0.999	0.996	0.999	0.999	0.998	0.999	0.999

Organ indexes are numbers on the straight right side of the name of method.
Model performance is better when metrics are higher, except for HD and RVE.

Table S11. Comparison with SOTAs under setting 3.

Method & Metric	Client index (the next line) & Organ index (after name of method) 1					2					3					4					Mean	Post
SOLO partial	1	2	3	4	mean	1	2	3	4	mean	1	2	3	4	mean	1	2	3	4	mean		
Dice	83.130	91.808	66.233	71.113	78.071	94.319	77.109	77.739	24.397	68.391	67.871	85.653	81.382	22.628	64.384	86.879	83.395	69.573	26.200	66.512	69.339	69.693
HD	1.003	1.312	1.387	0.644	1.087	1.629	5.959	2.638	2.345	3.143	1.544	2.322	1.735	1.376	1.744	1.506	2.871	1.853	1.306	1.884	1.964	1.706
JC	0.150	0.225	0.086	0.083	0.136	0.413	0.424	0.221	0.056	0.279	0.147	0.230	0.126	0.027	0.133	0.225	0.308	0.141	0.037	0.178	0.181	0.183
RVE	0.112	0.059	0.366	0.135	0.168	0.056	0.842	0.844	0.547	0.572	0.318	0.141	0.318	0.551	0.332	0.480	0.098	0.267	0.574	0.355	0.357	0.341
Sen	0.816	0.930	0.633	0.693	0.768	0.946	0.762	0.908	0.223	0.710	0.679	0.873	0.806	0.204	0.641	0.901	0.833	0.709	0.230	0.668	0.697	0.694
Spe	1.000	0.999	0.999	1.000	1.000	1.000	0.968	0.996	0.999	0.991	1.000	0.997	0.999	1.000	0.999	0.999	0.996	0.999	1.000	0.999	0.997	0.997
FedAvg*	1	2	3	4	mean	1	2	3	4	mean	1	2	3	4	mean	1	2	3	4	mean		
Dice	86.362	90.381	77.539	65.820	80.026	92.694	79.490	83.124	25.580	70.222	73.052	86.870	78.673	30.003	67.150	86.030	85.495	69.783	28.162	67.368	71.192	71.523
HD	1.035	2.080	1.617	0.691	1.356	1.814	5.631	2.175	2.319	2.985	1.864	2.458	1.923	1.286	1.883	1.649	2.938	1.924	1.389	1.975	2.050	1.685
JC	0.155	0.220	0.107	0.075	0.139	0.401	0.438	0.244	0.059	0.286	0.157	0.234	0.122	0.036	0.137	0.221	0.318	0.142	0.040	0.180	0.186	0.187
RVE	0.093	0.093	0.324	0.157	0.167	0.081	0.831	0.605	0.493	0.503	0.240	0.139	0.387	0.451	0.304	0.401	0.099	0.302	0.427	0.307	0.320	0.294
Sen	0.853	0.919	0.779	0.650	0.800	0.929	0.784	0.917	0.237	0.717	0.734	0.891	0.780	0.279	0.671	0.907	0.864	0.711	0.262	0.686	0.719	0.716
Spe	1.000	0.998	0.999	1.000	0.999	0.999	0.969	0.997	0.999	0.991	0.999	0.996	0.999	1.000	0.999	0.999	0.995	0.999	1.000	0.998	0.997	0.997
UFPS(Ours)	1	2	3	4	mean	1	2	3	4	mean	1	2	3	4	mean	1	2	3	4	mean		
Dice	84.849	83.967	77.615	67.276	78.427	92.129	82.454	84.455	32.139	72.794	77.418	85.045	74.852	35.710	68.256	83.735	86.671	69.981	30.618	67.751	71.807	72.601
HD	3.194	3.500	2.875	1.176	2.686	1.879	6.047	1.954	2.418	3.075	3.587	3.235	2.678	2.000	2.875	2.736	3.392	2.261	1.857	2.562	2.800	1.951
JC	0.150	0.197	0.107	0.076	0.132	0.397	0.461	0.251	0.073	0.296	0.163	0.227	0.114	0.043	0.137	0.212	0.324	0.142	0.044	0.180	0.186	0.190
RVE	0.503	0.374	0.910	0.172	0.490	0.103	1.363	0.587	0.504	0.639	0.350	0.229	0.591	0.293	0.366	0.712	0.121	0.296	0.363	0.373	0.467	0.366
Sen	0.869	0.921	0.787	0.672	0.812	0.923	0.830	0.904	0.290	0.737	0.794	0.899	0.767	0.348	0.702	0.898	0.900	0.716	0.296	0.703	0.739	0.735
Spe	0.998	0.995	0.998	1.000	0.998	0.999	0.952	0.997	0.999	0.987	0.998	0.994	0.998	0.999	0.997	0.998	0.994	0.999	0.999	0.997	0.995	0.996

Organ indexes are numbers on the straight right side of the name of method.
Model performance is better when metrics are higher, except for HD and RVE.

Table S12. Performance comparison between teacher models.

No aug	Kidney	Liver	Spleen	Pancreas	Mean
Client 1	91.43 / 0.77	86.71 / 1.44	0 / 1.61	0.51 / 1.11	47.01 / 2.23
Client 2	51.41 / 3.66	33.29 / 7.83	93.64 / 0.93	70.59 / 1.53	62.23 / 3.49
Client 3	43.56 / 2.81	93.73 / 1.71	0.21 / 2.19	10.73 / 1.40	37.06 / 2.03
Client 4	55.40 / 2.27	90.54 / 2.08	0.99 / 2.84	29.51 / 1.51	44.11 / 2.18
Mean	60.45 / 2.37	76.06 / 3.26	23.41 / 1.89	27.83 / 1.38	47.01 / 2.23
**Aug**	**Kidney**	**Liver**	**Spleen**	**Pancreas**	**Mean**
Client 1	91.48 / 0.80	85.92 / 1.21	53.94 / 1.22	45.35 / 0.85	69.17 / 1.02
Client 2	68.78 / 3.02	72.38 / 6.34	91.84 / 1.05	70.01 / 1.53	75.75 / 3.03
Client 3	53.09 / 2.11	90.20 / 1.74	64.66 / 1.27	34.43 / 1.35	60.59 / 1.62
Client 4	77.48 / 1.57	86.16 / 2.28	75.39 / 1.12	59.76 / 1.11	74.70 / 1.52
Mean	72.70 / 1.87	83.66 / 2.89	71.45 / 1.16	52.38 / 1.21	70.05 / 1.80
**Aug+DP**	**Kidney**	**Liver**	**Spleen**	**Pancreas**	**Mean**
Client 1	90.79 / 0.70	83.76 / 1.20	46.84 / 0.98	38.20 / 0.76	64.89 / 0.91
Client 2	51.93 / 3.42	68.73 / 5.96	86.12 / 1.17	68.12 / 1.52	68.72 / 3.01
Client 3	40.64 / 1.96	88.58 / 1.65	59.30 / 1.15	27.10 / 1.22	53.90 / 1.49
Client 4	70.59 / 1.63	84.50 / 2.26	72.73 / 1.08	55.26 / 1.07	70.77 / 1.51
Mean	63.48 / 1.92	81.39 / 2.76	66.24 / 1.09	47.17 / 1.14	64.57 / 1.73

Here we show Dice / HD (higher / lower numbers are better) on test sets.

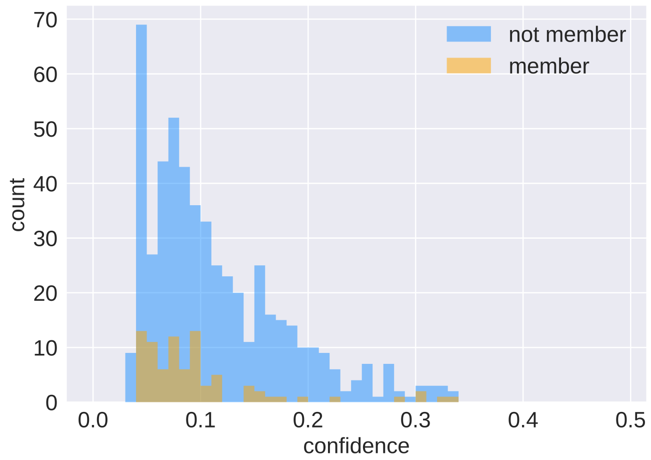

(a) teacher 1, no aug

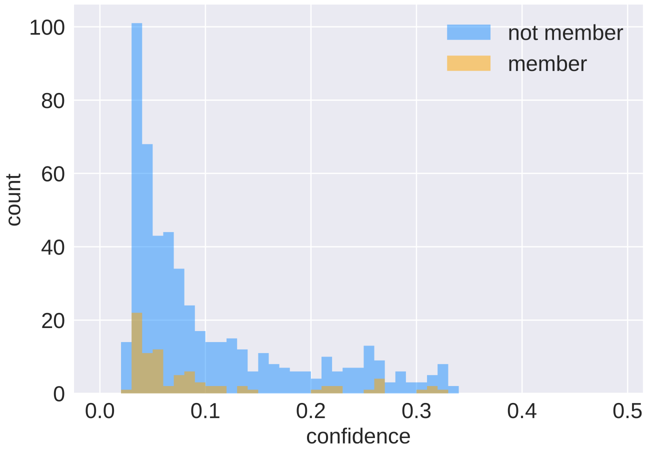

(b) teacher 1, aug

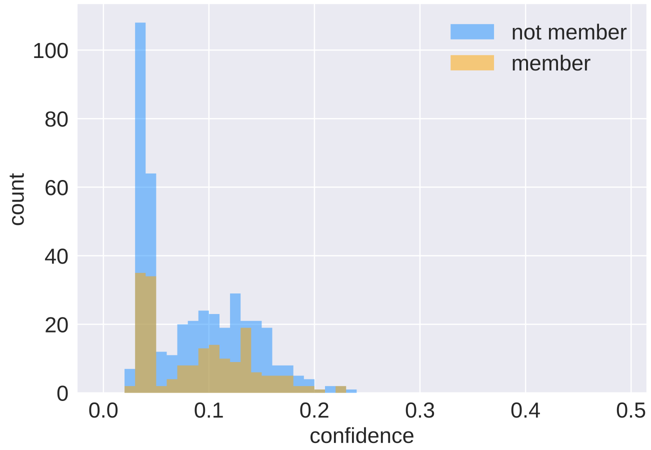

(c) teacher 2, no aug

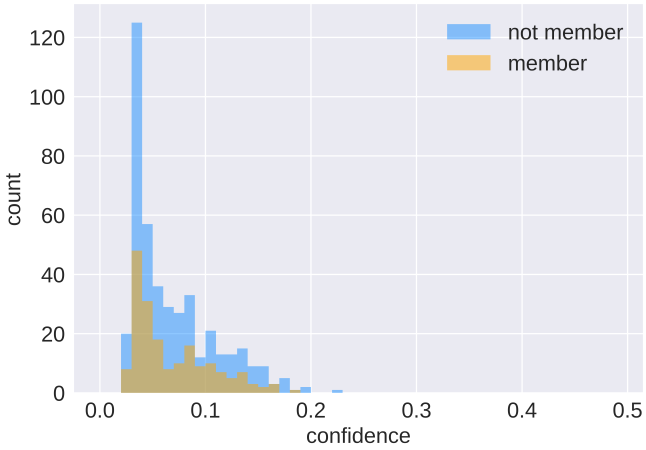

(d) teacher 2, aug

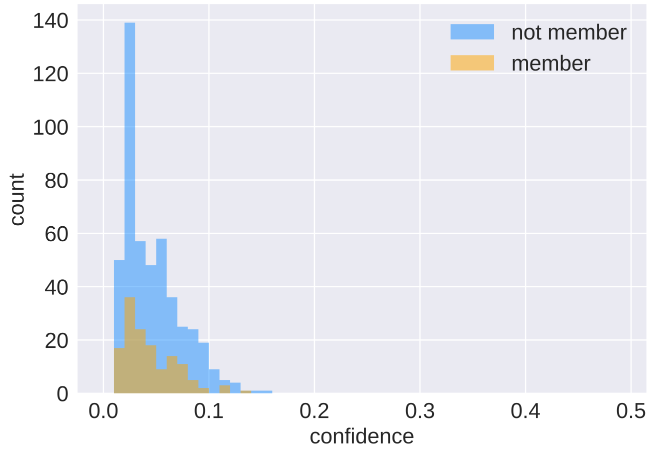

(e) teacher 3, no aug

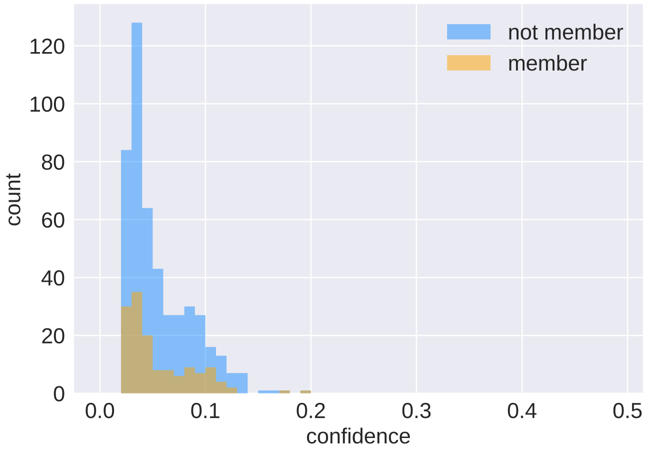

(f) teacher 3, aug

Figure S 5: Comparison of uncertainty.
Comparison of uncertainty for all clients when teacher models are trained with or without augmentation (aug).

Table S13. MIA attack performance on teacher models.

No aug	Client 1	Client 2	Client 3	Mean
Shadow	50.39 / 2.588	60.36 / 1.115	71.43 / 5.189	60.72 / 2.964
Attack class mean	68.06 / 57.75	69.61 / 66.11	53.05 / 36.40	63.57 / 53.42
Attack class 1	82.92 / 48.22	97.87 / 74.49	98.63 / 59.01	93.14 / 60.57
**Aug**	**Client 1**	**Client 2**	**Client 3**	**Mean**
Shadow	64.45 / 2.614	46.29 / 1.304	82.21 / 5.760	64.31 / 3.226
Attack class mean	78.32 / 78.56	68.12 / 67.18	76.23 / 72.74	74.22 / 72.82
Attack class 1	65.85 / 66.66	46.80 / 59.06	94.52 / 73.79	69.05 / 66.50
**Aug + DP**	**Client 1**	**Client 2**	**Client 3**	**Mean**
Shadow	59.11 / 2.722	45.46 / 1.396	80.72 / 5.465	61.76 / 3.194
Attack class mean	70.01 / 66.91	69.28 / 68.73	57.29 / 45.82	65.52 / 60.48
Attack class 1	63.41 / 51.99	51.06 / 61.93	95.89 / 60.86	70.12 / 58.26

Here we show Dice / HD (higher / lower numbers indicate better stimulation) for the shadow model and accuracy / F1 score (higher numbers indicate better attacks) for the attack model.
Class 1 refers to the label 'member'.

References

1. Bartlett, P. L. and Mendelson, S. (2001). Rademacher and gaussian complexities: Risk bounds and structural results. In *Computational Learning Theory: 14th Annual Conference on Computational Learning Theory, COLT 2001 and 5th European Conference on Computational Learning Theory, EuroCOLT 2001 Amsterdam, The Netherlands, July 16–19, 2001 Proceedings 14*, pages 224–240. Springer. https://doi.org/10.1007/3-540-44581-1_15.
2. Cardoso, M. J., Li, W., Brown, R., Ma, N., Kerfoot, E., Wang, Y., Murrey, B., Myronenko, A., Zhao, C., Yang, D., et al. (2022). Monai: An open-source framework for deep learning in healthcare. *arXiv preprint arXiv:2211.02701*. https://arxiv.org/abs/2211.02701.
3. Chobola, T., Usynin, D., and Kaissis, G. (2022). Membership inference attacks against semantic segmentation models. *arXiv preprint arXiv:2212.01082*. https://arxiv.org/abs/2212.01082.
4. Dwork, C. (2006). Differential privacy. In *International colloquium on automata, languages, and programming*, pages 1–12. Springer. https://link.springer.com/chapter/10.1007/11787006_1.
5. Grill, J.-B., Strub, F., Altché, F., Tallec, C., Richemond, P., Buchatskaya, E., Doersch, C., Avila Pires, B., Guo, Z., Gheshlaghi Azar, M., et al. (2020). Bootstrap your own latent-a new approach to self-supervised learning. *Advances in neural information processing systems*, 33:21271–21284. https://proceedings.neurips.cc/paper_files/paper/2020/file/f3ada80d5c4ee70142b17b8192b2958e-Paper.pdf.
6. Isensee, F., Jaeger, P. F., Kohl, S. A., Petersen, J., and Maier-Hein, K. H. (2021). nnu-net: a self-configuring method for deep learning-based biomedical image segmentation. *Nature methods*, 18(2):203–211. https://doi.org/10.1038/s41592-020-01008-z.
7. Kornblith, S., Norouzi, M., Lee, H., and Hinton, G. (2019). Similarity of neural network representations revisited. In *International Conference on Machine Learning*, pages 3519–3529. PMLR. http://proceedings.mlr.press/v97/kornblith19a/kornblith19a.pdf.
8. Loshchilov, I. and Hutter, F. (2017). Decoupled weight decay regularization. *arXiv preprint arXiv:1711.05101*. https://arxiv.org/abs/1711.05101.
9. Mikosch, T., van der Vaart, A., and Wellner, J. A. (1996). *Weak Convergence and Empirical Processes: With Applications to Statistics*. Springer Science & Business Media. https://link.springer.com/book/10.1007/978-1-4757-2545-2.
10. Qu, Z., Li, X., Duan, R., Liu, Y., Tang, B., and Lu, Z. (2022). Generalized federated learning via sharpness aware minimization. In *International Conference on Machine Learning*, pages 18250–18280. PMLR. https://proceedings.mlr.press/v162/qu22a.html.
11. Shokri, R., Stronati, M., Song, C., and Shmatikov, V. (2017). Membership inference attacks against machine learning models. In *2017 IEEE symposium on security and privacy (SP)*, pages 3–18. IEEE. https://doi.org/10.1109/SP.2017.41.
12. Sinha, A., Namkoong, H., and Duchi, J. (2017). Certifiable distributional robustness with principled adversarial training. *arXiv preprint arXiv:1710.10571*, 2. https://arxiv.org/abs/1710.10571.
13. Yang, H., Fang, M., and Liu, J. (2021). Achieving linear speedup with partial worker participation in non-iid federated learning. *arXiv preprint arXiv:2101.11203*. https://arxiv.org/abs/2101.11203.
